# Substantial decrease in CO_2_ emissions from Chinese inland waters due to global change

**DOI:** 10.1038/s41467-021-21926-6

**Published:** 2021-03-19

**Authors:** Lishan Ran, David E. Butman, Tom J. Battin, Xiankun Yang, Mingyang Tian, Clément Duvert, Jens Hartmann, Naomi Geeraert, Shaoda Liu

**Affiliations:** 1 Department of Geography, The University of Hong Kong, Pok Fu Lam Road, Hong Kong; 2 School of Environmental and Forest Sciences, University of Washington, Seattle, WA USA; 3 Stream Biofilm and Ecosystem Research Laboratory, School of Architecture, Civil and Environmental Engineering, École Polytechnique Fédérale de Lausanne, Lausanne, Switzerland; 4 School of Geography and Remote Sensing, Guangzhou University, Guangzhou, China; 5 Research Institute for the Environment and Livelihoods, Charles Darwin University, Darwin, NT Australia; 6 Institute for Geology, Center for Earth System Research and Sustainability (CEN), Universität Hamburg, Hamburg, Germany; 7 School of Biological Sciences, The University of Hong Kong, Pok Fu Lam Road, Hong Kong; 8 State Key Laboratory of Water Environment Simulation and Modelling, School of Environment, Beijing Normal University, Beijing, China

**Keywords:** Carbon cycle, Climate change

## Abstract

Carbon dioxide (CO_2_) evasion from inland waters is an important component of the global carbon cycle. However, it remains unknown how global change affects CO_2_ emissions over longer time scales. Here, we present seasonal and annual fluxes of CO_2_ emissions from streams, rivers, lakes, and reservoirs throughout China and quantify their changes over the past three decades. We found that the CO_2_ emissions declined from 138 ± 31 Tg C yr^−1^ in the 1980s to 98 ± 19 Tg C yr^−1^ in the 2010s. Our results suggest that this unexpected decrease was driven by a combination of environmental alterations, including massive conversion of free-flowing rivers to reservoirs and widespread implementation of reforestation programs. Meanwhile, we found increasing CO_2_ emissions from the Tibetan Plateau inland waters, likely attributable to increased terrestrial deliveries of organic carbon and expanded surface area due to climate change. We suggest that the CO_2_ emissions from Chinese inland waters have greatly offset the terrestrial carbon sink and are therefore a key component of China’s carbon budget.

## Abstract (web summary)

Inland waters emit greenhouse gases, but robust estimations are hampered by a dearth of spatio-temporally resolved measurements. Here the authors present annual fluxes of CO_2_ from Chinese inland waters over the past several decades, showing that emission fluxes have significantly declined since the 80s.

## Introduction

Inland waters are an important component of the global carbon cycle and function as active reactors, transporting and transforming large quantities of naturally and anthropogenically derived carbon^1–4^. Today, we understand that the flux of carbon dioxide (CO_2_) outgassing, admittedly still poorly constrained, from inland waters is of the same order of magnitude as land–atmosphere and land–ocean net carbon exchanges^5,6^. While most efforts over the last decade have focused on refining these estimates at the regional and global scales^7–9^, we do not understand the responsiveness of regional CO_2_ emissions from inland waters to global change. Addressing this is fundamental because of the inherent feedbacks between the carbon cycle and the Earth’s climate.

Comprehensive and credible estimates of global CO_2_ evasion from inland waters can only be achieved when data with high spatial and temporal coverage, including the accurate quantification of inland water surface area, are available. Current estimates have been greatly refined; however, recent studies demonstrate that they are subjected to upward revisions because key regions, such as China, have not been properly included^6,9^. Although China occupies only ~7% of the global land surface, it includes some of the largest rivers and hosts half of the world’s reservoirs^10,11^. Meanwhile, its lakes are globally significant and have been undergoing widespread changes^12,13^. Better understanding of CO_2_ evasion from China’s inland waters is therefore essential to constrain global estimates and clarify the importance of inland waters in regional and global carbon budgets.

An unprecedented economic development in China over the past decades has impacted the environment and thereby profoundly modified the carbon dynamics of the country’s terrestrial landscapes^14,15^. Most carbon cycling studies in China have focused on balancing the carbon sequestration and emissions in terrestrial ecosystems^15,16^, whereas research on lateral carbon export to inland waters and its subsequent metabolism and evasion as CO_2_ is largely missing. Recent studies have shown that human activities have altered the aquatic carbon dynamics in China^14,17,18^, but how this may affect the CO_2_ evasion from China’s inland waters remains unknown.

In this study, we quantify and compare CO_2_ emissions from streams, rivers, lakes, and reservoirs in China in the 1980s and 2010s, during which China experienced unprecedented environmental and socioeconomic changes. The first period refers to the 1980s prior to massive anthropogenic perturbations, while the second period (the 2010s) is posterior to extensive damming and intensive land-use change. The use of an unprecedented spatiotemporal dataset (see Methods for further details) enabled us to reconstruct past perturbations caused by rapid environmental and socioeconomic changes, which might have happened in other areas of the world going through comparable changes.

## Results and discussion

### Inland water surface area changes

We quantified the inland water surface area across China for the two time periods. Given the strong hydrologic seasonality of the East Asian monsoon climate, we separately calculated the inland water surface area for the dry and wet seasons (Supplementary Section 1). Total surface area of Chinese streams and rivers in the 1980s was 55,488 ± 12,886 km^2^ during the dry season and 65,076 ± 14,357 km^2^ during the wet season, accounting for 0.58–0.68% of the total land surface of China. Regional stream surface area estimates ranged from 0.16% to 1.54% of the total watershed area (Supplementary Table 4.3). In comparison, the stream surface area in the 2010s declined to 51,003 ± 14,439 km^2^ in the dry season and 58,279 ± 15,027 km^2^ in the wet season, indicating an 8.1−10.4% decline relative to the 1980s. All regions except the Tibetan Plateau showed a decreasing trend, largely due to combined effects of climate change and of increasing water withdrawals and damming that transformed free-flowing rivers to reservoirs^19^. The stream surface area on the Tibetan Plateau increased by 8.5% and 2.5% in the dry and wet seasons, respectively. This reflects the expansion of the stream networks and increasing flow due to melting glaciers, snow, and permafrost and increasing precipitation in the region^20,21^. Furthermore, these streams and rivers remain largely unaffected by damming and other human alterations. For all six regions, there was a statistically significant correlation between stream surface area, expressed as a percentage of land surface, and precipitation rate in both periods (Supplementary Fig. 4.6).

We estimated the surface area of lakes and reservoirs by using a combination of satellite image-based delineation and national inventories (Supplementary Section 4). The total surface area of lakes was 79,196 ± 6415 and 82,570 ± 6688 km^2^ in the 1980s and 2010s, respectively, consistent with recent studies on temporal changes in Chinese lakes^12,20^. The spatial distribution of lakes exhibited substantial variations with more than half of the surface area located on the Tibetan Plateau although this region comprises only 27% of China’s land surface. Lake shrinkage occurred in eastern China because of land reclamation^20^, whereas the lakes in western China, including the Tibetan Plateau and NW China, expanded significantly over the past three decades (Supplementary Table 4.11). Similar to the expanding stream networks, this is attributable to the melting glaciers, permafrost thawing and increasing precipitation^21–23^. It is evident that the surface area of Tibetan Plateau lakes could further increase with projected climate warming^20,22^.

China has engaged in a dam boom since the 1980s with surging economy that spurred the need for energy and food production (Supplementary Fig. 4.7). With ≈15,000 new reservoirs completed between the two periods, the storage capacity of reservoirs was more than double. Accordingly, the reservoir surface area increased from 14,772 ± 1196 km^2^ in the 1980s to 25,616 ± 2075 km^2^ in the 2010s. This corresponds to a 73% increase, the largest temporal change among the three inland water types. If lakes and reservoirs are combined, our estimates reveal that the lentic ecosystems account for 61–66% of the total surface area of Chinese inland waters. Overall, the total surface area of Chinese inland waters increased by about 8600 km^2^ on average (6%) in the 2010s compared with the 1980s. Therefore, the reduction in stream surface area between the two periods has been offset by the simultaneous expansion of lakes and reservoirs.

### Regional variability in areal CO_2_ evasion

Chinese inland waters are generally supersaturated with CO_2_ with respect to the atmosphere. The partial pressure of CO_2_ (*p*CO_2_) in streams and rivers ranged from 112 to 29,096 µatm (mean: 2798 ± 1906 µatm) and tended to decline downstream (Supplementary Fig. 6.1). Median stream and river *p*CO_2_ varied from 1168 to 3589 µatm across the six regions. The *p*CO_2_ results were 0.8–5.9 times (on average 3.0 times) higher than those estimated by Raymond et al.^7^ and Lauerwald et al.^8^ for the same regions. This suggests that using a very limited water chemistry database^7^ or using a statistical model neglecting headwater streams^8^ for East Asia would result in significant underestimations of riverine *p*CO_2_. We treated reservoirs as similar to natural lakes because their elevated CO_2_ emissions during the initial years after impoundment show an exponential decline with increasing reservoir age^7,24^. Lake and reservoir *p*CO_2_ varied from <50 to 6809 µatm, and the average of median *p*CO_2_ values ranged between 1044 and 3552 µatm across regions. However, it is important to note that 21% of the lake and reservoir sampling locations were undersaturated with CO_2_.

**Fig. 1:**
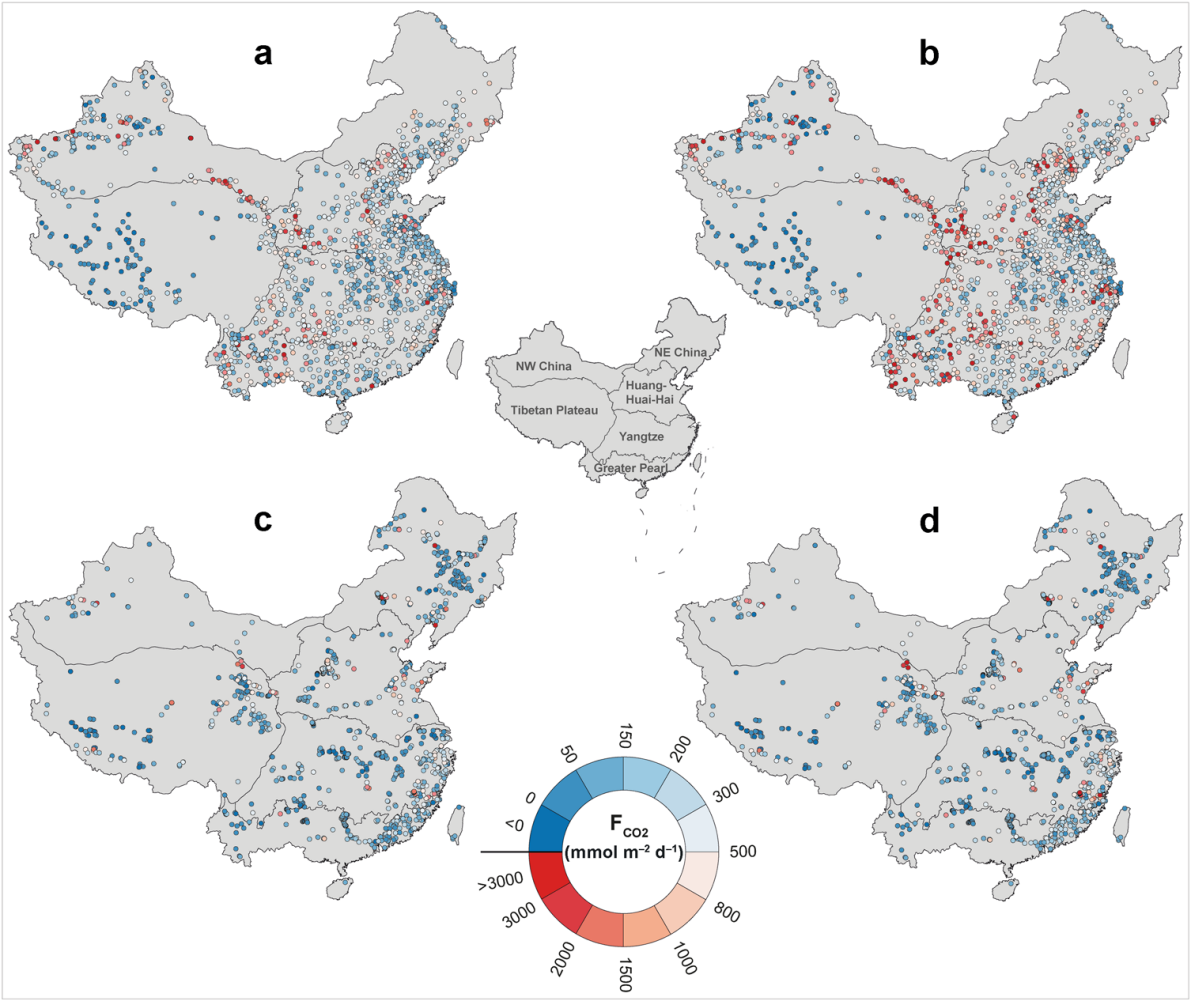
Spatial variations in CO_2_ efflux from inland waters across China. Dry season (**a**) and wet season (**b**) in the 1980s, and dry season (**c**) and wet season (**d**) in the 2010s. The names correspond to the regions discussed in the text (Supplementary Section 1).

**Fig. 2:**
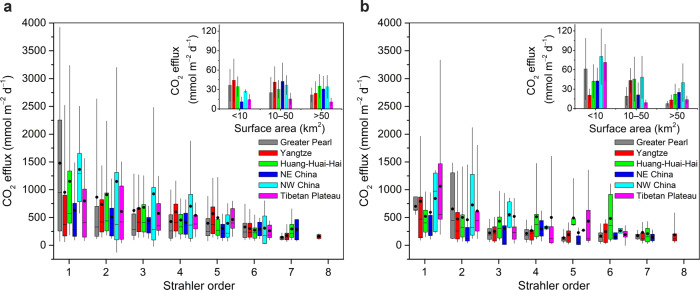
Spatial distribution of CO_2_ effluxes across Strahler orders of Chinese stream network. **a** 1980s. **b** 2010s. The box spans the interquartile range, the circle denotes the mean, the line denotes the median and the whiskers represent the 10th and 90th percentiles. Also shown in the inset graph are CO_2_ effluxes from lakes and reservoirs in the 1980s (a) and 2010s (b) for comparison. Error bars represent mean ± standard deviation.

We calculated the areal CO_2_ efflux (F_CO2_) from streams and rivers using different approaches (see Methods for further details) and found pronounced spatial and temporal variations (Fig. 1). Highest F_CO2_ values were measured in the Huang-Huai-Hai and NW China regions, which are situated in a dry climate with strong chemical weathering and are hydrologically controlled by groundwater that sustains high *p*CO_2_ values^25,26^. The F_CO2_ decreased downstream as streams and rivers became larger (according to their Strahler order; see Methods) across all regions, showing an average decrease of 75−123 mmol m^−2^ d^−1^ with increasing Strahler order (Fig. 2). Recent studies indicated that small streams typically have high *p*CO_2_ levels because of their high hydrological connectivity with terrestrial landscapes^27–29^. This decreasing trend is consistent with the rapid degassing of CO_2_ in small streams driven by high gas transfer velocities. Our F_CO2_ estimates were 22–48% higher in the wet season than in the dry season, which reveals stronger terrestrial inputs of carbon or soil CO_2_ due to better connected flow paths and in-stream CO_2_ production by microbial respiration^30,31^. Stronger turbulence caused by increased flow velocities and discharge in the wet season may have also supported the higher F_CO2_. Overall, the dry and wet season F_CO2_ in the 2010s has declined by about 13% and 32%, respectively, compared to that in the 1980s. The mean annual F_CO2_ declined from 630 mmol m^−2^ d^−1^ in the 1980s to 348 mmol m^−2^ d^−1^ in the 2010s, representing a decrease of 45% (Supplementary Table 6.1). We tested the robustness of this declining trend by comparing the CO_2_ evasion at sampling sites (*n* = 91) where F_CO2_ was available for both periods, which yielded a similar decrease between the two periods (Supplementary Section 7). These F_CO2_ estimates are comparable to those in the conterminous United States^32^ with similar climates as in China, but are lower than estimates for tropical African rivers^5^.

Compared with streams and rivers, lakes and reservoirs had substantially lower CO_2_ emissions at 23 ± 21 and 27 ± 66 mmol m^−2^ d^−1^ in the 1980s and 2010s, respectively (Fig. 2). The F_CO2_ estimates for lakes and reservoirs generally declined with increasing size across regions (Supplementary Table 6.3). This is because small lakes and reservoirs with strong land–water connectivity receive higher loads of terrestrial carbon and dissolved CO_2_ relative to water volume^33^. In situ CO_2_ production by degradation of terrestrial carbon coupled with direct CO_2_ inputs through inflowing streams likely resulted in high F_CO2_ values^34,35^.

### CO_2_ evasion from Chinese inland waters

**Fig. 3:**
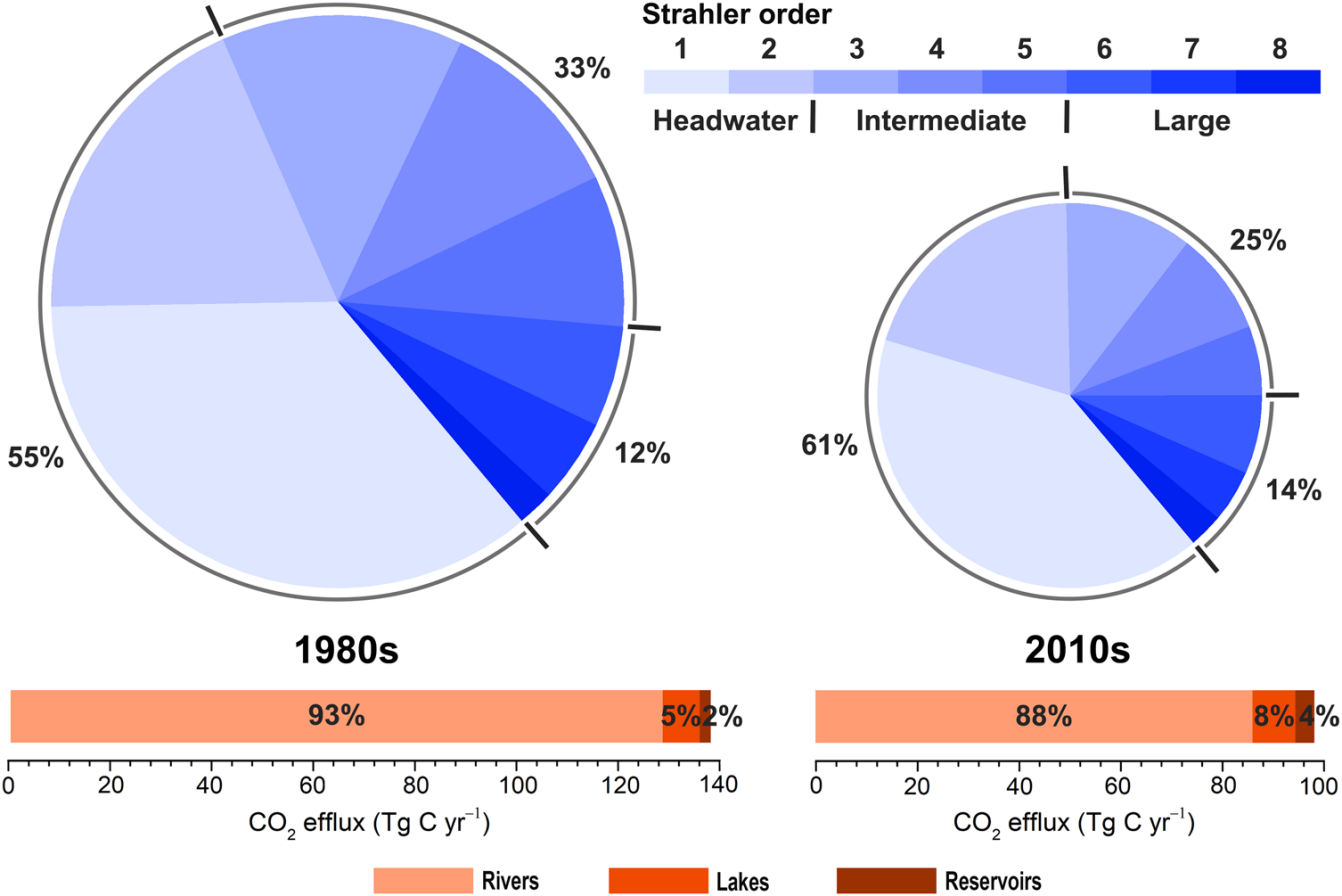
Comparison of CO_2_ emissions from Chinese inland waters in the 1980s and 2010s. Upper: relative importance of different Strahler order streams in riverine CO_2_ evasion. Lower: relative contributions of rivers, lakes, and reservoirs in total CO_2_ efflux. The sizes of the pie charts are proportional to the riverine CO_2_ evasion flux. Percentage contributions are given for headwater (first–second orders), intermediate (third–fifth orders), and large (sixth–eighth orders) streams.

We estimated the total efflux from the streams and rivers across China at 128.6 ± 31.3 Tg C yr^−1^ in the 1980s and 85.8 ± 19.4 Tg C yr^−1^ in the 2010s (Table 1; Fig. 3). Such values are of the same order of magnitude as the integrated flux for streams and rivers in the conterminous United States of 97 Tg C yr^−1^ (ref. ^32^) or one third of the estimate for African rivers with 270–370 Tg C yr^−1^ (ref. ^5^). The headwater streams made a disproportionately high contribution, accounting for 55% of the total riverine efflux in the 1980s (Fig. 3). This percentage increased to 61% in the 2010s (Fig. 3) as intermediate and large rivers were progressively dammed and converted to reservoirs as testified by the concomitantly increasing reservoir number and storage capacity (Supplementary Fig. 4.7). Although headwater streams comprise only 34–38% of the total stream surface area (Supplementary Section 6), their disproportionate importance in the total riverine efflux is an increasingly common observation worldwide^28,32,36^. We conclude that accurate determination of CO_2_ emissions from headwater streams is critical for evaluating the relative importance of streams and rivers in the global carbon cycle.

**Table 1:** Changes in CO_2_ effluxes from Chinese inland waters from the 1980s to the 2010s.

Region	1980s			2010s		
	Rivers	Lakes	Reservoirs	Rivers	Lakes	Reservoirs
	(Tg C yr^−1^)	(Tg C yr^−1^)	(Tg C yr^−1^)	(Tg C yr^−1^)	(Tg C yr^−1^)	(Tg C yr^−1^)
Greater Pearl	23.8 (12.7)	0.2 (0.1)	0.3 (0.1)	12.2 (4.6)	0.1 (0.1)	0.8 (0.5)
Yangtze	46.0 (24.4)	2.1 (1.1)	0.7 (0.3)	29.9 (15.3)	1.1 (0.4)	1.0 (0.3)
Huang-Huai-Hai	15.7 (8.0)	0.9 (0.2)	0.5 (0.1)	9.2 (3.8)	0.8 (0.4)	0.8 (0.4)
NE China	16.0 (5.4)	1.3 (0.2)	0.5 (0.1)	11.0 (6.1)	1.4 (0.3)	0.6 (0.2)
NW China	11.2 (8.5)	0.7 (0.2)	0.1 (0.02)	4.9 (2.3)	1.2 (0.5)	0.4 (0.1)
Tibetan Plateau	15.8 (7.4)	2.1 (1.0)	0.03 (0.01)	18.6 (7.9)	3.8 (1.1)	0.07 (0.02)
Subtotal	128.6 (31.3)	7.3 (1.5)	2.1 (0.3)	85.8 (19.4)	8.4 (1.4)	3.7 (0.7)
Total	138 (31)			98(19)		

Numbers in parentheses represent standard deviation.

For lakes and reservoirs, our estimate resulted in an evasion of 9.4 and 12.1 Tg C yr^−1^ in the 1980s and 2010s, respectively (Table 1). These values are less than the most recent estimate of ~25 Tg C yr^−1^ (ref. ^11^). However, our estimates represent seasonal variations of F_CO2_ and its size dependence that shows generally lower evasion rates in large water surfaces. Our estimated F_CO2_ per size class also compares well with a recent global study which evaluated CO_2_ evasion on the basis of direct measurements^33^.

Combining our results, we found that the sum of integrated effluxes across regions has decreased from 138 ± 31 Tg C yr^−1^ (91−200 Tg C yr^−1^; 5th and 95th confidence interval percentiles) in the 1980s to 98 ± 19 Tg C yr^−1^ (66−136 Tg C yr^−1^; 5th and 95th confidence interval percentiles) in the 2010s (Table 1), of which 88–93% was emitted from streams and rivers (Fig. 3). This suggests an overall decrease of 29% over the past three decades. The Tibetan Plateau is the only region showing increased evasion from streams/rivers, lakes and reservoirs, with riverine and lake CO_2_ efflux increasing by 18 and 81%, respectively. In comparison, the riverine CO_2_ evasion in all other regions presents strong declines of 31−56% (Table 1; Supplementary Table 6.1). Likewise, the effluxes from lakes declined in the three largest catchments (Yangtze, Yellow (Huang), and Pearl) (Table 1; Supplementary Table 6.2). Our research suggests that conversion of flowing rivers to reservoirs which show physicochemical properties analogous to lakes caused a significant reduction of CO_2_ emissions. If streams/rivers and reservoirs are considered together, their combined efflux has been reduced by roughly one-third in the recent three decades largely due to impoundments across China. Yet, it is important to note that the lentic ecosystems after impoundment tend to create favorable environments for methane production and evasion^37^. Together with the increasing trophic status throughout Chinese lakes, quantifying this pathway will help refine the overall carbon evasion.

### Potential drivers for the decreasing CO_2_ effluxes

**Fig. 4:**
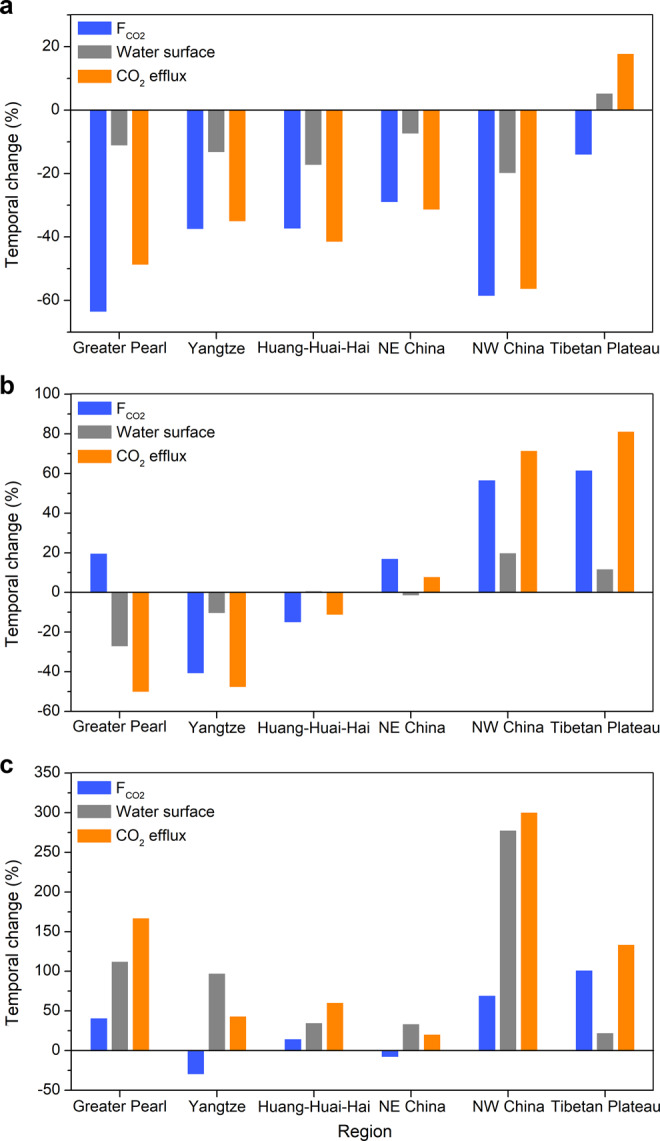
Changes in F_CO2_ and water surface area drive the temporal pattern of the total CO_2_ efflux across the six regions in China. **a** Rivers. **b** Lakes. **c** Reservoirs.

Both human and natural factors can affect CO_2_ emissions by changing the water surface area and/or F_CO2_, which in turn depends on surface water *p*CO_2_ and gas transfer velocity (Fig. 4). Land cover and land use changes have been recognized as important drivers of CO_2_ production and flux^1,36^. Significant forest cover increases were observed in the previously agriculture-dominated eastern China, such as the Greater Pearl, Yangtze, and Huang-Huai-Hai regions, due to the widespread implementation of vegetation restoration programs which converted retired cropland to forest and grassland^10,14^. We found that the riverine F_CO2_ decreased as a function of forest cover, with an average decrease of 17.4 mmol m^−2^ d^−1^ for a 1% increase in forest cover (Supplementary Fig. 6.3). This is probably because the dissolved organic matter originated from forested watersheds with less anthropogenic modifications is less labile and accessible to the microbial community than that from agricultural watersheds^38,39^. In addition, the increased evapotranspiration after reforestation may have also decreased transport of labile organic matter and soil CO_2_ to the stream system. Furthermore, the stream water *p*CO_2_ was found to be positively related to fractional cropland cover across regions (Supplementary Fig. 6.4). Agricultural activities, such as ploughing and use of fertilizers, typically induce strong soil erosion and export of terrestrial organic carbon into the aquatic ecosystems. Meanwhile, agricultural land use in China is generally associated with high population densities and discharge of largely untreated wastewater^40^, which together cause elevated nutrient deliveries to rivers. These disturbances can enhance mineralization of carbon and efficient flushing of soil CO_2_ into rivers, sustaining their high *p*CO_2_ levels^14,30,32^. From another perspective, the positive response of *p*CO_2_ to cropland cover validates our hypothesis that conversion of cropland to forest will reduce the dissolved CO_2_ concentrations and thus the flux. This demonstrates that effective land use management can not only increase ecosystem carbon sequestration but also reduce CO_2_ emissions from streams and rivers.

The aquatic ecosystems in eastern China, including Greater Pearl, Yangtze, Huang-Huai-Hai, and NE China, have experienced widespread pollution and eutrophication caused by high nutrient loading from agriculture and urbanization since the early 1980s (refs. ^40,41^). Recent in situ measurements indicate that lakes typically function as small CO_2_ sources and even sinks owing to high primary productivity from algal blooms^42,43^. We therefore propose that intensive eutrophication was another potential driver for the declining CO_2_ evasion rates between the 1980s and 2010s, especially for standing waters that are particularly vulnerable to eutrophication. Overall, our analysis shows that the reduced riverine F_CO2_ in eastern China, largely driven by human disturbances (Fig. 4a), accounted for 58–97% of the decrease in riverine CO_2_ efflux. The F_CO2_ changes in lakes and reservoirs for these four regions (Figs. 4b and 4c) explained on average 82% of the variability in CO_2_ efflux changes (Supplementary Table 6.5). We hypothesize that the increasing use of fertilizers and urban sewage discharge may further inhibit CO_2_ emissions from inland waters in China. Hence, future work that considers these human impacts and accurately determines the resulting CO_2_ efflux changes is needed.

In comparison, damming and water withdrawals were important controls on the decreasing riverine CO_2_ efflux in arid NW China, a region with the largest temporal increase in reservoir surface area (277%, Fig. 4c). We propose that there are two mechanisms governing the declining riverine CO_2_ fluxes in NW China. First, dam construction and excessive water withdrawals have reduced the areal extent of gas exchange in natural rivers. Second, the reduced flow velocities after damming have led to a decrease in near-surface turbulence, thereby reducing the gas transfer velocity and subsequently F_CO2_ (refs. ^1,6,44^). The decrease in river water surface area in this region explained 74% of the variability in CO_2_ efflux changes. Similar human-induced disturbances can partially explain the decrease in riverine CO_2_ efflux (77%) in the Huang-Huai-Hai region, which is also characterized by a dry climate. The river surface area in this region decreased by 17% while the reservoir water surface increased by one-third between the 1980s and 2010s (Supplementary Table 4.11). Hence, we conclude that the observed decrease in F_CO2_ was the primary driver for the decrease in CO_2_ efflux in the wet regions (i.e., Greater Pearl, Yangtze, and NE China), while both F_CO2_ and water surface area changes were equally important in determining the decrease in CO_2_ efflux in the dry regions (i.e., Huang-Huai-Hai and NW China).

Unlike the rest of China, the Tibetan Plateau did not undergo any substantial anthropogenic perturbation between the 1980s and 2010. Here we reason that climate change was the primary factor responsible for the observed increase in CO_2_ emissions. First, accelerated melting of glaciers, snow, and permafrost driven by rising temperature coupled with increasing precipitation over the Tibetan Plateau has greatly expanded the surface area of its streams/rivers and lakes (Figs. 4a and 4b)^12,20^. Second, the climate change-driven hydrological alterations have caused higher flushing and transport of soil organic carbon and wetland/riparian CO_2_ that would otherwise be permanently stored in upland catchments or degassed into the atmosphere directly from terrestrial landscapes^45^. Consequently, the Tibetan Plateau was the only region where F_CO2_ increased in headwater streams (Supplementary Table 6.1), although the regional mean F_CO2_ has consistently declined in all six regions (Fig. 4a). The observed variations in F_CO2_ were responsible for 83–98% of the CO_2_ efflux increase from the Tibetan Plateau aquatic ecosystems (Supplementary Tables 6.4 and 6.5). This climate change-driven F_CO2_ increase may have also contributed to the increasing CO_2_ emissions from lakes and reservoirs in the NW China region, which depend heavily on glacier runoff (Figs. 4b and 4c). We suggest that degradation of ancient carbon released from melting glaciers and permafrost can constitute an important carbon source for the atmosphere^46,47^.

### Implications for China’s and global carbon budgets

The emitted CO_2_ is originated largely from terrestrial ecosystem respiration that enters the aquatic systems as dissolved soil/wetland CO_2_ or from mineralization of terrestrially derived organic matter within the aquatic domain that transforms organic carbon to dissolved CO_2_ (refs. ^2,7^). Therefore, the evasion of CO_2_ from inland waters should be accounted for when quantifying the carbon sink strength of terrestrial ecosystems. Based on literature values, we assume that respiratory soil CO_2_ contributed 90% of the degassed CO_2_ from headwater streams and 20% of the degassed CO_2_ from higher-order streams and from lakes and reservoirs^27,31,32^. This suggests that soil respiration contributed 77 and 56 Tg C yr^−1^ to the CO_2_ evasion flux from Chinese inland waters in the 1980s and 2010s, respectively. By subtraction, the remaining fluxes are from other sources (primarily aquatic mineralization of terrestrial organic matter) not counted in the estimation of terrestrial carbon sink using biomass accumulation techniques (see Methods for further details).

A recent study of carbon balance in China’s terrestrial ecosystems reports an average carbon sink of 177 ± 73 Tg C yr^−1^ in the 1980s based on biomass accumulation techniques^48^. Our efflux estimates could reduce the magnitude of the terrestrial carbon sink within China for the 1980s by 24–59% (see Methods and Supplementary Section 9). As a result of implementation of nationwide ecological restoration programs since the early 1980s, the terrestrial ecosystems across China have been greatly restored^15^. Updated estimates show that China’s terrestrial carbon sink has increased to 201–250 Tg C yr^−1^ in the 2000s (refs. ^10,49^) (Supplementary Section 9). If we use this range as the lower limit estimate for the period 2010s, accounting for the simultaneous CO_2_ evasion from Chinese inland waters suggests that the overall carbon sink capability of China’s terrestrial ecosystems could be offset by 17–21%. Because CO_2_ loss through inland water evasion is not yet incorporated into current carbon budgeting of China’s terrestrial landscape, we conclude that inland water CO_2_ emissions can substantially offset China’s terrestrial carbon sink.

The large CO_2_ effluxes from inland waters are also significant in the context of CO_2_ emissions from fossil fuel combustion of China, currently the largest emitter in the world^50^. The degassed CO_2_ from Chinese inland waters correspond to 25% and 4% of China’s carbon emissions from fossil fuel combustion in the 1980s and 2010s, respectively (Supplementary Section 9). The decreased percentage is mostly because of the surging emissions from fossil fuel combustion driven by rapid economic growth, but such emissions peaked in 2013 and started to decline since then^50^. Hence, the CO_2_ evasion from Chinese inland waters remains a non-negligible carbon source at the landscape scale. Including this evasion pathway in China’s carbon budget is crucial to better understand its natural carbon cycling and associated feedbacks on climate warming. Our efflux estimate from Chinese inland waters represents 5–7% of the global estimate of Raymond et al.^7^ and would cause a ~0.1 Pg increase over this estimate. Considering that China’s diverse climatic and geomorphologic systems mimic global landscapes and comprise most of the global vegetation types^10^, we contend that excluding inland water CO_2_ evasion could produce significant errors in understanding the role of terrestrial ecosystems in the global carbon balance.

This study represents the first comprehensive approach to evaluating changes in aquatic CO_2_ emissions through time. We show that direct management of both terrestrial and aquatic systems has the potential to significantly impact the carbon emissions from inland waters. Only an accurate assessment of CO_2_ emission changes due to management practices, and their influence on water resources, will allow us to fully understand how to couple aquatic carbon loss to terrestrial ecosystems.

## Methods

### Data acquisition

To calculate the CO_2_ emissions from Chinese inland waters in the 1980s, we relied on estimated surface water *p*CO_2_ because direct evasion measurements are not available. We compiled hydrologic gauge-based water chemistry records at 1709 locations over a period of 26 years (1960−1985). These records were retrieved from hydrological yearbooks published yearly by the Ministry of Water Resources of China. The water chemistry parameters include pH, water temperature, and alkalinity. We acquired 138,687 paired measurements, of which 86% were measured during the period 1976−1985. To avoid the directional bias of *p*CO_2_ calculation, we generally selected the locations with a minimum of 25 measurements for *p*CO_2_ estimation^32^. Due to paucity of hydrologic gauges on the Tibetan Plateau, we included additional water chemistry data in 107 Tibetan Plateau lakes to maintain an adequate spatial representation of locations across China (Supplementary Section 2). In total, we retained 126,713 measurements at 1508 locations for this analysis. For CO_2_ evasion in the 2010s, we collected dissolved CO_2_ concentration and/or CO_2_ evasion data at 1064 sampling locations across China. When CO_2_ evasion rates were not available from the literature (35% of the dataset), we calculated the site-specific CO_2_ evasion flux using dissolved CO_2_ concentration and gas transfer velocities (Supplementary Sections 3 and 5). We retrieved hydrological data (e.g., flow discharge, flow velocity, stream width, and channel slope) at the 1709 hydrologic gauges in the 1980s and 2010s for estimating the gas transfer velocity of streams and rivers. Ancillary data, including climate (precipitation, temperature, and wind speed) and land cover across China, were also collected for regression analysis (Supplementary Sections 2 and 3).

### Remote sensing and GIS analysis

We used a total of 507 satellite images (mainly Landsat 5 and 7 during the period 2005−2008) to delineate the inland waters across China. The inland waters were further divided into three categories: rivers (including artificial ditches), natural lakes, and artificial reservoirs with assistance of a script developed in C# and visual interpretation^51^. The lake and reservoir datasets have been published in our earlier research work^51–53^. The stream network was initially extracted from river centerlines and followed by manual restoration based on electronic maps and high-resolution satellite images, because most narrow small and medium-sized rivers are disconnected owing to shadows, clouds, and even vegetation. The network data represents the current real river status in China (see Supplementary Fig. 4.2). We assigned a numeric stream order to each river segment according to Strahler’s ordering system^54^. It is unreliable to use satellite images or DEM models to derive the stream width changes due to large water level fluctuations. Alternatively, we used the stream width measured at the 1709 hydrologic gauges to calculate the averaged stream width in the dry and wet seasons. The total water surface area of streams and rivers for each Strahler order was calculated based on total stream length and average stream width in each region. The stream network for the 1980s was obtained by backward updating the stream network dataset with the assistance of the aforementioned script and historical satellite images. If a river segment in the 1980s is longer than that in the 2010s, the river was flagged and then extended manually. If the river segment is an artificial river or ditch built after the 1980s, the river segment was deleted accordingly.

We realized that the satellite images captured in the 1980s showed low spatial resolution and the delineation results of lakes and reservoirs were characterized by high uncertainties, especially for small water bodies. To estimate the surface area of these lentic water bodies, we used national inventories^55–57^, which provide detailed information on abundance, surface area, and location of major lakes and reservoirs (see Supplementary Section 4) to overcome the uncertainty. For small lakes (<1 km^2^) which are undetectable from satellite images back to the 1980s, we assumed their abundance and size remained largely unchanged and used the estimates for the 2010s to estimate that in the 1980s. This may be slightly conservative as small lakes are particularly prone to rapid changes because of strong human activities^58^. However, because small lakes accounted for only 8.3% of the total surface area of lakes in the 2010s, this assumption would not greatly bias the total surface area of lakes in the 1980s. For small reservoirs in the 1980s, we estimated their surface area by using the number of small reservoirs available in the national inventories and averaged surface area of small reservoirs in the 2010s with the assumption that their spatial distribution across regions is similar to that in the 2010s (Supplementary Section 4).

### *p*CO_2_ calculation and calibration

Before we calculated the *p*CO_2_, we evaluated the data quality of the water chemistry records. As an example, we compared the pH and alkalinity measured on the Yangtze River with that measured under the United Nations Global Environment Monitoring System (GEMS)/Water Program (available at http://www.unep.org/gemswater). For the datasets from the two sources, the pH differed by <1.8% and the alkalinity by 7.6–13.9%, suggesting that the collected water chemistry data are reliable for *p*CO_2_ calculation (Supplementary Section 2). Furthermore, the calculated *p*CO_2_ is prone to large overestimation in low pH and low alkalinity conditions^59^. We corrected the *p*CO_2_ calculation errors caused by biased pH and organic alkalinity using the approach presented in Liu et al.^60^. All measurements with the alkalinity less than 1000 µmol L^−1^ were calibrated (Supplementary Section 2). We then calculated the *p*CO_2_ from pH, water temperature, and alkalinity using CO2calc program^61^. We further tested the reliability of this approach by comparing the calculated *p*CO_2_ results with direct *p*CO_2_ measurements using the headspace equilibrium technique. Our recent measurements (*n* = 329) in the Huang-Huai-Hai, NE China, and Tibetan Plateau regions showed that *p*CO_2_ predicted from water chemistry records agreed well with direct measurements (Supplementary Fig. 2.3). The calculated *p*CO_2_ slightly overestimated the actual *p*CO_2_ by 9%.

### Gas transfer velocity (*k*)

For streams and rivers where direct CO_2_ evasion measurements were not available, we computed their areal CO_2_ evasion from the gas transfer velocity (*k*) using two recently developed approaches (Supplementary Section 3). Both approaches estimate *k*_*600*_, the gas transfer velocity normalized to a Schmidt number of 600. The Raymond et al. (hereafter ‘Raymond’) approach^62^ relies on a general relationship to compute the *k*_*600*_ value from hydraulic parameters for all streams and rivers. The Ulseth et al. (hereafter ‘Ulseth’) approach^63^ uses two different scaling relationships between turbulence-induced energy dissipation rate and *k*_*600*_ to distinguish high-energy streams from low-energy streams. We report the effluxes computed from the Raymond approach and use the estimates from the Ulseth approach for comparison. The *k*_*600*_ values were highest in the headwater streams (range: 8.3–432 cm h^−1^ and average: 43 cm h^−1^) owing to the elevated turbulent energy driven by steep topography and exhibited a decreasing downstream trend.

For lakes and reservoirs, we estimated their gas transfer velocity by relating it to wind measured at 10 m height. Similarly, we used two empirical approaches^64,65^ for the *k*_*600*_ estimation, depending on wind speed (Supplementary Section 3). The breakpoint of wind speed was set at 3.7 m s^−1^. We classified lakes and reservoirs into three size classes based on surface area because their gas transfer velocities are highly size dependent^66^. The two approaches provided national average *k*_*600*_ values of 2.2 and 3.6 cm h^−1^, which are much lower than the estimates for streams and rivers but are comparable to recent global and regional studies^5,33^.

### CO_2_ efflux calculation

For the sampling locations without direct measurement of CO_2_ evasion, we calculated the areal CO_2_ efflux (F_CO2_, in mmol m^−2^ d^−1^) across the water–air interface using the following equation

1 $${\mathrm{F}}_{{\mathrm{CO2}}} = k \times k_{\mathrm{H}} \times \Delta p{\mathrm{CO}}_2$$

where *k* is the gas transfer velocity (cm h^−1^), *k*_*H*_ is Henry’s constant for CO_2_ corrected for temperature and pressure (mol L^−1^ atm^−1^) and Δ*p*CO_2_ is the water–air gas concentration gradient. Whereas a positive gradient corresponds to CO_2_ emission from the water to the atmosphere, a negative value indicates a carbon invasion into the water.

To better understand the spatial and seasonal variability in CO_2_ emissions and to determine the annual flux more accurately, we split China into six regions based on hydrologic, geomorphologic, and climatic differences, including Greater Pearl, Yangtze, Huang-Huai-Hai, northeast China (hereafter denoted as NE China), northwest China (hereafter denoted as NW China), and Tibetan Plateau (Supplementary Section 5). We then calculated the average F_CO2_ in the dry and wet seasons for each region. Furthermore, surface area of inland waters, especially for streams and rivers, exhibits significant seasonal variability as a result of the seasonality in precipitation and runoff^67^. We estimated the surface area of streams and rivers in the dry and wet seasons of the 1980s and 2010s. But for lakes and reservoirs, we only estimated their annual surface area in the 1980s and 2010s because they are subject to strong human impacts (e.g., dam operation and construction and land reclamation from lakes). With the seasonal estimates of F_CO2_ and water surface area, we separately computed the seasonal evasion flux for each region.

Evasion of CO_2_ from streams and rivers present a high degree of spatial heterogeneity across stream networks^31,68^. We calculated the CO_2_ efflux per Strahler order by multiplying the stream surface area per Strahler order by the averaged F_CO2_ of each Strahler order within each of the six defined regions. The total efflux (F_CO2total_, in Tg C yr^−1^) from all streams and rivers across China was computed as

2 $${\mathrm{F}}_{{\mathrm{CO2total}}} = \mathop {\sum }\limits_{{\mathrm{region}}} \left[ {\mathop {\sum }\limits_{{\mathrm{SO}}} \left( {\mathop {\sum }\limits_{{\mathrm{season}}} {\bar{\mathrm{F}}}_{{\mathrm{CO2}}} \times {\mathrm{SA}} \times {\mathrm{N}} \times {\mathrm{12}} \div {\mathrm{10}}^{{\mathrm{15}}}} \right)} \right]$$

where SO is the Strahler orders within a given region, $\bar {\mathrm{F}}_{{\mathrm{CO2}}}$ is the mean F_CO2_ of discrete sampling location-based results for a given SO within a given region, SA is the stream surface area in the dry and wet seasons (m^2^), N is the number of days in the dry or wet season considering only the ice-free period, 12 is the molar mass of carbon (12 g mol^−1^) and 10^15^ is the convertor from milligrams (mg) to teragrams (Tg).

Because the dissolved CO_2_ concentrations and gas transfer velocity of lake and reservoir waters are found to be highly dependent on surface area size^66,69,70^, we classified them into three size classes (<10, 10–50 and >50 km^2^) for efflux calculation. Similarly, we calculated the CO_2_ efflux per size class by multiplying the surface area of lakes or reservoirs per size class by the averaged F_CO2_ of each size class within each of the six regions. Finally, these flux results were aggregated by regions and seasons to calculate the total efflux from lakes or reservoirs across China, which can be expressed as

3 $${\mathrm{F}}_{{\mathrm{CO2total}}} = \mathop {\sum }\limits_{{\mathrm{region}}} \left[ {\mathop {\sum }\limits_{{\mathrm{class}}} \left( {\mathop {\sum }\limits_{{\mathrm{season}}} {\bar{\rm{F}}}_{{\mathrm{CO2}}} \times {\mathrm{SA}} \times {\mathrm{N}} \times {\mathrm{12}} \div {\mathrm{10}}^{{\mathrm{15}}}} \right)} \right]$$

where ${\bar {\mathrm{F}}}_{{\mathrm{CO2}}}$ is the mean F_CO2_ of discrete sampling results for a given water surface class within a given region, SA is the water surface area of the lakes or reservoirs within a given size class (m^2^) and N is the number of days in the dry or wet season considering only the ice-free period.

### Estimation of lateral export of soil CO_2_

Although it is well established that the degassed CO_2_ from inland waters is partly derived from soil CO_2_ produced by terrestrial respiration, it is still difficult to quantitatively determine this external contribution. To evaluate the implications of inland water CO_2_ evasion for terrestrial carbon sink by ecosystems, we estimated the contribution of terrestrial respiration to total efflux from Chinese inland waters. We assumed that soil respiration contributed 90% of the degassed CO_2_ from headwater streams and 20% of the degassed CO_2_ from higher-order streams and from lakes and reservoirs^27,31,32^. We then estimated that soil respiration in China contributed 77 and 56 Tg C yr^−1^ to the CO_2_ evasion flux from Chinese inland waters in the 1980s and 2010s, respectively. These fluxes have already been counted in the estimation of terrestrial carbon sink based on biomass accumulation techniques. By subtraction, the remaining fluxes (61 and 42 Tg C yr^−1^ in the 1980s and 2010s, respectively) are from other sources (e.g., primarily aquatic mineralization of terrestrial organic matter but also groundwater-derived inorganic carbon inputs) not considered in the estimation of terrestrial carbon sink using biomass accumulation techniques. We assessed their implications for China’s carbon budget in the 1980s and 2010s (Supplementary Section 9).

### Statistical and error analyses

We performed all simple linear regression analysis, including the determination of 95% confidence intervals, with Origin 9.1 software. Standard deviations of F_CO2_ from streams/rivers, lakes and reservoirs were calculated using Origin 9.1 software. We evaluated bivariate comparisons of continuous data measurements using analysis of variance (ANOVA) tests.

An error analysis on the computation and upscaling of CO_2_ evasion flux was performed by propagated error analysis of the surface water *p*CO_2_ measurement, the gas transfer velocity estimates, and the estimates of inland water surface area to scale the areal effluxes. We distinguished streams and rivers from lakes and reservoirs because of the great differences in the physicochemical characteristics between flowing and stagnant waters. We thus calculated their respective CO_2_ effluxes and tested the errors of these efflux calculations using a Monte Carlo simulation with 10,000 iterations (Supplementary Section 8). For lakes and reservoirs, we performed the Monte Carlo uncertainty analysis to estimate the error for each of the three size classes. Finally, we calculated the propagated error of the total CO_2_ efflux from inland waters across China by treating the errors on the two primary inland water types (streams/rivers and lakes/reservoirs) or on the six regions as being statistically independent. We used the 5th and 95th percentiles as confidence intervals. All analyses were performed using R (R version 3.6.0, R Core Team^71^).

## Supplementary information

Supplementary Information

## Data Availability

A summary of datasets used in this study is available in the Supplementary Section 10. The full dataset, including water chemistry records in the 1980s, major lakes and reservoirs across China in the 1980s and surface water *p*CO_2_ and CO_2_ emission measurements in the 2010s, is available at HKU DataHub (10.25442/hku.13560452.v3).

## Code availability

The code developed in C# for distinguishing different inland water bodies and for delineating river networks is available at HKU DataHub (10.25442/hku.13560452.v3).
