# Supplementary material for: Substantial decrease in CO2 emissions from Chinese inland waters due to global change
Source: Nat Commun. 2021 Mar 19;12:1730. doi: 10.1038/s41467-021-21926-6 (PMC7979821; doi:10.1038/s41467-021-21926-6)
Supplement: Supplementary file 1 — Supplementary Information [file 41467_2021_21926_MOESM1_ESM.pdf]

Supplementary Information for:

Substantial decrease in CO_2 emissions from Chinese inland waters due to global change

Lishan Ran[1*], David E. Butman[2], Tom J. Battin[3], Xiankun Yang[4*], Mingyang Tian[4], Clément Duvert[5], Jens Hartmann[6], Naomi Geeraert[7], Shaoda Liu[8]

[1]Department of Geography, The University of Hong Kong, Pok Fu Lam Road, Hong Kong
[2]School of Environmental and Forest Sciences, University of Washington, Seattle, WA, USA
[3]Stream Biofilm and Ecosystem Research Laboratory, School of Architecture, Civil and Environmental Engineering, École Polytechnique Fédérale de Lausanne, Lausanne, Switzerland
[4]School of Geography and Remote Sensing, Guangzhou University, Guangzhou, China
[5]Research Institute for the Environment and Livelihoods, Charles Darwin University, Darwin, Northern Territory, Australia
[6]Institute for Geology, Center for Earth System Research and Sustainability (CEN), Universität Hamburg, Hamburg, Germany
[7]School of Biological Sciences, The University of Hong Kong, Pok Fu Lam Road, Hong Kong
[8]State Key Laboratory of Water Environment Simulation and Modelling, School of Environment, Beijing Normal University, Haidian District, Beijing, 100875, China
Correspondence: lsran@hku.hk (L.R.) or yangxk@gzhu.edu.cn (X.Y.)

Contents

Introduction
1. Division of the inland water system across China
2. Data collection and pCO_2 calculation
2.1 Water chemistry dataset in the 1980s and pCO_2 calculation
2.2 Dataset for evaluation of CO_2 emissions in the 2010s
2.3 Correcting pCO_2 calculation errors from biased pH and organic alkalinity
3. Estimation of gas transfer velocity
3.1 Gas transfer velocity in streams and rivers
3.2 Gas transfer velocity in lakes and reservoirs
4. Surface area of Chinese inland waters
4.1 Water surface area in the 1980s
4.1.1 Streams and rivers
4.1.2 Lakes and reservoirs
4.2 Water surface area in the 2010s
4.2.1 Streams and rivers
4.2.2 Lakes and reservoirs
4.3 Comparison of water surface area between 1980s and 2010s
5. CO_2 efflux calculation
5.1 Areal CO_2 efflux
5.2 Total CO_2 efflux
5.2.1 CO_2 efflux from streams and rivers
5.2.2 CO_2 efflux from lakes and reservoirs
6. Spatial and temporal variations of CO_2 emissions
6.1 River pCO_2 change along the stream network
6.2 Overall CO_2 emission fluxes
6.3 River CO_2 efflux by Strahler order
6.4 Potential drivers for the decreasing CO_2 evasion
6.4.1 Human factors
6.4.2 Natural factors
6.4.3 Attribution analysis of human and natural factors
7. Robustness test of temporal changes in F_{CO2} between 1980s and 2010s
8. Uncertainties in CO_2 effluxes
9. Implications for assessing China's and global carbon budgets
10. Datasets used for estimating CO_2 emissions
References for supplementary material

Introduction

We estimated the flux of CO_2 emissions from Chinese inland waters, including streams, rivers, lakes and reservoirs, in two time periods. The first period is the 1980s, soon after the initiation of the reform and opening-up policy while before the rapid economic growth (and strong human disturbances) which is unprecedented in modern human history[1, 2]. The second period is the 2010s that reflects strong anthropogenic perturbations across China, such as damming and intensive land use changes. For the CO_2 efflux in the 1980s, we used water chemistry dataset primarily collected from the Hydrological Yearbooks published yearly by the Ministry of Water Resources of China to calculate the CO_2 partial pressure (pCO_2). The areal CO_2 efflux (CO_2 emission per unit water surface area; F_{CO2}) was estimated based on pCO_2 and gas transfer velocity across the water-air interface. For the areal CO_2 efflux in the 2010s, we used floating chamber-based CO_2 emission measurements and, when direct evasion measurements were not available, water chemistry records from the literature to estimate CO_2 emissions from Chinese inland waters. Surface area of different types of water bodies was estimated based on delineation of satellite images along with national river, lake and reservoir survey datasets. It is important to note that we considered the seasonal variations in water surface area and areal CO_2 emissions in both time periods. We therefore feel confident that the obtained flux estimates are reliable and convincing. Below are the supplementary methods (Sections 1–5) and discussion (Sections 6–9). Information on the datasets used in this analysis is provided in Section 10.

1. Division of the inland water system across China

China has a huge land surface area of ~9.6 million km^2 and spans a broad range of latitude (from 18°N to 53.5°N) and climatic conditions (from tropical, temperate to dry and cold climates) to which diverse ecosystem types have evolved. Its elevation varies from less than 30 m in the Eastern China Plain to more than 4000 m on the Tibetan Plateau, displaying pronounced variability in hydrology and geomorphology. In order to account for spatial heterogeneity in hydrology, climate and geomorphology across the country, we split China into six regions (Figure 1.1) based largely on hydrologic units but also consider the differences in climate and geomorphology as best as possible. This allowed us to maintain the complete and contiguous hydrological units for upscaling. These six regions are Greater Pearl, Yangtze, Huang-Huai-Hai, northeast China (hereafter referred to as NE China), northwest China (hereafter referred to as NW China), and Tibetan Plateau (Figure 1.1). The Huang-Huai-Hai region includes three large rivers, namely the Huang River (Yellow River), Huai River and the Hai River. The first two regions are located in southern China and are predominantly wet and warm, whereas the second three regions are located in northern China and are comparatively dry and cold. Together, the five regions exhibit largely low-to-medium elevations. In comparison, the Tibetan Plateau region is characterized by high elevations (>3000 m a.s.l.) and shows an extremely cold highland climate. Table 1.1 shows the basic climatic and hydrologic characteristics of the six regions. Based on the hydrological regime of each region controlled by climate, the wet and dry seasons in the six regions were also shown in Table 1.1. Furthermore, water bodies in northern China, including the NE China and NW China regions, and in Tibetan Plateau will freeze in winter. Duration of the freezing period ranges from 80 to 120 days[3, 4] (Table 1.1).

Both human disturbances and climate change have greatly affected the inland water system across China. For example, excessive water withdrawal along with dam impoundment (thus elevated evaporation) has caused river flow dry-up and lake shrinkage in eastern China[5, 6, 7], and

glacier recession and permafrost thawing due to climate change have resulted in diverse lake dynamics on the Tibetan Plateau with some lakes shrunk or even disappeared in the western part of the plateau while new lakes emerged mainly on the eastern plateau over the past decades [8, 9, 10, 11]. As outlined below, within each of the six defined regions we estimated the surface area of rivers, lakes and reservoirs, surface water $p\text{CO}_2$ and CO_2 effluxes from rivers, lakes and reservoirs in the 1980s and 2010s. The efflux results for these regions are then summed up to calculate the total CO_2 emissions from Chinese inland waters in the two time periods.

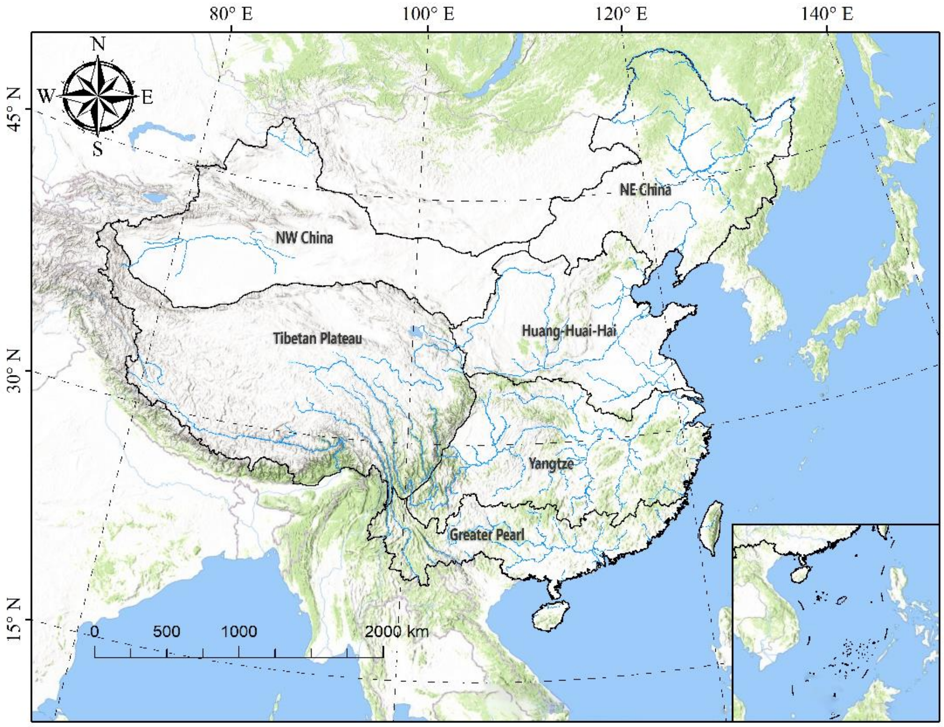

Figure 1.1 Division of the inland water system across China. The names correspond to the regions discussed in the text.

Table 1.1 Climatic and hydrologic characteristics of the six defined regions.

Region	Climate type	MAT (°C)	MAP (mm)	Wet season	Dry season	Freezing period (days)
Greater Pearl	Subtropical monsoon	20	1470	May–September	October–April	0
Yangtze	Subtropical monsoon	17	1100	May–September	October–April	0
Huang-Huai-Hai	Temperate monsoon	10	480	July–October	November–June	0
NE China	Temperate monsoon	5.5	550	July–September	October–June	90
NW China	Temperate continental	11	120	July–September	October–June	80
Tibetan Plateau	Highland climate	5	430	June–September	October–May	120

MAT: mean annual temperature; MAP: mean annual precipitation.

2. Data collection and pCO_2 calculation

2.1 Water chemistry dataset in the 1980s and data reliability

We estimated dissolved CO_2 concentrations in Chinese inland waters from water chemistry records retrieved from Hydrological Yearbooks published by the Ministry of Water Resources of China. The chemistry records for each sampling event include alkalinity, major ions, dissolved silica and concurrent environmental and hydrologic variables, such as pH, water temperature, flow discharge, flow velocity, stream slope, channel width and depth. The measurements were taken from 1960 to 1985 and the sampling frequency varied from 1 to 29 times per month depending on flow regimes. It is important to note that not all sites contained a complete and continuous record. While sampling at some stations was halted during the period 1966–1975, approximately 60% of the gauges had data for more than 8 years, usually from 1976 to 1985. In order to calculate robust and reliable pCO_2, measurements with a pH lower than 6.5 (5.7% of the considered measurements) were first discarded from the analysis because the calculated pCO_2 is prone to high errors owing to high noncarbonated alkalinity from organic acid anions[12, 13, 14, 15] and biased pH measurements due to low ionic strength of the waters. A total of 1,709 hydrologic gauges with 138,687 water chemistry measurements for pH, water temperature and alkalinity were finally compiled for this analysis. An important issue regarding historical datasets is their reliability and uncertainty. Yet, no assessment reports on quality assurance and quality control are available in the Hydrological Yearbooks. Our earlier work[16] on the Yangtze River evaluated the pH and alkalinity at Wuhan Hydrologic Gauge retrieved from the Hydrological Yearbooks with that from the United Nations GEMS/Water Program (Table 2.1). Both the pH and alkalinity data from both sources agreed well with one another (i.e., <1.8% difference for pH and 7.6–13.9% difference for alkalinity)[16]. Thus, the water chemistry data collected from the Hydrological Yearbooks are reliable for pCO_2 calculation. Previous comparisons of major ions and alkalinity in the Yangtze, Yellow and Pearl rivers measured by the two agencies have also validated the high data reliability of the Hydrological Yearbooks[17, 18, 19]. We thus concluded that the water chemistry data are reliable with high confidence.

Table 2.1 Comparison of alkalinity ($\mu eq\ L^{-1}$) and pH at Wuhan Hydrologic Gauge (on the Yangtze River) between the GEMS/Water Program results and the Hydrological Yearbooks[16]. Expressed as mean±standard deviation (s.d.).

Item	1980	1981	1982	1983	1984	1985
GEMS/Water Program						
Alkalinity	2050±286	2004±188	2000±232	1838±252	2200±247	1992±219
pH	7.83±0.16	7.73±0.24	8.04±0.09	8.06±0.05	8.00±0.09	7.88±0.06
Hydrological Yearbooks						
Alkalinity	2310±314	2187±236	2274±268	2033±304	2383±277	2306±238
pH	7.93±0.09	7.87±09	8.01±0.09	7.94±0.08	7.93±0.10	7.98±0.08

To avoid the directional bias of pCO_2 calculation caused by spurious pH measurements[15], we selected a minimum of 25 measurements for pCO_2 estimation as suggested by Butman and Raymond (2011)[20]. However, to maintain an adequate spatial coverage of hydrologic gauges in regions with a paucity of data as much as possible, we retained the gauges (~0.6% of the total) with ≥3 measurements in the dry and wet seasons, mainly in the Tibetan Plateau. Out of the 1,709 hydrologic gauges, 1,401 gauges met this requirement and were identified for this analysis (Figure 2.1). Moreover, because of the paucity of hydrologic gauges on the Tibetan Plateau, we

collected additional water chemistry data measured in 107 lakes in the 1980s from Zheng (1989)[21]. In total, we assembled 126,713 paired measurements at 1,508 locations, including 1,316 sites in streams/rivers, 122 sites in lakes and 70 sites in reservoirs. Frequency distribution of the sampling dates was shown in Figure 2.2. Surface water pCO_2 was computed from pH, water temperature, and alkalinity data using CO2calc program[22]. For discussion on the calibration of pH and alkalinity, see Section 2.3 below. Carbonic acid disassociation constants (i.e., pK1 and pK2) from Millero (1979)[23] were used for the computations. However, this conventional method would produce unrealized extremes because of biased pH measurements and significant non-carbonate alkalinity in low ionic strength waters[13, 24]. We thus reported median values per hydrologic gauge instead of means to avoid the impact of erroneous extremes.

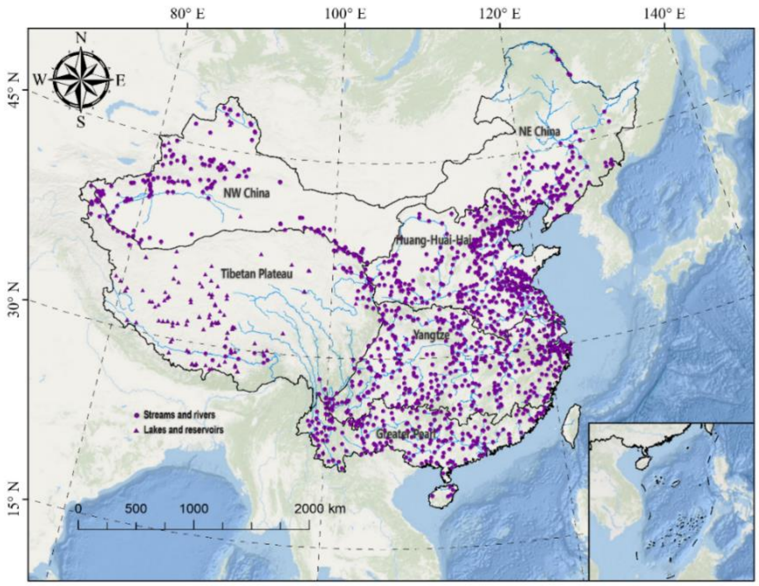

Figure 2.1 Spatial distribution of hydrologic gauge-based sampling locations across China.

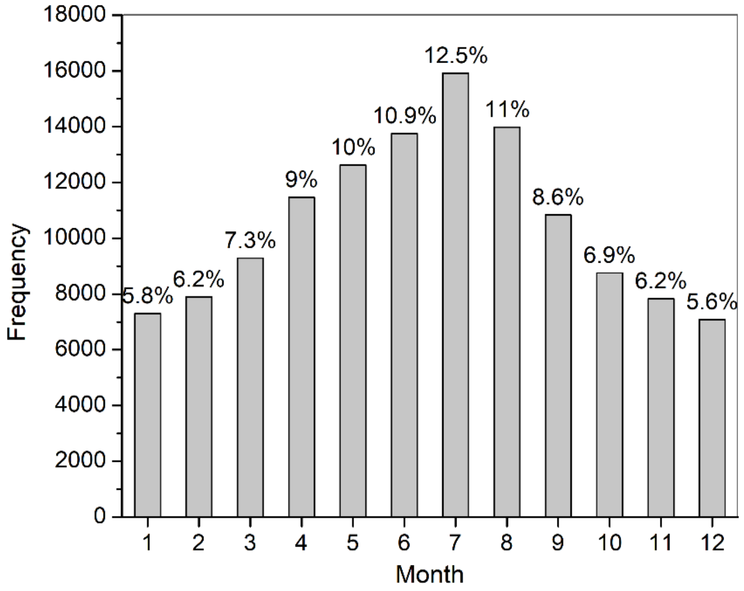

Figure 2.2 Frequency distribution of the sampling dates. The % above the bar indicates the percentage of measurements in a given month accounting for all measurements (126,713).

In order to evaluate the accuracy of the computed pCO_2, we compared the predicted pCO_2 results against *in situ* surface water pCO_2 measurements obtained by the headspace equilibrium method[25, 26, 27]. In our recent field surveys in the Huang-Huai-Hai[26], NE China (Ran, unpublished) and Tibetan Plateau[28], we measured the basic water quality parameters, such as pH, water temperature, alkalinity, salinity, dissolved oxygen and conductivity with a portable multi-parameter probe (Multi 3420, WTW GmbH, Germany). Meanwhile we measured the surface water pCO_2 using a non-dispersive infrared Licor 7000 or Licor 850 gas analyzer (LI-COR Environmental, NE, USA). In total, we collected 329 data pairs for predicting surface water pCO_2 from water quality parameters by using the CO2calc program[22]. The pH ranged from to 7.11 to 9.29, which covered 96% of our historical pH values. Our analysis shows that calculated pCO_2 results agreed well with the direct measurements (Figure 2.3), suggesting that for samples with pH > 7, pCO_2 can be reasonably estimated using pH and alkalinity according to carbonate equilibria. The calculated pCO_2 slightly overestimated the actual pCO_2 by 9%.

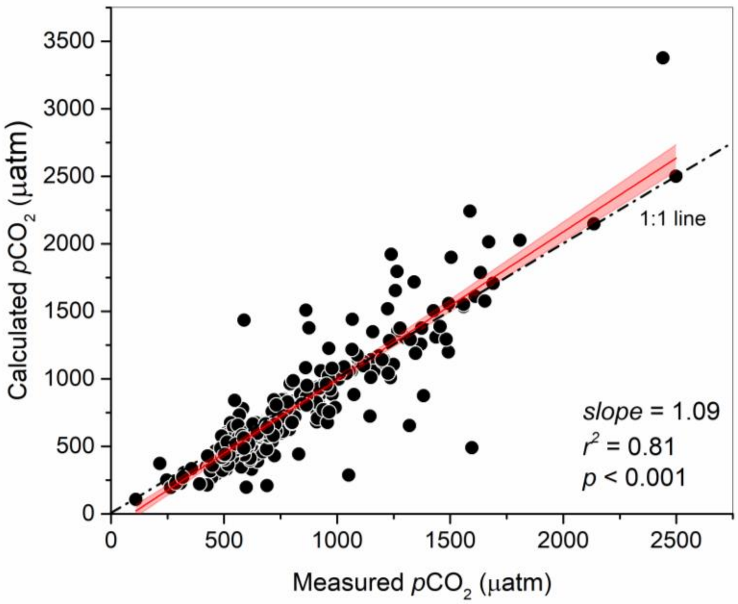

Figure 2.3 Comparison of calculated pCO_2 and measured pCO_2. The red line is the regression and the red band represents the 95% confidence interval (95%CI) of the regression model.

2.2 Dataset for evaluation of CO_2 emissions in the 2010s

To evaluate CO_2 emissions from Chinese inland waters in the 2010s, we conducted *in situ* CO_2 emission measurement using the commonly used floating chamber method in different climatic conditions, including Greater Pearl, Huang-Huai-Hai, NE China and Tibetan Plateau (Figure 2.4). In addition, the headspace equilibrium method was used to quantify the pCO_2 in water and ambient air. In total, we compiled a dataset of 198 sites scattered in river catchments with diverse climate, hydrology and geomorphology. In addition, we collected inland water pCO_2 and CO_2 emission results from the literature. Although our priority is to collect the measurements that were conducted in the 2010s (91 out of 113 peer-reviewed papers), we include 22 peer-reviewed papers that were published in the 2000s to increase the spatial representation of sites across China (see data information in Section 10). The raw data were either retrieved from tables or extracted from graphs using GetData Graph Digitizer (version 2.26, available at http://getdata-graph-digitizer.com/). For each paper, we compiled the following information: source of data,

location of sampling sites (longitude, latitude and altitude), climatic information (i.e., average annual precipitation and temperature), sampling date and/or frequency, Strahler order, pH, dissolved carbon, water temperature, pCO_2 and areal CO_2 evasion rate. Our final dataset consisted of 113 papers encompassing 1,064 sampling locations, of which 661 were conducted in streams and rivers, 204 in lakes and the remaining 199 in reservoirs. The sampling sites reported from the literature are widely distributed across China (Figure 2.4). All datasets along with the sampling date were compiled in an Excel file (see Section 10 for further details).

Of the literature-derived data, all CO_2 effluxes were expressed in units of mmol m^{-2} d^{-1} for flux calculation. If the measurements only reported surface water pCO_2 (~35% of the dataset), we estimated the air pCO_2 by considering CO_2 concentration change with altitude and assuming the air pCO_2 near sea surface at 400 µatm. We calculated their CO_2 effluxes by using the predicted gas transfer velocity. Fortunately, approximately 73% of the data retrieved from the literature reported CO_2 effluxes and only 27% of the datasets reported only surface water pCO_2.

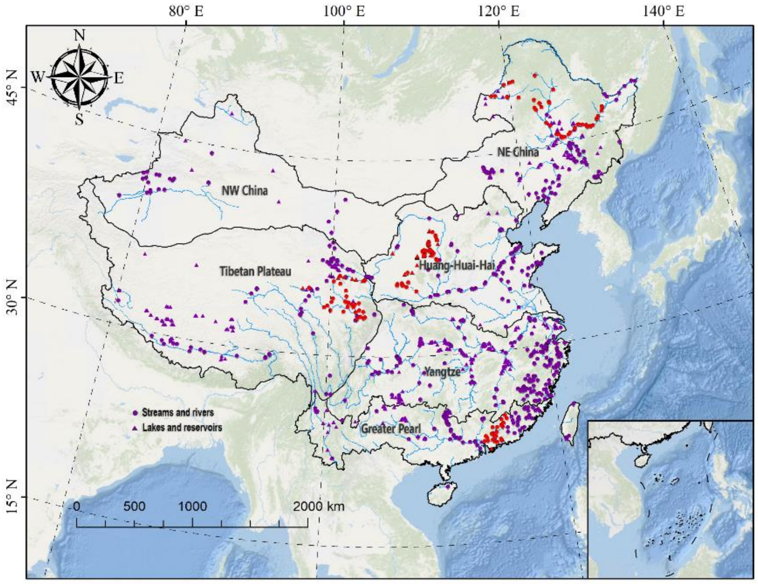

Figure 2.4 Spatial distribution of sampling locations used for estimating CO_2 emissions in the 2010s. The names correspond to the regions discussed in the text. Red circles (●) and triangles (▲) denote our own direct measurements of CO_2 evasion and surface water pCO_2 during the period 2015–2019 using floating chambers and headspace equilibrium technique, respectively.

2.3 Correcting pCO_2 calculation errors from biased pH and organic alkalinity

Contributions of non-carbonate species to total alkalinity can affect the calculation of pCO_2 from pH, alkalinity and water temperature[12, 29, 30]. Recent studies suggest that pCO_2 calculated from the carbonate equilibria is unreliable in organic-rich acidic waters because of high organic alkalinity[12, 13, 31] and biased pH measurements[32, 33]. The validity of estimating pCO_2 from pH, alkalinity and water temperature is most robust and reliable in freshwaters with circumneutral to basic pH and with the alkalinity exceeding 1000 µmol L^{-1}, whereas the pCO_2 would be greatly overestimated in low pH and low alkalinity conditions[12]. In addition to the removal of measurements with the pH lower than 6.5 as mentioned in Section 2.1, we further corrected the pH and organic alkalinity-related CO_2 calculation errors for measurements with alkalinity <1000 µmol L^{-1} following a roadmap model proposed by Liu et al (2020)[34]. Briefly, errors caused by

both organic alkalinity and pH measurement biases were corrected. pH measurement bias was estimated using a relationship between pH measurement error and ionic strength of the waters, where the ionic strength was estimated from its relationship with alkalinity. Organic alkalinity was estimated by applying a ratio to dissolved organic carbon concentration of the waters. Although dissolved organic carbon was not reported in the original datasets, it can be estimated using an assumed exponential relationship with total alkalinity of the waters. See detailed procedures recommended in Liu et al (2020)[34].

Overall, we corrected 7,359 measurements measured at 335 sampling locations (Figure 2.5), which account for 0.08–13.6% of the total across the six regions or 5.8% of the entire dataset for the 1980s. The spatial distribution of the low alkalinity waters can be explained by the lithology and land surface characteristics. The southeastern China and the northern part of the NW China regions are predominantly composed of silicates and have low alkalinity concentrations. For the corrected measurements, the frequency distribution of their pH values was presented in Figure 2.6. Approximately half of the measurements showed a pH in the range of 6.5–7.0.

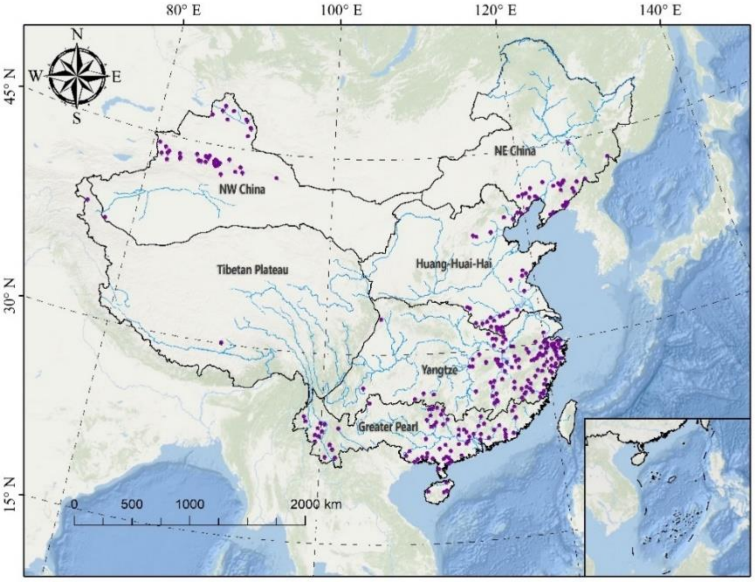

Figure 2.5 Location map of the 335 corrected gauge-based sampling locations.

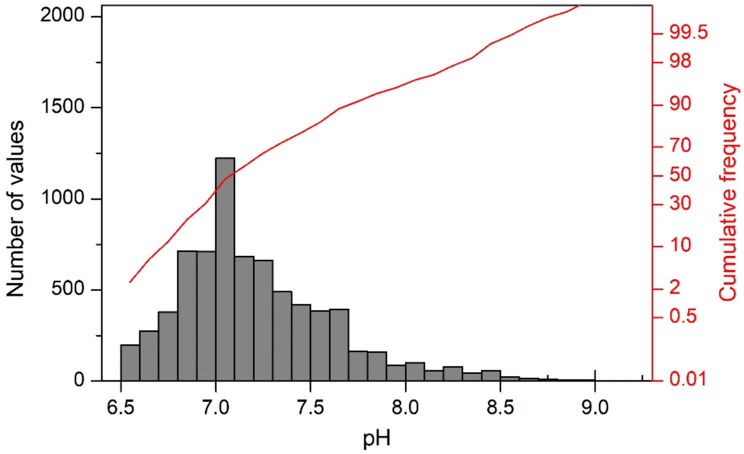

Figure 2.6 Frequency distribution of pH for the corrected measurements (n = 7359).

After the correction, the surface water pH increased on average by 0.16 units across the six regions, while the total alkalinity decreased by 17.6–29.4% (Table 2.2). Consequently, for the corrected measurements, the re-calculated pCO_2 decreased by about 50% in the Greater Pearl, Yangtze, NE China and NW China regions, and 42.6–45% in the remaining two regions (Table 2.2). The pCO_2 overestimation could be 300% or even higher when the total alkalinity was less than 500 μmol L^{-1} and the overestimation declined with increasing alkalinity (Figure 2.7a). A frequency distribution analysis shows that a large majority of the overestimations (94.5%) are below 200% which corresponds to a 66.7% reduction in pCO_2 after correction (Figure 2.7b). This large reduction is consistent with the need for caution raised by Abril et al (2015)[12] who suggested that calculated pCO_2 for low-alkalinity waters would be overestimated by 50–300% on average. Based on the corrected pH and alkalinity, we re-calculated the pCO_2. Considering that the proportion of the corrected pCO_2 is small (5.8% of the entire dataset), the impact of the correction on the overall efflux calculation is likely to be minor.

Table 2.2 Correction of pH and organic alkalinity-induced errors in the calculated pCO_2.

Region	No. of sites	No. of data records	Original pH range	pH after correction	Original mean alkalinity (μmol L^{-1})	Alkalinity change after correction (%)	Original mean pCO_2 (μatm)	pCO_2 change after correction (%)
Greater Pearl	92	2545	6.5–9.0	+0.157	687	-29	4803	-50.7
Yangtze	110	2647	6.5–7.7	+0.159	656	-28	3139	-50.2
Huang-Huai-Hai	29	690	6.5–9.2	+0.156	742	-17.6	3573	-42.6
NE China	30	706	6.5–8.9	+0.162	587	-29.4	2896	-51.5
NW China	43	765	6.6–9.1	+0.165	486	-26.3	888	-49
Tibetan Plateau	2	6	7.1–8.1	+0.161	507	-20	615	-45
Total	335	7359						

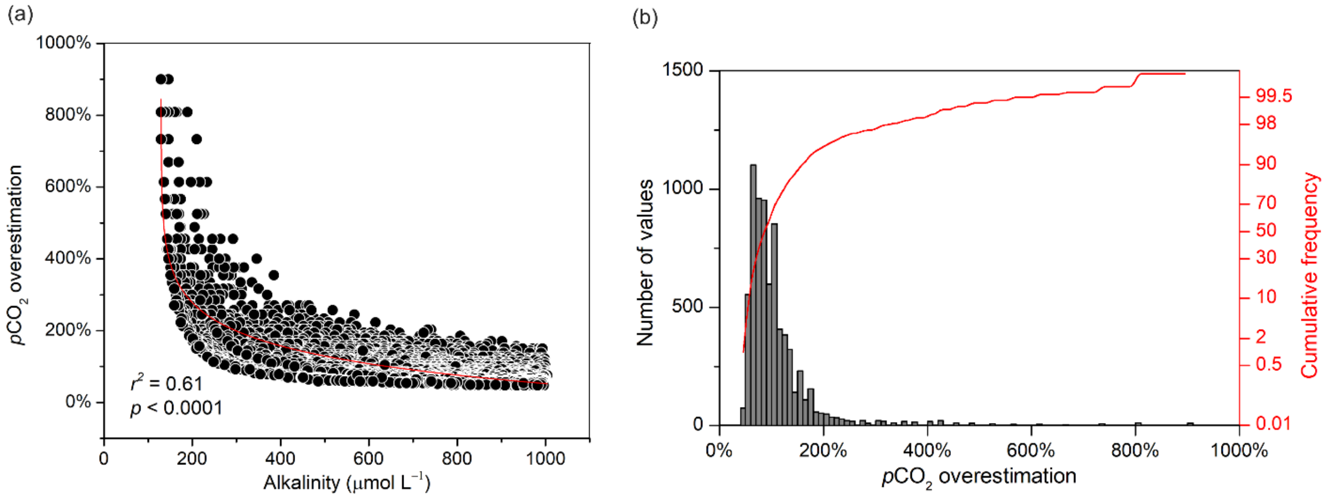

Figure 2.7 Significant overestimation of pCO_2 in low alkalinity waters (n = 7359). (a) decreasing overestimation of pCO_2 with increasing alkalinity, (b) frequency distribution of pCO_2 overestimation. Here the pCO_2 overestimation was calculated as 100 × (calculated pCO_2 – corrected pCO_2) / corrected pCO_2. Thus, a 100% overestimation corresponds to a 50% reduction in pCO_2 after correction and a 200% overestimation corresponds to a 66.7% reduction in pCO_2 after correction as shown in Table 2.2.

3. Estimation of gas transfer velocity

3.1 Gas transfer velocity in streams and rivers

We estimated the gas transfer velocity (k) for each sampling site across China. Hydraulic geometry parameters, including flow velocity (V), flow discharge (Q), stream width (W) and channel slope (S), were retrieved from Hydrological Yearbooks. Measurements of these parameters at each hydrologic gauge were usually daily performed and yearly compiled for research purpose. We calculated the average hydraulic geometry parameters (e.g., V, Q and W) in the wet and dry seasons for each sampling site.

Because direct measurements of k in Chinese inland waters in the 1980s are not available in literature, we employed empirical models to predict the k for each sampling site (hydrological gauge). Gas transfer across the water-air interface of streams and rivers is predominantly controlled by turbulence generated by water flow and bed roughness[35, 36, 37]. Raymond et al (2012)[36] proposed 7 models to predict k based on stream hydraulics and energy dissipation theories. Stream velocity and channel slope are the primary determinants for turbulence, and are thus used to construct the empirical relationship to estimate k. We used Model 5 in Raymond et al (2012)[36] (hereafter 'Raymond') to estimate the k in this study. In this model, the standardized k to a Schmidt number of 600 (hereafter referred to as k_{600}) can be predicted as a product of channel slope (S, m m^{-1}) and flow velocity (V, m s^{-1}):

$$k_{600} \text{ (m d}^{-1}) = 2841 \times VS + 2.02 \quad (1)$$

In our earlier study in the low-energy Wuding River catchment (drainage area: 30261 km^2) on the Loess Plateau (in the Huang-Huai-Hai region; Figure 1.1), we compared the predicted k_{600} using the Raymond approach with our calculated k_{600} results (Figure 3.1a). We deployed 170 freely drifting floating chambers to measure the CO_2 emission flux within the Wuding River catchment over three seasons and measured the pCO_2 in stream water and ambient air. With the CO_2 emission flux and concentration gradient of CO_2, we calculated the actual k_{600} by assigning the Schmidt number exponent a value of 0.5 (ref [38]). The comparison showed high consistency between the measured k_{600} and the modeled k_{600} (Figure 3.1a). The actual k_{600} calculated from floating chamber deployments was very close to the predicted k_{600}. For comparison, we constructed a model for predicting the k_{600} of the Wuding River catchment by using V and S as the proxies of near-surface turbulence (Figure 3.1b). Our model is consistent with the Raymond approach. We thus feel it appropriate to use the Raymond approach for estimating the k_{600} value.

To explore the applicability of Raymond approach in predicting k for streams in other regions with different climate and geomorphology, we further measured riverine CO_2 emissions in the subtropical Dongjiang River basin in subtropical southern China (in the Greater Pearl region; Figure 1.1). Following the same *in situ* monitoring method as used in the Wuding River catchment, freely drifting floating chambers were deployed to measure CO_2 emissions across the water-air interface. Like the arid-to-semiarid Wuding River catchment, the Raymond approach can predict the k_{600} with high confidence. Again, we constructed an empirical model to predict the k_{600} using V and S (Figure 3.2). Surprisingly, the established empirical relationship for the Dongjiang River basin shows a high degree of similarity to that for the Wuding River catchment (Figures 3.1 and 3.2). Considering the great difference between the two rivers in climate, hydrology, geomorphology and lithology, the high consistency between the measured k_{600} and

the modeled k_{600} suggests that the Raymond approach can be used to predict k for streams and rivers across China.

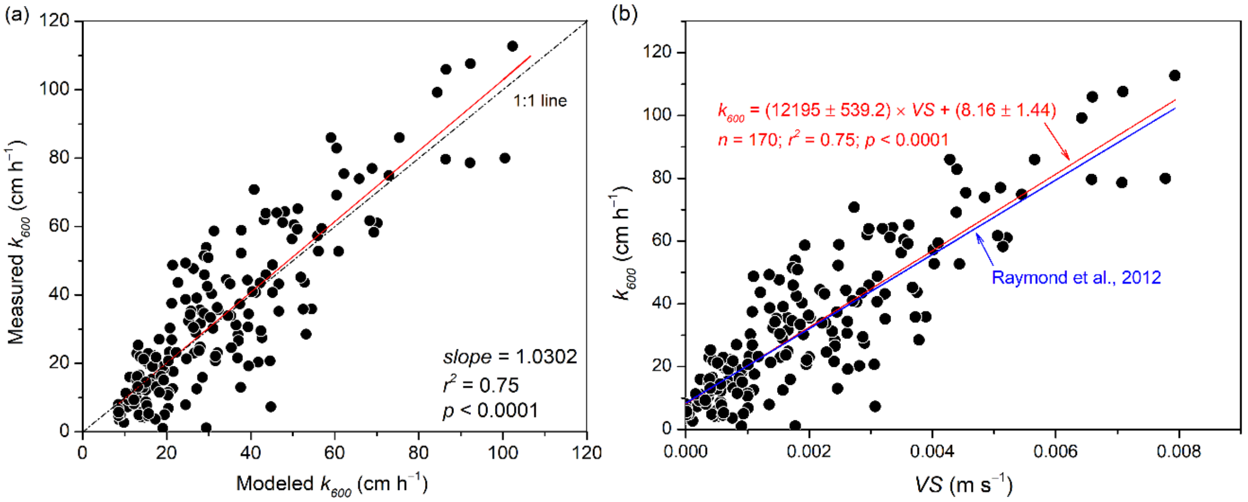

Figure 3.1 Prediction of k_{600}. (a) Relationship between measured k_{600} and predicted k_{600} by using the Raymond approach, (b) Revised relationship between k_{600} and hydraulic geometry parameters (flow velocity: V and channel slope: S) and its comparison with Raymond approach. Panel (a) was adapted from Ran et al (2017)[26].

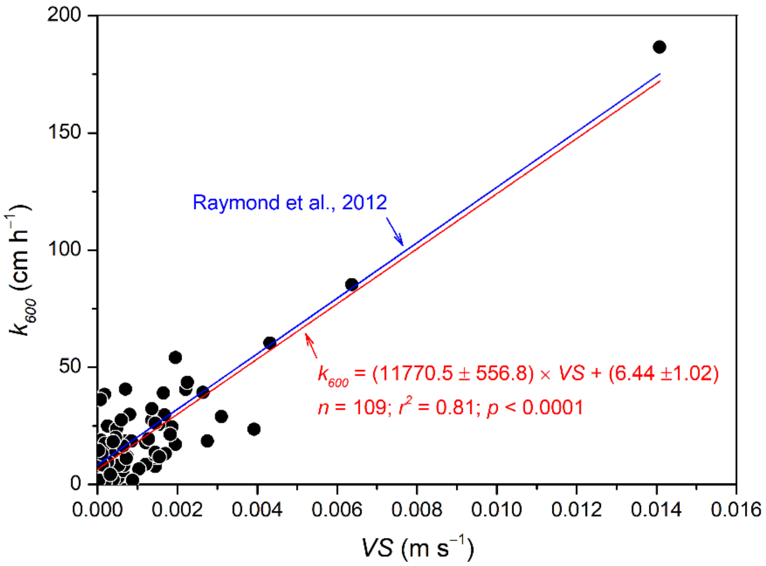

Figure 3.2 Predicting k_{600} in the Dongjiang River basin (in the Greater Pearl region) from VS.

However, we recognize that the scaling relationships are mostly based on streams with gentle channel gradients and low stream energy. These relationships may not be applicable to streams and rivers with high stream channel gradient, such as those in Tibet Plateau region. These streams and rivers are typically characterized by deeply incised channels, steep slope, turbulent flow and high stream energy. Owing to the strong near-surface turbulence generated from channel geomorphology and hydraulics, these rivers generally exhibit distinct air-water gas transfer regimes compared to low-energy rivers[39, 40]. A simple generalization of their gas transfer velocities using the established scaling relationships from low channel slope rivers would significantly underestimate the k value. A recent study reported that the k_{600} in 12 Swiss alpine

streams was unexpectedly high with an average of 464±780 m d^{-1}, which was approximately two orders of magnitude higher than those reported for low-energy rivers[41]. Therefore, it is important to develop a more suitable scaling relationship for these streams and rivers to reflect their unique geomorphological controls on gas transfer. Ulseth et al (2019)[41] used the energy dissipation rate as proxy for near-surface turbulence to scale and predict the k_{600} for streams and rivers with different channel energy levels. The empirical relationship between k_{600} (unit: m d^{-1}) and energy dissipation rate can be described as follows:

$$\ln(k_{600}) = 3.10 + 0.35 \times \ln(eD) \quad \text{when } eD < 0.02 \qquad (2)$$
$$\ln(k_{600}) = 6.43 + 1.18 \times \ln(eD) \quad \text{when } eD > 0.02 \qquad (3)$$

where eD is the channel energy dissipation rate (m^2 s^{-3}) and is the product of gravitational acceleration (g, m s^{-2}), channel slope (S, m m^{-1}) and flow velocity (V, m s^{-1}). As a measure of near-surface turbulence, an eD threshold of 0.02 m^2 s^{-3} indicates the shift of gas transfer velocities from mountainous high-energy streams ($eD > 0.02$ m^2 s^{-3}) to lowland low-energy streams ($eD < 0.02$ m^2 s^{-3}) along the stream eD continuum.

We calculated eD for 1316 stream and river sampling sites and further modeled their k_{600} using Equation 2 or 3 depending on the obtained eD. To evaluate the consistency of Raymond approach and the models developed by Ulseth et al (2019)[41] (hereafter 'Ulseth'), we compared the k_{600} values predicted by both methods. For low-energy streams (i.e., $eD < 0.02$ m^2 s^{-3}; the inset in Figures 3.3a and 3.4a), the modeled k_{600} values by the Raymond approach are very close to those estimated by the Ulseth approach for both the wet and dry seasons. However, for high-energy streams (i.e., $eD > 0.02$ m^2 s^{-3}; Figures 3.3b and 3.4b), there is a difference of 41–45% in k_{600} between the two methods as implied by the slope of the linear regression fit (0.5938 in the dry season and 0.549 in the wet season).

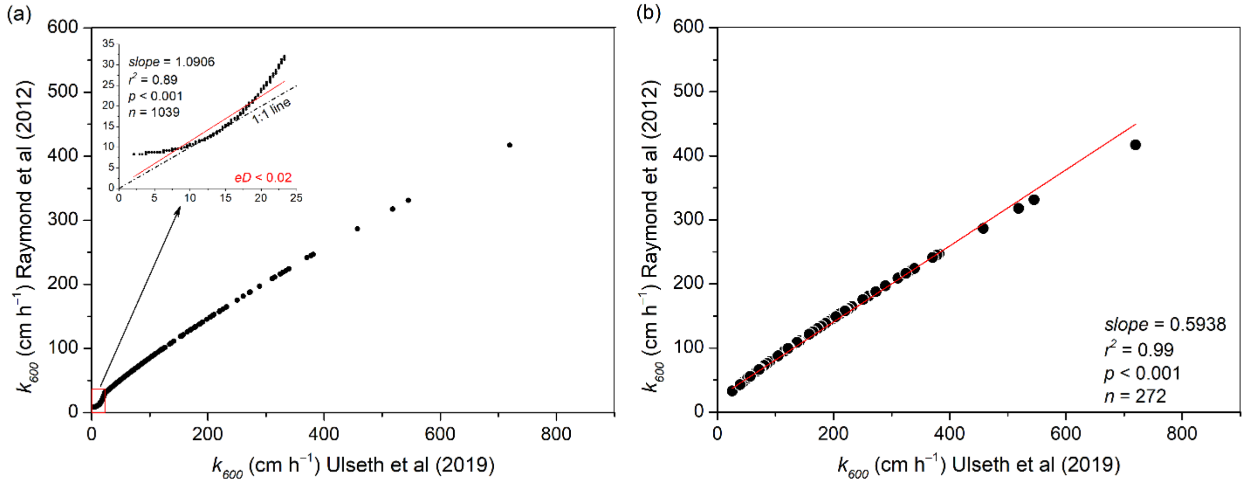

Figure 3.3 Comparison of dry season k_{600} estimated by the Ulseth and Raymond approaches. (a) all streams and rivers. (b) high energy streams only.

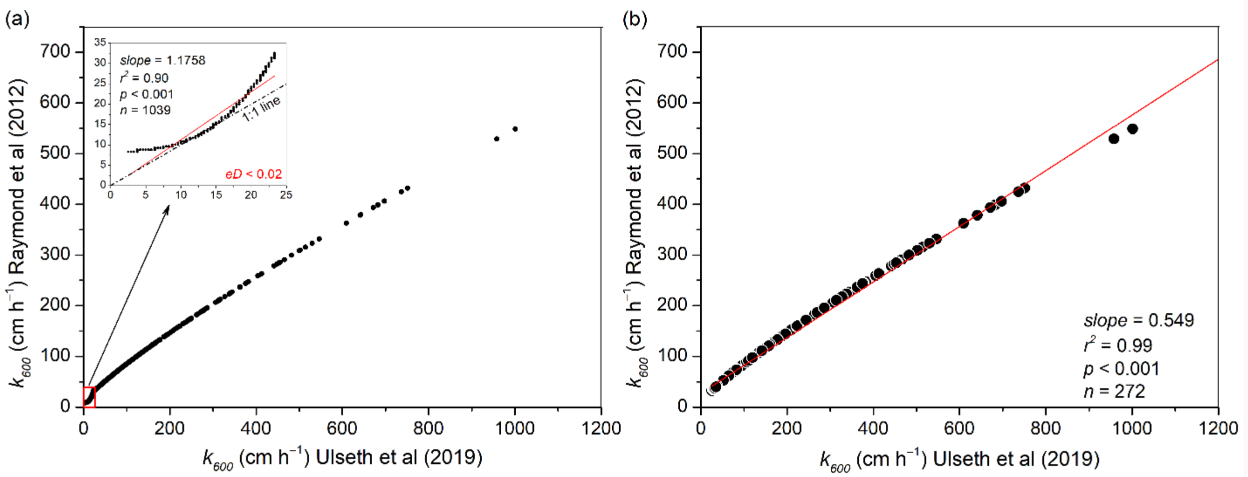

Figure 3.4 Comparison of wet season k_{600} estimated by the Ulseth and Raymond approaches. (a) all streams and rivers. (b) high energy streams only.

Using the same CO_2 emission measurement methods as in the Wuding and Dongjiang river basins, we conducted *in situ* measurement of CO_2 emissions using floating chambers in the Yellow River source region in 2016. The Yellow River source region is located on the northeastern edge of the Tibet Plateau region (Figure 1.1), which exhibits steep topography and high channel slope. Here we selected the sites with the *eD* > 0.02 m^2 s^{-3} and validated the performance of the equation developed by Ulseth et al (2019)[41] in estimating the calculated k_{600} based on floating chambers (Figure 3.5a). We observed that *eD* has a high explanatory power in predicting k_{600} and the empirical model proposed by Ulseth et al (2019) for high-energy streams is able to predict their gas transfer velocities (measured k_{600} versus modeled k_{600}: $r^2 = 0.59$, slope = 0.89; Figure 3.5b).

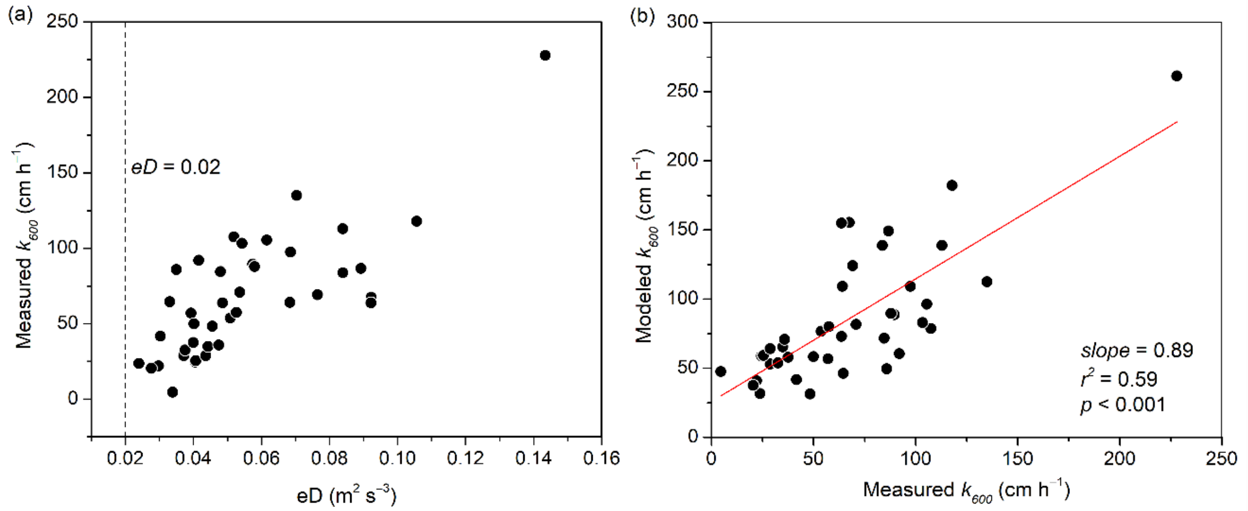

Figure 3.5 Predicting k_{600} for high-energy streams on the Tibetan Plateau. (a) Positive relationship between measured k_{600} and channel energy dissipation rate (*eD*), (b) Comparison of measured k_{600} and predicted k_{600} using Equation 2 of the Ulseth approach. Extreme k_{600} values in turbulent streams were removed from the analysis because of the errors in chamber deployments.

In view of the widespread applicability, in this analysis we used the Raymond approach to estimate the k_{600} in streams and rivers across China, upon which we calculated the flux of CO_2 evasions from streams and rivers. Because we aimed to consider the seasonal variations in CO_2 emissions as outlined above, we modeled the k_{600} in the dry and wet seasons. For the sampling sites without flow velocity records, mainly in the 2010s and accounting for ~29% of all observations, we used the flow velocity measured simultaneously at a nearby hydrologic gauge that was located on a river with the same Strahler order and within the same region to estimate the gas transfer velocity. For these sites, we used the stream lines from our river network data and the Shuttle Radar Topography Mission (SRTM)[42] DEM data to generate river elevation profile and then determined the mean stream channel slope. To evaluate the reliability of our flux estimates, we also estimated the k_{600} for each sampling site using the Ulseth approach. We first calculated *eD* for all sampling sites and for the dry and wet seasons, and then selected the suitable model (Equations 2 and 3) for k_{600} prediction. We compared the flux estimates based on the Raymond approach with that based on the Ulseth approach to evaluate the uncertainties (see Section 7 below for more discussion).

3.2 Gas transfer velocity in lakes and reservoirs

For lentic systems of lakes and reservoirs, CO_2 emissions are controlled by near-surface turbulence primarily generated by wind[14, 43, 44, 45]. In this study, the *k* value was calculated from three empirical relationships developed for different wind conditions in lentic systems and normalized using a Schmidt number of 600.

$$k = k_{600} \times (\mathrm{Sc}/600)^{-1/2 \text{ or } -2/3} \qquad (4)$$

where *Sc* is the Schmidt number and the Schmidt number of 20 °C in freshwater is 600, and the updated *Sc* is computed as a function of water temperature (*T* in degrees Celsius)[46]:

$$Sc = 1923.6 - 125.06\times T + 4.3773\times T^2 - 0.085681\times T^3 + 0.00070284\times T^4 \qquad (5)$$

The first relationship (Equation 6) was updated by Wanninkhof (2014)[46] from the Wanninkhof (1992) model [45] that had been widely used for computing CO_2 emission[47, 48 49]. The relationship between *k* and wind speed can be expressed as

$$k_{600} = 0.251 \times U_{10}{}^{2} \qquad (\mathrm{U_{10}}\text{: 3–15 m s}^{-1}) \qquad (6)$$

The second empirical model (Equation 7) was developed by Cole and Caraco (1998)[43] and the third model (Equation 8) by Crusius and Wanninkhof (2003)[50], which can be expressed as

$$k_{600} = 2.07+0.215 \times U_{10}{}^{1.7} \qquad (\mathrm{U_{10}} < 3.7 \text{ m s}^{-1}) \qquad (7)$$

$$k_{600} = 0.168+0.228 \times U_{10}{}^{2.2} \qquad (\mathrm{U_{10}}\text{: no specific requirement}) \qquad (8)$$

Because of the great water temperature variation across China, the *Sc* number ranged between 320 and 1600. We used the power exponent of -2/3 for low wind scenarios (<3.7 m s^{-1}) under the assumption that the water surface was smooth, and an exponent of -1/2 for high winds. Because of the wind effect on near-surface turbulence, we employed Equation 6 to calculate the *k* value

for strong winds (>3.7 m s^{-1}) and Equation 7 for winds <3.7 m s^{-1}. Meanwhile, the *k* estimates based on Equation 8 were used for error analysis.

Monthly wind speeds measured at 10 m height were retrieved from the National Meteorological Information Center of the China Meteorological Administration (http://data.cma.cn). We collected wind records at 686 meteorological stations across China for the 1980s and 1112 stations for the 2010s. The wind was measured at 10 m height following national standards of the "specifications for surface meteorological observation" (QX/T 45–2007)[51]. The annual mean wind speed varied in the range of 0.4–11.6 m s^{-1} and 0.4–8.9 m s^{-1} in the 1980s and 2010s, respectively. Wind speed was directly used for *k* prediction if a meteorological station was located within 2 km from a hydrologic gauge (in the 1980s) or a sampling site (in the 2010s). Otherwise, the ordinary kriging interpolation was performed using ArcGIS 10.3 software ((ESRI Inc., Redlands, CA, USA) to estimate the wind speed at the hydrologic gauges or sampling sites (Figure 3.6).

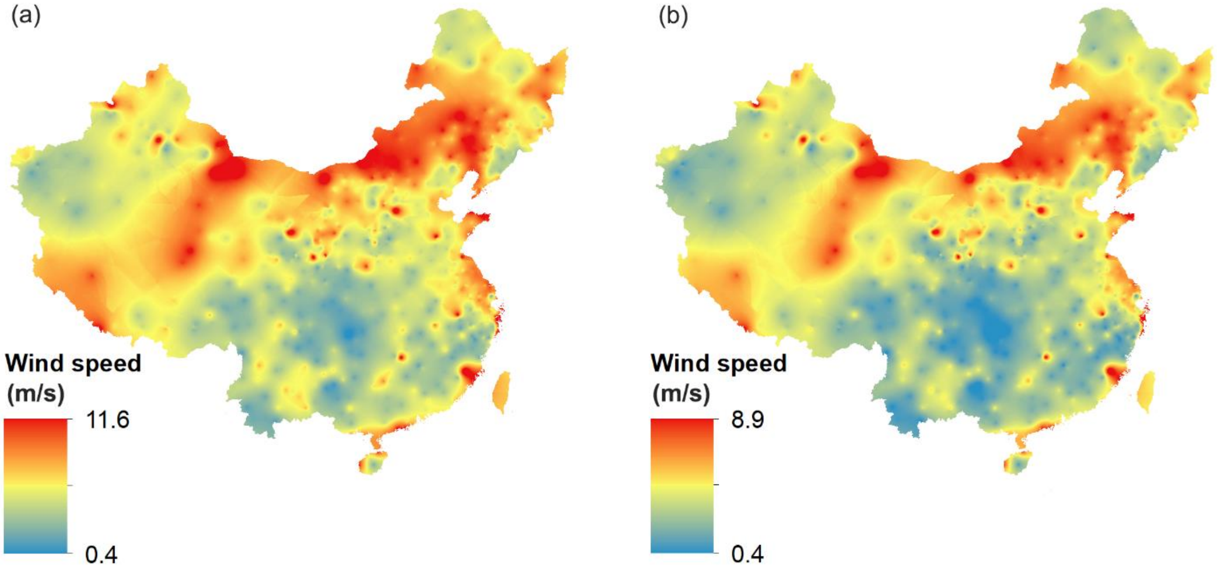

Figure 3.6 Spatial variations of wind speed at 10 m height across China in the 1980s (a) and 2010s (b).

To verify the reliability of the empirical models in predicting the *k* value in Chinese lakes and reservoirs, we compared the modeled *k* values against direct measurements in lakes and reservoirs in the Huang-Huai-Hai (Ran et al (2017)[26]), Greater Pearl (Ran unpublished), NE China (Ran unpublished) and Tibetan Plateau (Tian et al (2019)[28]) regions by using floating chambers (see their location in Figure 2.4). The models tend to underestimate the actual k_{600} values by ~20% (Figure 3.7), consistent with our earlier results[26]. This error was considered in the propagated error analysis (see Section 8 below for more discussion).

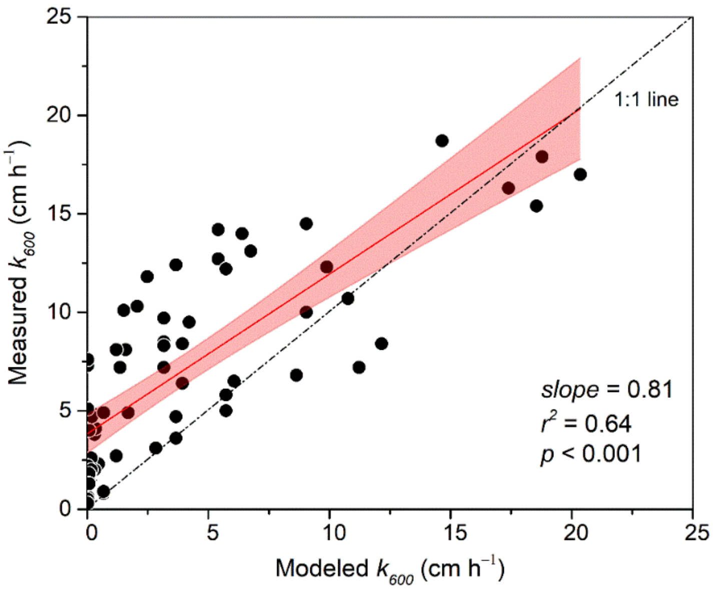

Figure 3.7 Comparison of modeled k_{600} values against measured k_{600} values based on floating chamber deployments under different climatic and hydrologic conditions. The red band represents the 95% confidence interval (95%CI) of the predicted *k*.

4. Surface area of Chinese inland waters

Areal extent of water surface determines the spatial dimension of CO_2 emissions across the water-air interface. In order to estimate CO_2 efflux in the 1980s and 2010s, we calculated the surface area of Chinese inland waters in the two time periods. Considering the huge climatic and hydrologic differences across China, we estimated the water surface area in each of the six regions by considering the changes in the dry and wet seasons (Table 1.1).

To delineate the inland waters, we obtained the Landsat satellite images with 30-meter spatial resolution (mainly Landsat 5 and 7). The results have been published in our earlier research work and a detailed description of the methodology can be found therein[9, 52, 53]. Briefly, a total of 507 satellite images with 30-m spatial resolution, including 412 Landsat Thematic Mapper (TM) images and 95 Enhanced Thematic Mapper Plus (ETM+) images, were used in this delineation. These images were mostly captured after the monsoon season during the years from 2005 to 2008. The overall procedure of image processing can be summarized into two steps. First, we developed an automated procedure that employed multiple thresholds to detect water bodies. Water bodies extracted from each image were then merged one by one using a batch processing algorithm to ensure that each water body can represent its complete natural status. Here we divided the water bodies into three categories: rivers (including artificial ditches), natural lakes and artificial reservoirs. In fact, it was extremely difficult to accurately identify the three types of water bodies just using computer algorithms. A script was developed in C# to assist the classification, and visual interpretation, if necessary, was used to reduce misclassification errors. The human-computer interaction computer application also integrated high-resolution satellite images and electronic maps of China. Using this application, we could display the shape and location of each water body as well as its corresponding high-resolution satellite base map or electronic map side by side. By comparing such reference information, our GIS technicians were able to determine the type of each water body. To guarantee the interpretation accuracy, we

arranged two GIS technicians to interpret separately. Re-examination was performed for the water bodies with inconsistent interpretation results. After classification, attributes of the water bodies, such as location, surface area and name, were added into the dataset for further analysis.

We also conducted a visual review of the extracted natural lakes and artificial reservoirs, aiming to merge small patches into adjacent larger lakes or reservoirs. For some lakes and reservoirs connected with rivers, separation operation was employed to detach them from rivers. In addition, some of the water bodies are smaller than their real size due to the low image quality (for example, frozen lake surface, snow cover on the water surface, or shadows cast by clouds). Therefore, the water classification accuracy can be guaranteed.

The major problem for the interpretation of rivers is that most small and medium-sized rivers extracted by remote sensing are disconnected and cannot represent their real status as they are relatively narrow, and could not be correctly identified due to the effects of shadows, clouds and even vegetation along the rivers. To resolve this problem, based on the classification, we first extracted the centerline as the initial river network. Using our developed script combined with electronic maps and high-resolution satellite images, the complete river networks were manually restored. Compared with the river network data extracted from DEM data, our river network data have the following incomparable advantages: 1). the river network data represents the real status of the Chinese river network; 2). the river network data includes both natural rivers and artificial ditches, which are very important to get an accurate estimate of CO_2 emissions from Chinese streams and rivers; 3). this data has been reviewed and calibrated by GIS technicians, and the dammed rivers occupied by reservoirs have been eliminated to avoid repetitive calculations of CO_2 emissions.

Figure 4.1 shows the comparison between the stream network obtained by DEM data and the real stream network obtained by this study. It can be seen from Figure 4.1A that, in the arid Teklimakan Desert in the NW China region, the river network acquired by DEM data displays a very dense river network, resulting in gross overestimation in the distribution of river networks. Likewise, such overestimations are very common in other arid and semi-arid areas in northern China, such as the Loess Plateau in Figure 4.1B. Another problem is that numerous artificial rivers and ditches in southern China cannot be correctly represented in the river network obtained by DEM as artificial rivers and ditches do not fully comply with the natural laws of hydrological processes (Figure 4.1C). However, such artificial rivers and ditches are accurately represented in the river network extracted in this study. In addition, in the rugged western China, the river network obtained by DEM data is excessively dense. As a result, many ephemeral rivers and/or flooded valleys were incorrectly identified as perennial rivers (Figure 4.1D).

The stream network data for the 1980s was obtained by backward updating the river network for the 2010s. The procedure was to update the state of rivers by referring to Landsat satellite images acquired in the 1980s with the assistance of the aforementioned script. If a river segment in the 1980s was longer than that in the 2010s, the river was flagged and then extended manually. If the river segment is an artificial river or ditch newly built after the 1980s, the river segment was deleted accordingly. Overall, in the arid and semi-arid northern China, there were more and denser rivers in the 1980s than in the 2010s. In the southern China, however, artificial ditches and rivers have increased significantly since the 1980s. In the First National Census of Water, it

was reported that more than 28,000 of these river segments were lost over the past decades[54]. Despite the disparity in numbers caused by inaccurate estimates in the past, it revealed a basic fact that many Chinese rivers have experienced shrinking due to climate change and human water use over the past decades[55]. Unfortunately, the report just provided the number of disappeared rivers and did not report the total length of these disappeared rivers.

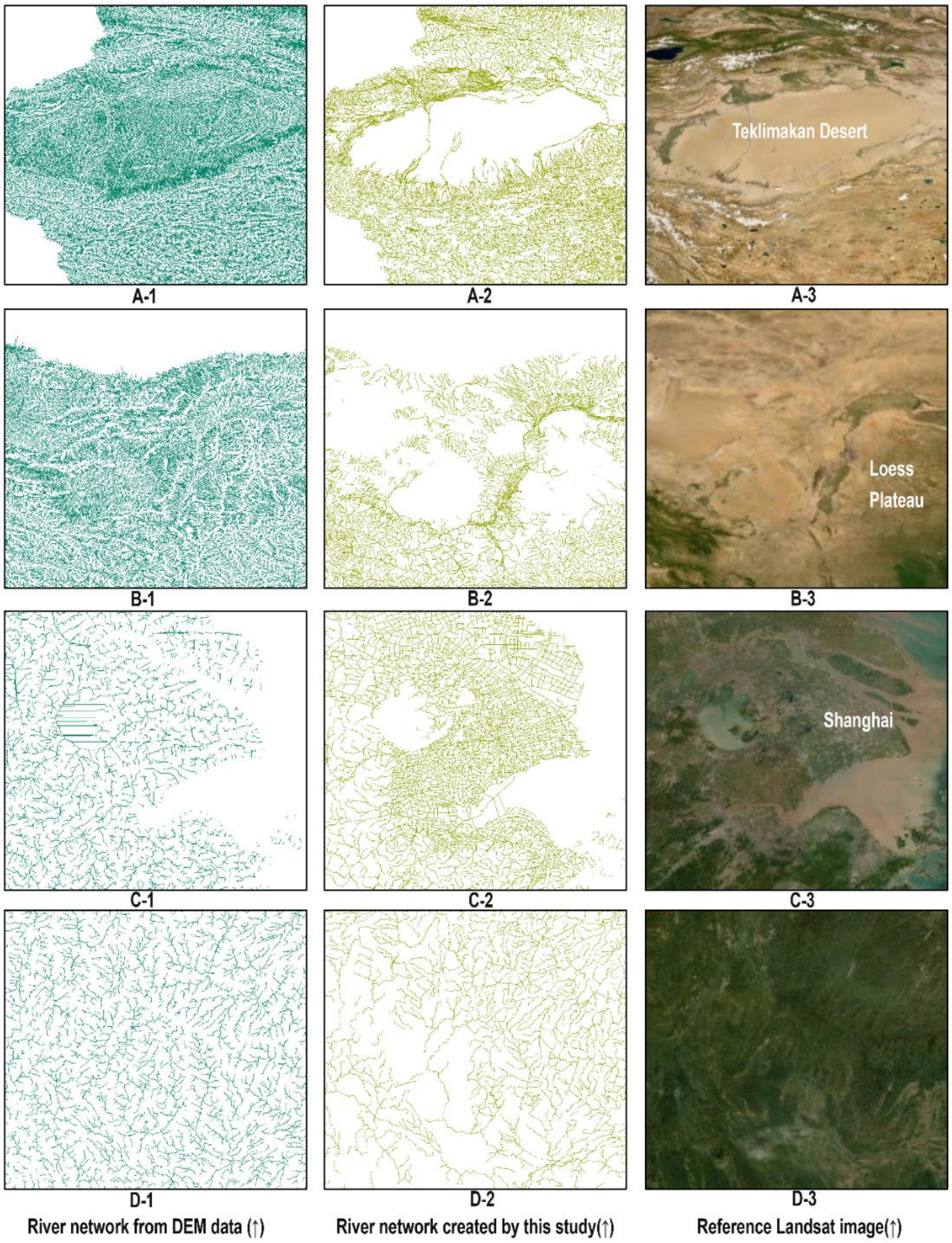

Figure 4.1 Comparison of the extracted stream network derived from DEM data and the real stream network created by this study: A. overestimation in the distribution of stream network in the arid Teklimakan Desert (in the NW China region); B. overestimation in the distribution of stream network derived from DEM data in arid and semi-arid areas in north China (in the Huang-Huai-Hai region); C. false identification of artificial rivers and ditches in DEM-derived stream network around the city of Shanghai in south China (in the Yangtze region); D. flooded valleys were incorrectly identified as perennial rivers in the stream network derived from DEM data.

We then assigned a numeric stream order to each segment of rivers according to Strahler's ordering system[56]. Based on the stream network map, we calculated the lengths and numbers of all stream and river channels for each Strahler order in each region and throughout China. The fluvial network of Chinese streams and rivers was shown in Figure 4.2. There were 8 Strahler orders. The only river segment classified as the 8th Strahler order is the mainstem channel of the Yangtze River, and the mainstem channels of other large rivers, such as the Yellow (Huang Ho), Pearl (Zhujiang) and Heilong (Amur) rivers, are classified as the 7th Strahler order. These results are consistent with earlier results which were testified by field surveys[26, 57], but one order lower than the widely used HydroSHEDS database which was derived from elevation data of the SRTM at 3" resolution[58, 59]. This was likely because many flooded valleys were incorrectly identified as rivers in the stream network derived from DEM data (Figure 4.1).

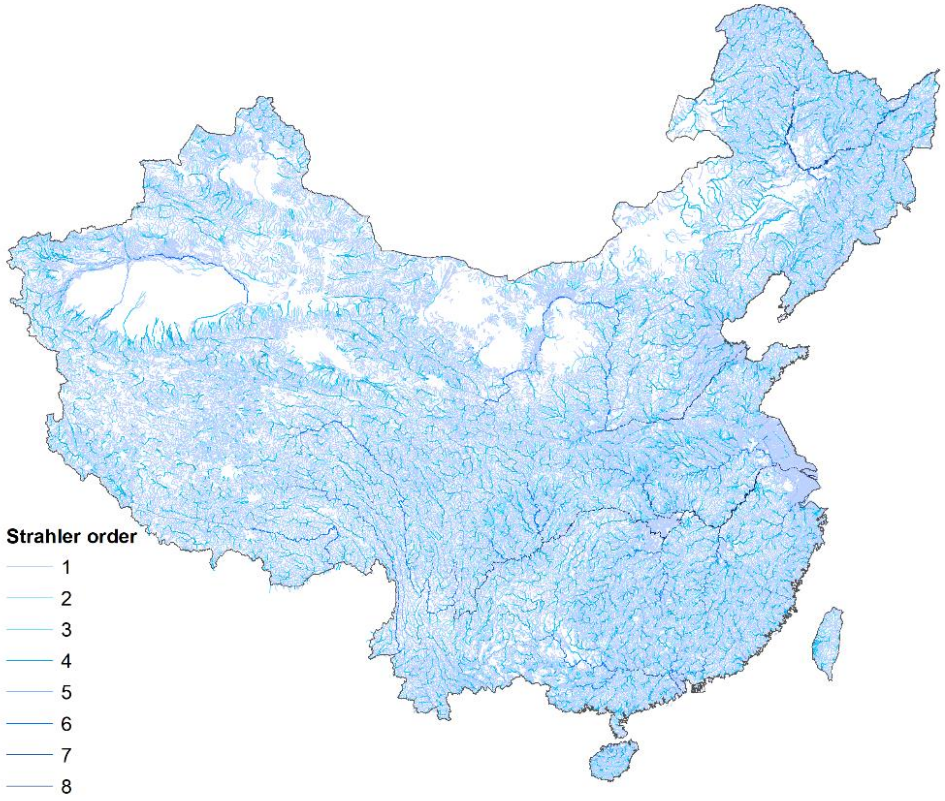

Figure 4.2 Fluvial network of streams and rivers across China.

Based on this stream network map, the spatial distribution of all stream and river sampling locations in the 1980s and 2010s was grouped based on their longitude and latitude information. Figure 4.3 presents the number of sampling locations for each Strahler order in the 1980s and 2010s. Approximately 50% of the sampling locations were situated in low Strahler order streams (e.g., 1st–3rd Strahler orders), which are largely proportional to the number of Strahler order streams within the stream network. In addition, there were 7 and 13 sampling sites in the middle and lower mainstem channel of the Yangtze River, the only 8th Strahler order stream within the stream network.

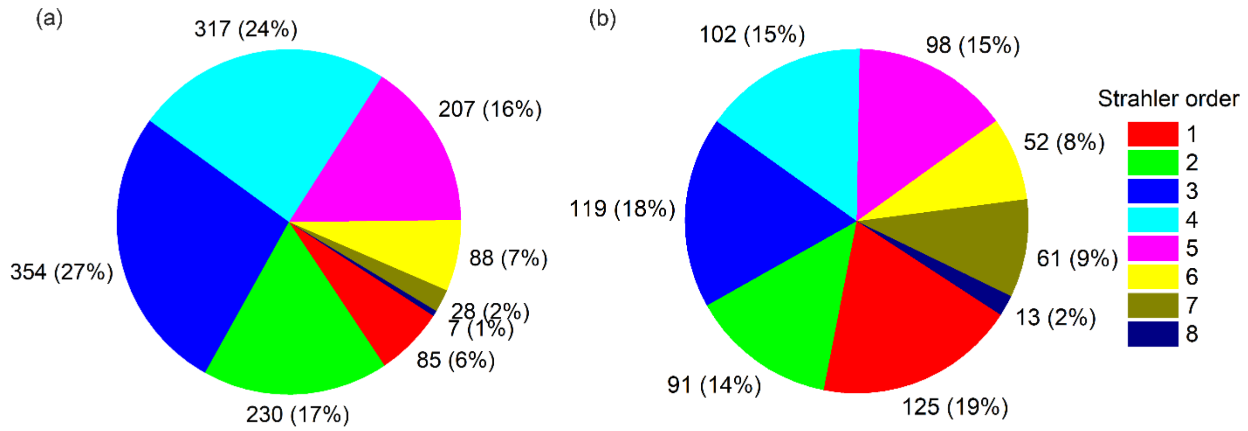

Figure 4.3 Spatial distribution of river sampling locations across the 8 Strahler orders in the 1980s (a) and 2010s (b). The numbers refer to the number of sampling sites and the percentages denote the percentage accounting for all sites (i.e., 1,316 in the 1980s and 661 in the 2010s).

Nevertheless, for stream width that typically ranges from less than 1 m in headwater streams to more than 1,000 m in mainstem channels, it is unreliable to use satellite images or DEM models to predict the stream width changes during the two periods or during the dry and wet seasons due to large water level fluctuations. We used the stream width data measured at the 1,709 hydrologic gauges across China and compiled in the Hydrologic Yearbooks. Stream width was measured concomitantly with flow discharge on a high frequency (e.g., daily). For each Strahler order in each of the six regions, we calculated its average stream width in the dry and wet seasons based on the corresponding hydrologic gauge stations that were categorized into different Strahler orders using their spatial locations in the delineated stream network. The water surface area of streams and rivers was calculated per Strahler order by multiplying the average stream width per Strahler order by the summed length of each Strahler order in each region. The total water surface area was calculated by summing up the water surface area of all Strahler orders in all the six regions. Because of the seasonal difference of stream width, we separately calculated the water surface area in the dry and wet seasons to detect its seasonal variations. Along with the pCO_2 and k values estimated for the dry and wet seasons, we were able to estimate the CO_2 evasion from streams and rivers in the dry and wet seasons (see Section 5).

For the lakes and reservoirs in the 1980s, we realized that the satellite images captured in that period showed low spatial resolution and the delineation results were characterized by high uncertainties, especially for small water bodies. In order to estimate the surface area of lakes and reservoirs, we used the national inventories which provide detailed information on the number, size (storage and surface area) and locations of lakes and reservoirs (see Section 4.1.2 below). There were 2,871 lakes with the surface area larger than 1 km^2 (ref [4]). For small lakes (<1 km^2) which are undetectable from satellite images back to the 1980s, we assumed their number and surface area remained largely unchanged and used the estimates for the 2010s to estimate that in the 1980s. Because the surface area of small lakes accounted for only 8.3% of the total surface area of lakes in the 2010s, this assumption would not greatly bias the total surface area of lakes in the 1980s. For small reservoirs in the 1980s, we retrieved the number of these small reservoirs from national inventories[60, 61], but the surface area information is missing. We estimated their surface area by using the averaged surface area of small reservoirs in each region in the 2010s and assuming that their spatial distribution across regions (i.e., % of small reservoirs in each

region accounting for the number of all small reservoirs across China) is similar to that in the 2010s. We further assumed that the water surface area of lakes and reservoirs did not greatly change between the dry and wet seasons although it is subject to fluctuation because of changes in rainfall and temperature and, for reservoirs, dam operation. We estimated the water surface area errors of Chinese lakes and reservoirs based on the satellite image-based delineation uncertainty (8.1%, ref [52]).

4.1 Water surface area in the 1980s

4.1.1 Streams and rivers

Water surface area across the Strahler order was summarized in Table 4.1 (dry season) and Table 4.2 (wet season). Headwater streams (i.e., first- and second-order streams) represented 20.4–48.2% of the total stream surface area in the six regions. Because of excessive water withdrawal in the lower reach, headwater streams in the NW China region are exceptionally important, accounting for 48.2% and 47.5% of the total surface area in the dry and wet seasons, respectively. The stream surface area of different Strahler orders was then summed up for each region to compute the total water surface area of streams and rivers throughout China (Table 4.3). The total water surface area of streams and rivers in the dry season was 9,600 km^2 or 17% smaller than that in the wet season (Table 4.3). The Yangtze region had the highest water-land ratio among the six regions, whereas the NW China region showed the lowest ratio (0.16–0.21%) because of its extremely dry climate (Table 1.1).

Table 4.1 Water surface area of Chinese streams and rivers in the dry season of the 1980s across Strahler order. The numbers after the symbol '±' correspond to the s.d..

Strahler order	Greater Pearl (km^2)	Yangtze (km^2)	Huang-Huai-Hai (km^2)	NE China (km^2)	NW China (km^2)	Tibetan Plateau (km^2)
1	1432±844	3763±1058	1419±568	1388±621	647±346	2535±1014
2	1246±424	2023±1201	750±332	1156±1325	806±654	1701±775
3	1053±728	1926±1212	788±555	1158±750	677±519	1082±609
4	822±461	2122±1222	500±406	1851±1588	517±549	720±488
5	1205±817	1738±930	421±306	1536±1192	235±111	1119±873
6	717±455	2098±881	895±403	1111±190	132±53	1744±933
7	581±279	1175±601	559±267	4291±1056		
8		3848±1250				

Table 4.2 Water surface area of Chinese streams and rivers in the wet season of the 1980s across Strahler order. The numbers after the symbol '±' correspond to the s.d..

Strahler order	Greater Pearl (km^2)	Yangtze (km^2)	Huang-Huai-Hai (km^2)	NE China (km^2)	NW China (km^2)	Tibetan Plateau (km^2)
1	1854±1164	4445±1388	1809±638	1845±670	708±331	3280±1163
2	1498±580	2414±1311	937±436	1391±1490	1159±843	2031±840
3	1337±834	2296±1422	987±731	1454±873	922±632	1621±994
4	1037±549	2513±1348	610±436	2124±1666	666±609	936±665
5	1387±805	1915±935	517±387	1659±1199	304±138	1275±946
6	784±444	2228±908	1004±439	1168±185	175±57	1947±1008
7	613±270	1230±603	622±238	4427±1080		
8		3944±1250				

Table 4.3 Water surface area of Chinese streams and rivers in the 1980s. The numbers after the symbol '±' correspond to the s.d..

Region	Total Surface Area	Water Surface Area (km^2)		% of Total Surface Area	
	(10^4 km^2)	Dry season	Wet season	Dry season	Wet season
Greater Pearl	75.5	7056±4008	8511±4646	0.93±0.53	1.13±0.62
Yangtze	136.2	18694±8355	20986±9163	1.37±0.61	1.54±0.67
Huang-Huai-Hai	124.1	5332±2836	6486±3306	0.43±0.23	0.52±0.27
NE China	175.5	12491±6720	14068±7162	0.71±0.38	0.80±0.41
NW China	190	3014±2231	3934±2610	0.16±0.12	0.21±0.14
Tibetan Plateau	258.3	8902±4691	11091±5616	0.34±0.18	0.43±0.22
Total/Mean	959.6	55488±12886	65076±14357	0.58±0.13	0.68±0.15

4.1.2 Lakes and reservoirs

Water surface area estimates for lakes in the 1980s were presented in Table 4.4. The Tibetan Plateau region was most abundant in lakes. The surface area of its lakes accounted for 51% of the total water surface area of all lakes across China. Overall, there were 2,871 lakes of ≥1 km^2 in the 1980s, of which 39% were located in the Tibetan Plateau region[4]. The total water surface area of all lakes combined was estimated at 79,196±6415 km^2 in the 1980s, consistent with recent national estimates[8, 10]. With respect to the percentage of land surface occupied by lakes, the highest is in the Tibetan Plateau region (1.56%), followed by the Yangtze region (1.22%). The lowest was observed in the NW China region with 0.24%. On the national scale, lakes occupied 0.83% of the total surface area in the 1980s.

Dam construction in China experienced a rapid development during the period 1950−1980. Water surface area estimates for reservoirs in the 1980s were shown in Table 4.5. Approximately 87,000 reservoirs with a total storage capacity of 421 km^3 were completed by the 1980s[61] (see Figure 4.7a below), of which 97% were small reservoirs with the storage capacity below 0.01 km^3. The total water surface area of these reservoirs was estimated at 14,772±1196 km^2, of which about 88% was attributed to large reservoirs that accounted for only 0.4% of the number of reservoirs[60].

Table 4.4 Number and water surface area of Chinese lakes in the 1980s.

Region	Total Surface Area (10^4 km^2)	Water Surface Area (km^2)	% of Total Surface Area	No. of Lakes (≥ 1 km^2)	Water Surface Area of Lakes ≥ 1 km^2 (km^2)
Greater Pearl	75.5	1399.2±113	0.19	30	971
Yangtze	136.2	16563±1342	1.22	677	15444
Huang-Huai-Hai	124.1	6226.4±504	0.50	180	5260.5
NE China	175.5	10015.3±811	0.57	705	8400.3
NW China	190	4619.1±374	0.24	170	3924.7
Tibetan Plateau	258.3	40372.6±3270	1.56	1109	38328.2
Total/Mean	959.6	79196±6415	0.83	2871	72329

Table 4.5 Number and water surface area of Chinese reservoirs in the 1980s.

Region	Total Surface Area (10^4 km^2)	No. of Reservoirs	Water Surface Area (km^2)	% of Total Surface Area
Greater Pearl	75.5	22628	2171.8±176	0.29
Yangtze	136.2	43981	4943.7±400	0.36
Huang-Huai-Hai	124.1	12325	3441.8±279	0.28
NE China	175.5	6883	3193.8±259	0.18
NW China	190	783	449.3±36	0.02
Tibetan Plateau	258.3	232	571.1±46	0.02
Total/Mean	959.6	86832	14772±1196	0.15

4.2 Water surface area in the 2010s

4.2.1 Streams and rivers

We noticed that flow discharge in many Chinese streams and rivers, especially those in northern China, has been greatly reduced due to unsustainable development and climate change[62, 63, 64, 65]. Some small streams and rivers have even dried up as a result of fragmentation and flow regulation caused by massive construction of hydraulic engineering projects over the past decades[66]. To take account of these alterations to water surface area, we compared the hydrologic gauge-based stream width datasets between the two periods. Along with the delineated stream network for the 2010s, we calculated the surface area of streams and rivers in the six regions.

Seasonal results of water surface area of Chinese streams and rivers across Strahler order in the 2010s were shown in Tables 4.6 and 4.7, and the summarized results were shown in Table 4.8. Water surface in the wet season was 9.6–27.5% higher than that in the dry season with a national average of 14.3%. The highest seasonal change was observed in the NW China region while the lowest was in the Yangtze region. Drainage density was highest in the Yangtze region and lowest in the NW China region (Table 4.8). Overall, water surface area of streams and rivers accounted for 0.53% and 0.61% of the total land surface in the dry and wet seasons, respectively.

Table 4.6 Water surface area of Chinese streams and rivers in the dry season of the 2010 across Strahler order. The numbers after the symbol '±' correspond to the s.d..

Strahler order	Greater Pearl (km^2)	Yangtze (km^2)	Huang-Huai-Hai (km^2)	NE China (km^2)	NW China (km^2)	Tibetan Plateau (km^2)
1	1543±1036	3801±1599	877±426	1334±898	551±346	2720±1133
2	1323±746	2268±1449	712±757	1079±1350	631±515	1731±768
3	1043±763	1985±1511	676±607	971±762	529±408	1439±1188
4	802±481	1913±1350	587±758	1591±1631	453±469	824±503
5	915±813	1124±933	421±314	1392±1177	171±97	1189±921
6	372±439	1329±913	729±391	1056±194	115±55	1760±967
7	432±263	1026±602	443±269	4157±1059		
8		2987±1227				

Table 4.7 Water surface area of Chinese streams and rivers in the wet season of the 2010s across Strahler order. The numbers after the symbol '±' correspond to the s.d..

Strahler order	Greater Pearl (km^2)	Yangtze (km^2)	Huang-Huai-Hai (km^2)	NE China (km^2)	NW China (km^2)	Tibetan Plateau (km^2)
1	1857±1215	4354±1552	1228±497	1773±898	643±331	3378±1223
2	1512±795	2542±1483	838±811	1315±1350	808±645	2018±861
3	1232±839	2241±1600	836±764	1219±762	723±548	1737±1210
4	948±563	2107±1400	670±785	1803±1631	592±573	968±620
5	1009±813	1207±940	473±330	1511±1177	213±116	1278±1012
6	396±445	1391±931	803±413	1112±194	145±69	1989±1009
7	453±270	1059±606	492±245	4293±1085		
8		3112±1277				

Table 4.8 Water surface area of Chinese streams and rivers in the 2010s. The numbers after the symbol '±' correspond to the s.d..

Region	Total Surface Area	Water Surface Area (km^2)		% of Total Surface Area	
	(10^4 km^2)	Dry season	Wet season	Dry season	Wet season
Greater Pearl	75.5	6431±4541	7408±4940	0.85±0.60	0.98±0.65
Yangtze	136.2	16433±9584	18013±9789	1.21±0.70	1.32±0.72
Huang-Huai-Hai	124.1	4445±3521	5341±3845	0.36±0.28	0.43±0.31
NE China	175.5	11581±7071	13025±7097	0.66±0.40	0.74±0.40
NW China	190	2451±1890	3124±2282	0.13±0.10	0.16±0.12
Tibetan Plateau	258.3	9662±5481	11368±5934	0.37±0.21	0.44±0.23
Total/Mean	959.6	51003±14439	58279±15027	0.53±0.15	0.61±0.16

4.2.2 Lakes and reservoirs

Spatial distribution of lakes and reservoirs across China in the 2010s was presented in Figure 4.4, which was characterized by pronounced spatial variations. Water surface area of lakes in the six regions was summarized in Table 4.9. There were 187,917 lakes in China in the 2010s with the size varying from <0.1 km^2 to more than 4000 km^2. The total surface area of lakes is 82570±6688 km^2, which is 1.5-fold of that of the streams and rivers. In addition, it is important to note that the surface area of lakes with ≥1 km^2 accounts for 91.7% of the total surface area of lakes. The Tibetan Plateau region has the highest number of lakes, accounting for ~24% of all lakes combined, followed by the NE China (20%) and the Huang-Huai-Hai (18%) regions (Table 4.9). However, with respect to the percentage of land surface occupied by lakes, the highest is in the Tibetan Plateau region (1.74%), followed by the Yangtze region (1.09%), and the lowest is in the Greater Pearl region (0.14%). As a result, the lakes in the Tibetan Plateau region account for more than half of the total lake surface area. On the national scale, lakes occupied 0.86% of the total surface area in the 2010s, slightly higher than that in the 1980s.

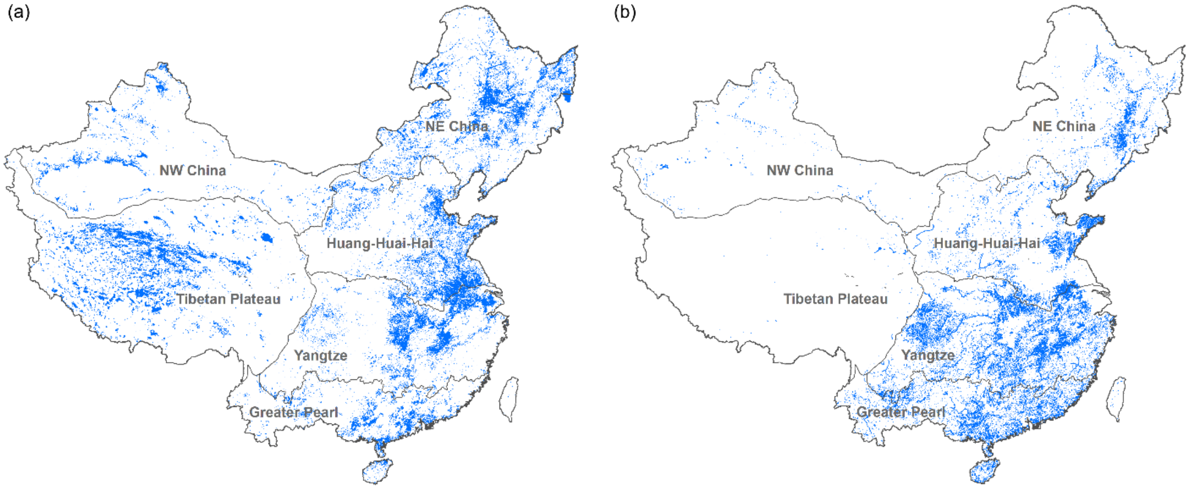

Figure 4.4 Spatial distribution and abundance of lakes (a) and reservoirs (b) in China. Adapted from Yang and Lu (2014)[52]. Because Yang and Lu (2014) only reported the results of lakes and reservoirs in Mainland China, lakes and reservoirs in Taiwan were delineated from Landsat 5 and 7 satellite images using the same methods as in Yang and Lu (2014)[52] and described above.

Table 4.9 Number and water surface area of Chinese lakes in the 2010s. Adapted from Yang and Lu (2014)[52]. The lakes in Taiwan are included into the Greater Pearl region.

Region	Total Surface Area (10^4 km^2)	No. of Lakes	Water Surface Area (km^2)	% of Total Surface Area	No. of Lakes (≥ 1 km^2)	Water Surface Area of Lakes ≥ 1 km^2 (km^2)
Greater Pearl	75.5	24346	1021.6±83	0.14	32	593.4
Yangtze	136.2	33960	14854.7±1203	1.09	473	13735.7
Huang-Huai-Hai	124.1	34166	6231.1±505	0.50	216	5265.2
NE China	175.5	36687	9881.4±800	0.56	683	8266.4
NW China	190	13290	5527.1±448	0.29	300	4832.7
Tibetan Plateau	258.3	45468	45054±3649	1.74	1271	43009.6
Total/Mean	959.6	18,7917	82,570±6688	0.86	2,975	75,703

There were 89,622 active reservoirs with a total surface area of 25,616±2075 km^2 by 2010 (Table 4.10). Approximately 91% of the reservoirs are constructed in the Greater Pearl, Yangtze and Huang-Huai-Hai regions, and accordingly, the surface area of the reservoirs in these three regions accounts for 74% of the total reservoir surface area. The largest reservoir is the Three Gorges Reservoir (686 km^2) on the middle Yangtze River, close to recent surface area estimates of the reservoir[67]. In contrast to lakes (Table 4.9), there are only 239 reservoirs in the Tibetan Plateau region with a total surface area of 696 km^2. In addition, low flow discharge in the NW China rivers has constrained dam building (Table 4.10), which accounts for only 0.9% and 6.6% of the number and surface area, respectively, of reservoirs across China.

Table 4.10 Number and water surface area of Chinese reservoirs in the 2010s. Adapted from Yang and Lu (2014)[52]. The reservoirs in Taiwan are included into the Greater Pearl region.

Region	Total Surface Area (10^4 km^2)	No. of Reservoirs	Water Surface Area (km^2)	% of Total Surface Area
Greater Pearl	75.5	23347	4601.6±373	0.61
Yangtze	136.2	45401	9739.4±789	0.72
Huang-Huai-Hai	124.1	12722	4626.8±375	0.37
NE China	175.5	7105	4257.1±345	0.24
NW China	190	808	1695.3±137	0.09
Tibetan Plateau	258.3	239	695.7±56	0.03
Total/Mean	959.6	89,622	25,616±2075	0.27

4.3 Comparison of water surface area between 1980s and 2010s

If the averaged surface area estimates for streams and rivers in the dry and wet seasons were used, the total water surface area of all rivers/streams, lakes and reservoirs in the 1980s and 2010s was summed at 154,250 and 162,826 km^2, respectively. Our surface area estimates are consistent with the result reported by the Statistical Yearbook of China (~170,000 km^2)[68] with a difference of 4–9%. Despite the consistency, it is important to note that the inland water area estimate in the statistical report has not been updated for nearly 10 years. Comparison of water surface area across the six regions and between the two periods was presented in Table 4.11 and comparison of the total surface area was shown in Figure 4.5. Compared with the 1980s, the stream surface area has declined by 9%, while the surface area of reservoirs has increased by 73% (Table 4.11). For streams and rivers, their surface area decreased in five of the six regions, due largely to excessive water withdrawal for irrigation and industry, continuous dam building and decreased precipitation caused by climate change[7, 55, 69]. The only region showing increased water surface area was the Tibetan Plateau because of melting glaciers and permafrost due to climate change[8, 10]. Particularly, the higher increase in stream surface area in the dry season (8.5%) than in the wet season (2.5%) is likely because of the higher rates of temperature and precipitation increase in the dry season than in the wet season. Recent studies have clearly indicated that both temperature and precipitation on the Tibetan Plateau increased at a faster rate in the dry season than in the wet season[70, 71]. As a result, the more rapid melting of glaciers, snow and permafrost and the higher precipitation increase in the dry season have probably resulted in the higher stream runoff and thus the higher stream water surface. The largest reduction of stream surface area occurred in the NW China region (Table 4.11), which was in line with the rapid expansion of irrigation (thus reservoirs) and decreasing precipitation[69]. The percentage of surface area of streams and rivers is positively related to precipitation (Figure 4.6). The Greater Pearl and Yangtze regions in southern China exhibit a higher percentage of surface area than the regions with low precipitation. The NW China region is characteristic of the lowest drainage density due to its extremely low precipitation (Table 1.1 and Figure 4.6).

Table 4.11 Change of water surface area between the 1980s and 2010s, expressed as % relative to the 1980s.

Region	Rivers		Lakes	Reservoirs
	Dry season	Wet season		
Greater Pearl	-8.9	-13	-27	111.9
Yangtze	-12.1	-14.2	-10.3	97
Huang-Huai-Hai	-16.6	-17.7	0.1	34.4
NE China	-7.3	-7.4	-1.3	33.3
NW China	-18.7	-20.6	19.7	277.3
Tibetan Plateau	8.5	2.5	11.6	21.8
Mean	-8.1	-10.4	4.3	73.4

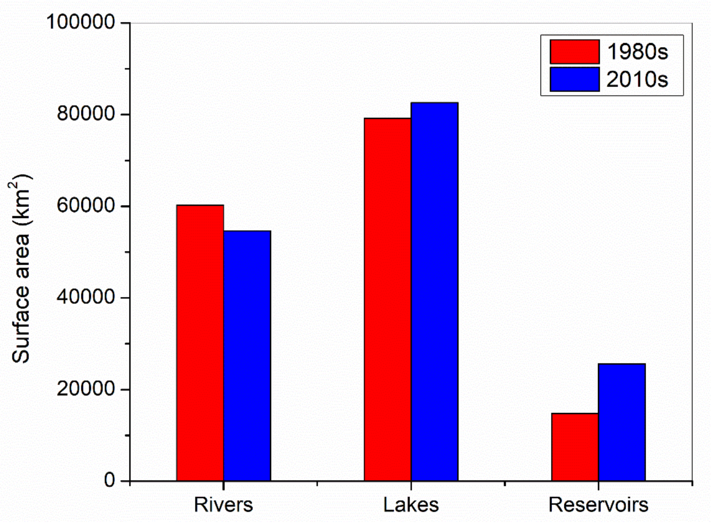

Figure 4.5 Comparison of surface area of streams/rivers, lakes and reservoirs in China between the 1980s and 2010s.

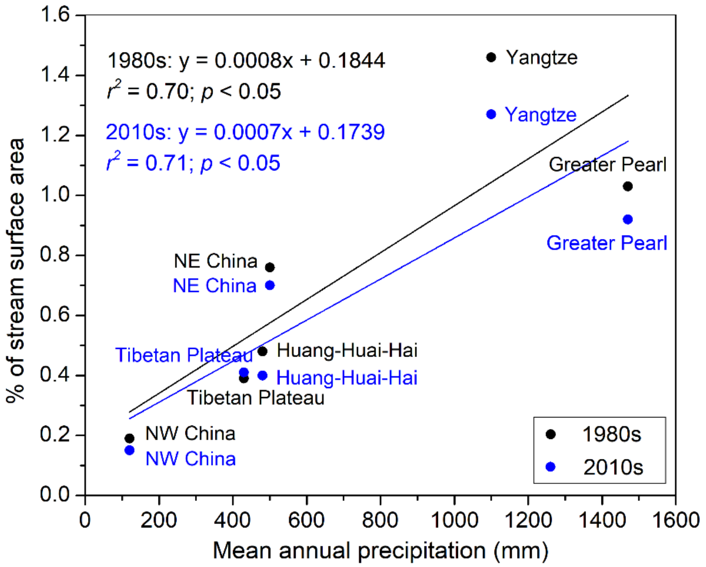

Figure 4.6 Relationship between mean annual precipitation and the percentage of surface area of streams and rivers within the six regions in the 1980s and 2010s. Note that because the overall annual precipitation across China has not notably changed in the past three decades[1], we used the long-term mean annual precipitation for both the 1980s and 2010s scenarios.

China has engaged in a dam boom since the 1980s with surging economy and deteriorating air quality that spurred the need for clean energy. Figure 4.7 illustrates the history of reservoir construction in China during the period 1973–2018. Approximately 15,000 new reservoirs were completed between the two periods and the storage capacity more than doubled. Large dams were constructed at a rate unmatched in human history. As a result, flowing waters were continuously converted to reservoirs and accordingly, the surface area of reservoirs increased rapidly in all the six regions (Table 4.11). Major water surface increases were observed in southern China, including Greater Pearl and Yangtze, due to massive dam construction during the past decades driven by high energy need and rapid economic development.

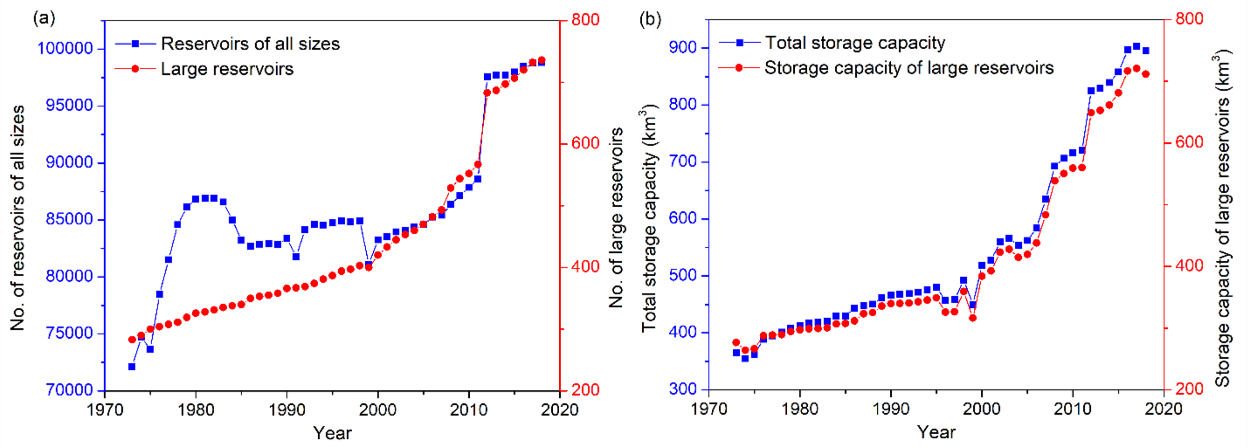

Figure 4.7 History of reservoir construction in mainland China. (a) Number of reservoirs of all sizes and large reservoirs, (b) Storage capacity of all reservoirs and large reservoirs. Large reservoirs in China are the reservoirs with the storage capacity exceeding 0.1 km^3. Data source: Ministry of Water Resources of China (http://www.mwr.gov.cn/english/publs/).

For the surface area of lakes, its temporal change exhibited pronounced spatial variations although the total surface area has slightly increased by around 4% compared with that in the 1980s (Table 4.11). Both the number and surface area of lakes in the Tibetan Plateau region showed an increasing trend because of enhanced snowmelt and glacier retreat caused by global warming, consistent with recent studies by Zhang et al[8] and Yang et al[9] which found significant lake expansion on the Tibetan Plateau. A slight increase in number and large increase in surface area of lakes were also observed in the NW China region, which can be partly explained by the increased glacier-fed lakes[10]. In contrast, both lake number and surface area of lakes in other regions, especially in south China (the Yangtze and the Greater Pearl regions), have greatly declined or remained largely unchanged. For example, approximately 1700 km^2 or ~10% of lake surface area has been lost in the Yangtze region and the largest reduction of lake surface area occurred in the Greater Pearl region (27%). Land reclamation and lake isolation due to greatly reduced river-lake connectivity resulting from dam impoundment are the primary causes[6, 72]. The reduced surface area of streams and rivers due to flow dry-up and conversion to dams has been offset by increased lake and reservoir surface area. As a result, the total surface area of Chinese inland waters has increased by about 8600 km^2 on average or 6% based on the 1980s level.

5. CO_2 efflux calculation

5.1 Areal CO_2 efflux

For pCO_2 calculated from water chemistry records at discrete sampling sites, the areal CO_2 efflux (F_{CO2} in units of mmol m^{-2} d^{-1}) across the water-air interface can be expressed as

$$F_{CO2} = k \times k_H \times \Delta pCO_2 \quad (9)$$

where k is the gas transfer velocity (cm h^{-1}), k_H is Henry's constant for CO_2 corrected for temperature and pressure (mol L^{-1} atm^{-1}) and ΔpCO_2 is the water-air gas concentration gradient[73]. Whereas a positive gradient corresponds to an emission of CO_2 from the water to the atmosphere, a negative value indicates a carbon invasion into the water.

To calculate the CO_2 efflux in the 1980s and 2010s, we used the average annual atmospheric pCO_2 of the two periods, which were retrieved from the Global Greenhouse Gas Reference Network of the Earth System Research Laboratory (ESRL), National Oceanic and Atmospheric Administration (NOAA)(https://esrl.noaa.gov/). The average atmospheric pCO_2 was 345 and 400 µatm in the 1980s and 2010s, respectively. In addition, atmospheric pCO_2 changes with altitude were considered for the computation, especially for the Tibetan Plateau region where the elevation is generally >3000 m a.s.l..

5.2 Total CO_2 efflux

We separately calculated the flux of CO_2 emissions from lotic systems (i.e., free-flowing streams and rivers) and lentic systems (i.e., lakes and reservoirs). The sampling sites were categorized into the six defined regions based on their spatial location (Figure 2.1 for the 1980s and Figure 2.4 for the 2010s) and further grouped into different stream orders for streams and rivers or into different size classes for lakes and reservoirs (see Section 5.2.2 below). Chinese streams and rivers are characterized by pronounced seasonal variability in hydraulic geometry and carbon export[74, 75]. Because the seasonal variations in pCO_2, k value and water surface area, we separately calculated CO_2 efflux in the dry and wet season for each region. When a river segment is dammed, a stagnant water body, thereafter a reservoir, will be formed. Because the physicochemical properties of reservoirs are analogous to that of lakes, in this study we used the observed pCO_2 or areal CO_2 efflux (F_{CO2}, see Section 5.2.2 below) results from lakes in a given region to represent that from reservoir waters in that region, and vice versa. However, we reported the total CO_2 efflux from lakes and reservoirs separately based on their respective surface area. Finally, the total CO_2 efflux from inlands waters across China was calculated by summing up the CO_2 effluxes from lotic and lentic systems. These calculation steps were repeated for the studied two periods.

5.2.1 CO_2 efflux from streams and rivers

With areal CO_2 effluxes (F_{CO2} in units of mmol m^{-2} d^{-1}) at individual sampling sites in both the dry and wet seasons, we calculated total CO_2 efflux by Strahler order within each of the six regions according to:

$$F_{CO2total} = \sum_{region}\left[\sum_{SO}\left(\sum_{season} \bar{F}_{CO2} \times SA \times N \times 12 \div 10^{15}\right)\right] \quad (10)$$

where $F_{CO2total}$ is the total CO_2 efflux from all streams and rivers across China (Tg C yr^{-1}), SO is the Strahler orders within a given region, $\bar{F}_{CO2}$ is the mean F_{CO2} of discrete sampling location-based results for a given SO within a given region, SA is the surface area of streams and rivers in

the dry and wet seasons (m^2), N is the number of days in the dry or wet season considering only the ice-free period, 12 is the molar mass of carbon (12 g mol^{-1}) and 10^{15} is the convertor from milligrams (mg) to teragrams (Tg).

5.2.2 CO_2 efflux from lakes and reservoirs
For lakes and reservoirs in each region, we computed the areal CO_2 efflux using wind-dependent *k* estimates (see Section 3.2 above). In view of the differential effects of convection and wind shear on near-surface turbulence[76], we split lakes and reservoirs into three size classes based on water surface area, namely <10 km^2, 10–50 km^2 and >50 km^2. The CO_2 efflux from lakes and reservoirs in each region was calculated by multiplying the mean seasonal (dry and wet seasons) areal CO_2 efflux per size class by the water surface area of all lakes or reservoirs of that class in that region. The CO_2 effluxes in the dry and wet seasons for each size class were then summed up to calculate the annual efflux for that size class. The CO_2 effluxes of all size classes were aggregated to derive the total CO_2 efflux from lakes or reservoirs in that region. Briefly, we calculated the total CO_2 efflux from all lakes or reservoirs across China according to

$$F_{CO2total} = \sum_{region}\left[\sum_{class}\left(\sum_{season}\bar{F}_{CO2} \times SA \times N \times 12 \div 10^{15}\right)\right] \quad (11)$$

where $\bar{F}_{CO2}$ is the mean F_{CO2} of discrete sampling results for a given water surface class within a given region, SA is the water surface area of the lakes or reservoirs within a given size class (m^2) and N is the number of days in the dry or wet season considering only the ice-free period.

Due to lack of sufficient data measurements in lakes and reservoirs in the Greater Pearl region in the 1980s, we used the adjacent lake and reservoir measurements in the Yangtze region (Figure 2.1) to estimate the CO_2 emissions from lakes and reservoirs in the Greater Pearl region. All the lakes and reservoirs in the 1980s were sampled multiple times across the year; for these sites, we calculated the average areal CO_2 efflux in the dry and wet seasons as much as possible. For the 2010s, the majority of lakes and reservoirs (252 out of 403) were also measured multiple times and 37% of them were measured only once, of which ~45% were performed in the dry season and ~55% in the wet season. To scale up to seasonal efflux estimation, for those measured only once, we assumed the areal effluxes obtained in the dry season were representative of those in the wet season, and vice versa. Considering the largely equal distribution of the measurements between the two seasons, this assumption may not greatly bias the seasonal efflux estimates. In addition, our efflux estimates discounted for the freezing period (80–120 days[3]; usually from late October to early March) for the NE China, NW China and the Tibetan Plateau regions (Table 1.1). This is because roughly one third of the measurements in these three regions were conducted during spring melt; for these measurements, the areal CO_2 evasion estimates that were generally elevated[77] have already accounted for the buildup of CO_2 during the freezing period.

6. Spatial and temporal variations of CO_2 emissions

6.1 River pCO_2 change along the stream network

Spatial distribution of stream water pCO_2 across Strahler stream orders in the 1980s are presented in Figure 6.1. Chinese streams and rivers exhibit a clear dominance of CO_2 oversaturation with respect to the overlying atmosphere. The surface water pCO_2 generally decreased from headwater streams towards the mainstream waters, showing an average decrease of 89 µatm in the dry season and 93 µatm in the wet season with increasing Strahler order for the

entire dataset. However, the relationship varied from 417 µatm (dry season) and 422 µatm (wet season) by Strahler order in the Huang-Huai-Hai region to a slight increase in the NE China and Tibetan Plateau regions (Figure 6.1). The positive increase of pCO_2 in the NE China region, especially in the mainstem channels, is likely because of the input of large quantities of CO_2 into rivers from wetlands which are widespread in the lower Songhua River basin[78, 79], the largest river in the NE China region. This overall decreasing trend of pCO_2 in the dry and wet seasons is consistent with the rapid evasion of CO_2 during fluvial transport within the stream network. Because 65% of dataset for the 2010s directly reports the areal CO_2 evasion rate and surface water pCO_2 values are not available, we did not plot the stream water pCO_2 across Strahler orders for the 2010s.

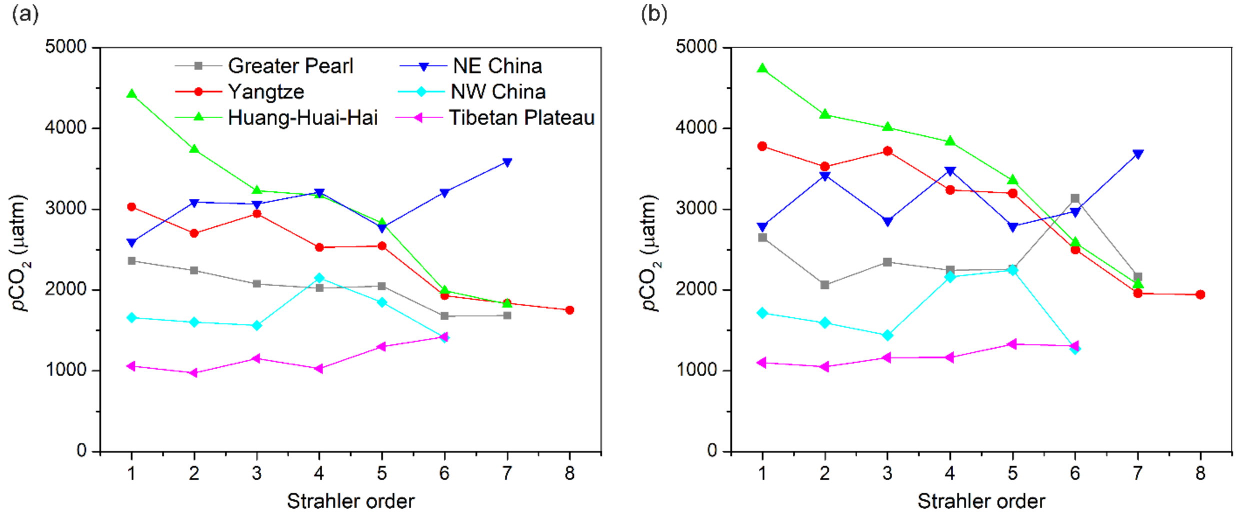

Figure 6.1 Spatial variation of stream water pCO_2 across Strahler orders in China in the 1980s. (a) dry season, (b) wet season.

6.2 Overall CO_2 emission fluxes

Outgassing fluxes of CO_2 from Chinese rivers/streams, lakes and reservoirs in the 1980s and 2010s are summarized in Tables 6.1, 6.2 and 6.3. We also calculated the annual average areal CO_2 efflux from streams and rivers and from lakes and reservoirs in each region based on the aggregated annual flux estimates and averaged surface area. These results are presented in Tables 6.1 and 6.3, respectively. Compared with the 1980s, the CO_2 efflux from streams and rivers in the 2010s declined in five of the six defined regions (Table 6.1). The largest reduction was observed in the dry NW China region due largely to greatly decreased stream surface area and F_{CO2}. As shown in Table 4.11, the stream network in the NW China region exhibited the largest reduction in surface area among the six regions in both the dry (-18.7%) and wet (-20.6%) seasons. In addition, the averaged F_{CO2} across the stream network of each region showed a decreasing trend between the two periods (Table 6.1) in both the dry (mean: 13±40%) and wet (mean: 32±28%) seasons. This can be partly attributed to the significantly reduced lateral carbon input into the river network during the past decades due to progressive implementation of vegetation restoration practices[80, 81] (see Section 6.4 below for more discussion). The intensity of soil erosion and the associated soil organic carbon transport have been greatly reduced with effective vegetation restoration across China[80, 82]. Meanwhile, river fragmentation and flow regulation by dams have reduced flow velocity, which has likely resulted in a lowered *k* value and therefore a smaller F_{CO2}. For example, Mei et al (2018)[83] reported a detectable flow velocity

decrease across the full range of flow discharge in the lower Yangtze River after the operation of Three Gorge Dam. The only region with increasing CO_2 efflux was the Tibetan Plateau region (Table 6.1). This is consistent with the expanding river network as a result of melting glaciers and permafrost (Table 4.11). Although the overall F_{CO2} declined in all six regions between the two periods, it is important to note that the F_{CO2} in the headwater streams (first- and second-order streams) in the Tibetan Plateau region increased by 9.6% (Table 6.1), which was probably because of the enhanced lateral carbon fluxes in response to climate change[84, 85]. Because the headwater streams account for 47% of the total surface area of the Tibetan Plateau stream network, their increasing F_{CO2} is responsible for the elevated total CO_2 efflux (Table 6.1).

Table 6.1 Change of CO_2 emissions from rivers/streams between the 1980s and 2010s.

Region	Annual mean F_{CO2} (mmol m^{-2} d^{-1})				CO_2 efflux (Tg C yr^{-1})		
	1980s	2010s	Change (%)	1st and 2nd orders change (%)*	1980s	2010s	Change (%)
Great Pearl	582.5±893.3	212.4±199.9	-63.5	-43.1	23.8±12.7	12.2±4.6	-48.7
Yangtze	599.8±828	375.3±576.3	-37.4	-17.9	46±24.4	29.9±15.3	-35
Huang-Huai-Hai	658.1±1008	412.6±424.1	-37.3	-45.2	15.7±8	9.2±3.8	-41.4
NE China	445.5±456.1	316.6±635.3	-28.9	-5.1	16.0±5.4	11.0±6.1	-31.3
NW China	894±1614.3	370.6±494	-58.5	-42.4	11.2±8.5	4.9±2.3	-56.3
Tibetan Plateau	554.7±596.7	476.8±780.2	-14.0	9.6	15.8±7.4	18.6±7.9	17.7
Total/Mean	629.6±976.2	347.9±530.3	-44.7	-33.4	128.6±31.3	85.8±19.4	-33.2

* Change of the annual F_{CO2} in the first- and second-order streams between the 1980s and 2010s.

For lakes, the CO_2 efflux declined in the Yangtze and Huang-Huai-Hai regions while increased in the high-latitude NE China and NW China regions and the high-altitude Tibetan Plateau region (Table 6.2). These diverse trends were largely consistent with the surface area changes of lakes (Table 4.11). Expanding lakes coupled with enhanced lateral carbon inputs from thawing glaciers and permafrost contributed to the elevated fluxes[78, 79, 84]. The largest increases were observed in the Tibetan Plateau and NW China regions, where mobilization of soil organic carbon from permafrost soils is predicted to increase with rising temperature in future fluxes[78, 85]. The F_{CO2} values of lakes and reservoirs during the two time periods generally showed an increasing trend in these two regions from the 1980s to the 2010s (Table 6.3).

Continuous construction of dams across China has resulted in increased CO_2 effluxes from reservoirs in five of the six regions (Table 6.2), which agreed well with the surface area increase as shown in Table 4.11. To some extent, the increased efflux reflected the spatial distribution of the newly built dams in the past three decades. The increase in the total CO_2 efflux from reservoirs between the two periods (72.3%; Table 6.2) is approximately equivalent to the surface water change (73.4%; Table 4.11).

Our efflux estimate for lakes and reservoirs was lower than Li et al (2018)'s recent estimate of 25.2 Tg C per year[86]. The primary difference lied in the F_{CO2}. Their F_{CO2} of 37.3 mmol m^{-2} d^{-1} for lakes and 44.4 mmol m^{-2} d^{-1} for reservoirs is up to one order of magnitude higher than those measured under the same climatic conditions in Europe and North America[87, 88, 89, 90]. Furthermore, our *in situ* measurements using floating chambers under different climates (see data

information in Section 10) also suggest that our F_{CO2} results (mean: 27.0±65.8 mmol m^{-2} d^{-1}) are more appropriate.

The total CO_2 efflux from Chinese inland waters in the 1980s and 2010s was 138±31 Tg C yr^{-1} and 98±19 Tg C yr^{-1}, respectively, indicating a net reduction of 29%. While the CO_2 efflux from lakes showed a slight increase (~15%), it is worth noting that conversion of free-flowing rivers to reservoirs has significantly reduced CO_2 emissions from streams and rivers across China (see Section 6.3 below for more discussion). If placed in the context of recent estimates of CO_2 evasion from global inland waters, the degassing of CO_2 from Chinese inland waters in the 2010s represents 5.1–8% of the global total[14]. Moreover, our flux estimates of CO_2 emissions from Chinese streams and rivers are comparable to that estimated for the streams and rivers in the United States (i.e., 97 Tg C yr^{-1})[20], indicating that both countries with similar land surface size have comparable CO_2 evasion.

Table 6.2 Change of CO_2 emissions from lakes and reservoirs between the 1980s and 2010s.

Region	Lakes (Tg C yr^{-1})			Reservoirs (Tg C yr^{-1})		
	1980s	2010s	Change (%)	1980s	2010s	Change (%)
Great Pearl	0.2±0.1	0.1±0.1	-50	0.3±0.1	0.8±0.5	166.7
Yangtze	2.1±1.1	1.1±0.4	-47.6	0.7±0.3	1.0±0.3	42.9
Huang-Huai-Hai	0.9±0.2	0.8±0.4	-11.1	0.5±0.1	0.8±0.4	60
NE China	1.3±0.2	1.4±0.3	7.7	0.5±0.1	0.6±0.2	20
NW China	0.7±0.2	1.2±0.5	71.4	0.1±0.02	0.4±0.1	300
Tibetan Plateau	2.1±1	3.8±1.1	81	0.03±0.01	0.07±0.02	133.3
Total/Mean	7.3±1.5	8.4±1.4	15.1	2.1±0.3	3.7±0.7	72.3

Table 6.3 Annual areal CO_2 emission (F_{CO2}) from lakes and reservoirs in the 1980s and 2010s.

Region	1980s: F_{CO2} (mmol m^{-2} d^{-1})			2010s: F_{CO2} (mmol m^{-2} d^{-1})		
	<10 km^2	10–50 km^2	>50 km^2	<10 km^2	10–50 km^2	>50 km^2
Great Pearl	36.7±24.9	25.2±24.0	21.7±11.1	61.3±47.6	18.9±24.2	7.6±10.0
Yangtze	44.4±32.9	41.7±23.7	24.2±29.7	20.8±44.4	43.4±110.4	13.3±20.0
Huang-Huai-Hai	34.7±15.3	30.5±13.9	35.2±18.3	42.3±63.7	45.4±47.3	22.2±16.1
NE China	11.2±7.6	42.7±29.3	30.8±19.7	42.7±60.9	21.2±19.1	25.1±6.4
NW China	27.5±2.5	36.8±15.0	34.4±17.6	80.5±43.2	48.4±32.8	40.1±29.4
Tibetan Plateau	14.6±15.0	15.6±16.7	10.9±16.0	71.4±72.4	9.2±18.2	13.7±29.4
Sub mean	26.2±22.1	25.9±20.1	18.7±13.5	36.7±62.8	33.3±82.2	14±21.6
Mean		23.3±21.4			27.0±65.8	

In order to explore the relative importance of each region in the total efflux, we calculated the percentage of efflux estimate for each region that accounts for the total flux (Figure 6.2). For streams and rivers, the largest change occurred in the Tibetan Plateau region where the expanding stream network and increasing F_{CO2} collectively contributed to a 10% increase. In comparison, all the other regions except the NE China generally showed a decreasing percentage in the 2010s, especially for the Greater Pearl and the NW China regions. River conversion to reservoirs was likely the primary reason, in particular for the NW China region where the surface area of reservoirs has increased by 277% (Table 4.11 and Figure 6.2).

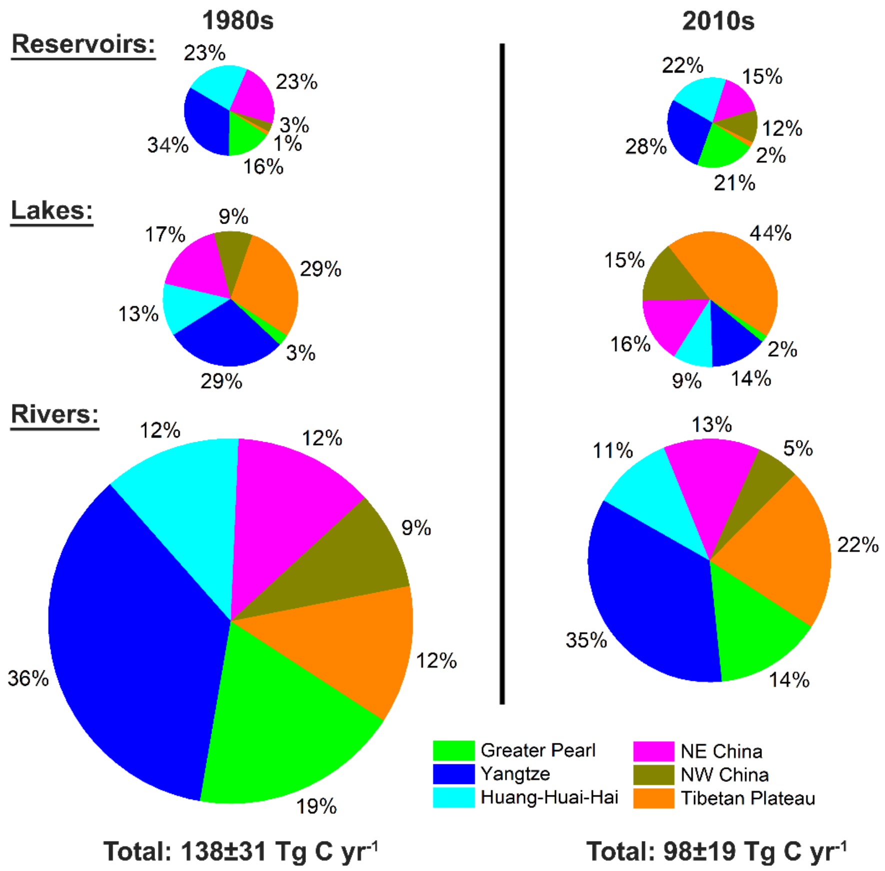

Figure 6.2 Comparison of CO_2 emissions across the six regions between the 1980s and 2010s. The sizes of the pie charts are approximately proportional to the average CO_2 evasion fluxes.

Comparison of the flux estimates between the two periods showed that the largest increases occurred in the Tibetan Plateau and NW China regions (Figure 6.2), which together represented 59% of the total efflux from lakes in the 2010s. In comparison, the percentages for the regions in eastern China have declined. These spatial variations are in line with the contrasting surface area changes – expanding lakes in western China whereas shrinking lakes in eastern China as outlined above. Evasion of CO_2 from reservoirs was predominated by the three large river-associated regions, namely the Greater Pearl (Pearl River), Yangtze (Yangtze River) and the Huang-Huai-Hai (Yellow River) regions. CO_2 effluxes from the three regions together accounted for 73% of the total in the 1980s and 71% of the total in the 2010s (Figure 6.2). This reflected the spatial distribution of hydropower development and the high human needs for water in agriculture, industry and domestic use. Approximately 86% of the population in China lives in these three regions[91].

The relative importance of the three inland water types in emitting CO_2 in the 1980s and 2010s was compared in the main text (Figure 3). Streams and rivers played a dominant role in both time periods, accounting for 93% and 88% of the total flux in the 1980s and 2010s, respectively. In comparison, the lentic systems played a minor role. Nevertheless, it is important to note that the importance of reservoirs has significantly increased over the three decades between the two time periods (Figure 3 in the text). The importance of reservoirs is predicted to continue to increase in

the coming decades because numerous new dams are currently under construction (see Section 6.5 for further discussion). Even so, we feel that streams and rivers will remain the dominant component in determining the magnitude of CO_2 emissions from Chinese inland waters.

6.3 River CO_2 efflux by Strahler order

In order to assess the importance of different Strahler order streams in total CO_2 efflux from rivers, we calculated the relative contribution of each Strahler order to the total efflux estimate (Figure 3 in the text). The streams were classified into three categories, namely headwater streams (1st and 2nd Strahler orders), intermediate streams (3rd–5th Strahler orders) and large streams (6th–8th Strahler orders). In general, headwater streams are characterized by high areal CO_2 emissions due to their strong turbulence at the water-air interface and high lateral carbon inputs from terrestrial ecosystems[40, 92, 93, 94]. Generally, headwater streams play a disproportionately important role in estimating total CO_2 efflux from stream networks. Wallin et al (2013)[93] discovered that first- and second-order streams in the Krycklan catchment (Sweden) are responsible for 72% of the total CO_2 evasion from the stream network, although they account for only 53% of the total stream surface area. Similarly, Marescaux et al (2018)[95] recently found that CO_2 evasion from headwater streams (1st and 2nd Strahler orders) in the Seine watershed in France account for 44% of the entire vertical flux of CO_2 from the stream network.

For the entire stream network across China, our flux estimation suggests that headwater streams were responsible for 55% of the total riverine CO_2 efflux in the 1980s (Figure 3 in the text). The percentage is even higher in the 2010s. This is because intermediate and large rivers have been progressively dammed and converted to reservoirs during the recent three decades. In contrast, the headwater streams comprised 34–38% of the total stream surface area across China (see Tables 4.1, 4.2, 4.6 and 4.7). The intermediate rivers were responsible for 33% and 25% of the total flux of CO_2 evasion in the 1980s and 2010s, respectively, and the remaining 12% in the 1980s and 14% in the 2010s was evaded from large river waters (Figure 3 in the text). In comparison, the intermediate and large river waters comprised 35–36% and 26–31% of the total stream surface area, respectively. This suggests that the decreasing F_{CO2} across the stream network has significantly reduced the relative importance of higher-order streams in the total flux of CO_2 evasion[96, 97, 98].

6.4 Potential drivers for the decreasing CO_2 evasion

The magnitude of CO_2 emissions from inland waters is controlled by human activity and natural factors[77, 99]. Major human activities in China include massive conversion of free-flowing rivers to reservoirs and widespread implementation of ecological restoration programs[80, 100]. Natural factors such as climate warming would change terrestrial carbon inputs into inland waters and surface water area, both of which may affect the CO_2 emission flux. In this section, we examined the potential drivers for the decreasing CO_2 emission fluxes and then conducted a quantitative evaluation of these drivers in responsible for the flux changes.

6.4.1 Human factors

We found that land cover and land use changes affected regional CO_2 emission dynamics. The Chinese government has implemented several national ecological restoration programs since the early 1980s to restore its degraded ecosystems[101]. For example, the ambitious Grain-for-Green project was initiated in 1999 to convert farmland that is susceptible to soil erosion to forest and

grassland[102]. To disentangle the potential impacts of forest management on CO_2 emissions across the six regions, we calculated the fractional forest cover change between the 1980s and 2010s (Figure 6.3a). Significant forest cover increases were observed in the previously agriculture-dominated eastern China, such as the Greater Pearl, Yangtze and Huang-Huai-Hai regions (Figure 1.1), due to widespread implementation of vegetation restoration programs[80, 81]. In contrast, the forest cover increase in the other three regions was much smaller and the NW China region even showed a decreased forest cover (-0.7%). If NW China was excluded from the analysis, there was a statistically significant correlation between forest cover changes and F_{CO2} changes across China (Figure 6.3b). The F_{CO2} change was found to decrease as a function of forest cover, showing an average decrease of 17.4 mmol m^{-2} d^{-1} with a 1% increase in forest cover. For the five regions showing increasing forest cover between the two periods, our analysis suggests that forest cover changes can explain 83% of variability of the F_{CO2} changes (Figure 6.3b). We feel that the reduced lateral carbon from forested watersheds and altered dissolved organic matter (DOM) composition are probably the major reasons. The DOM derived from forested watersheds with less anthropogenic modification tends to be less labile and accessible to microbial community than that from agricultural watersheds[103, 104]. The steadily increasing forest cover across most of China due to the implementation of ecological restoration programs is therefore a potential driver for the decreased CO_2 evasion flux from streams and rivers in the 2010s compared to the 1980s (Table 6.1 and Figure 6.2).

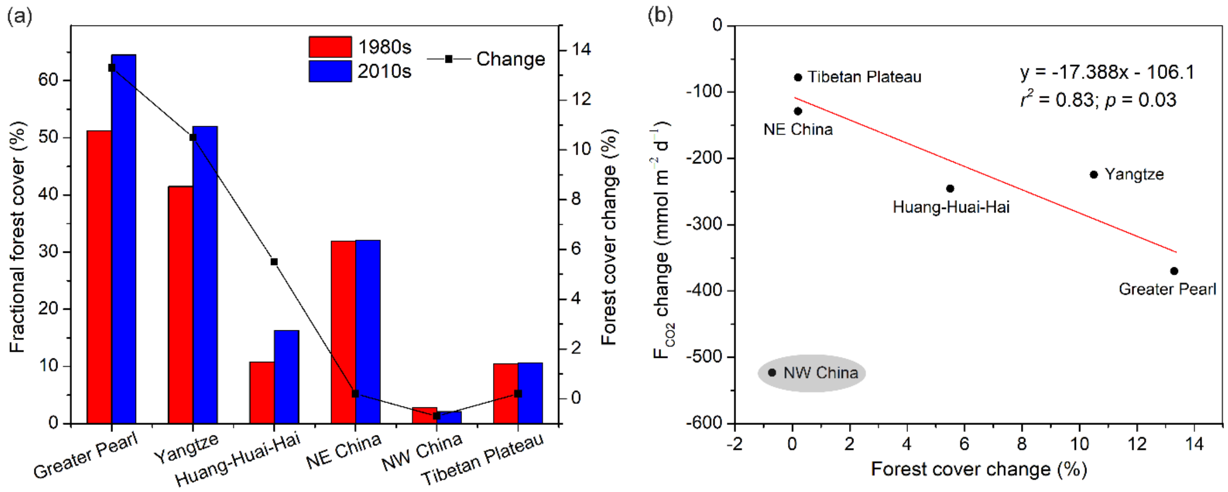

Figure 6.3 Forest cover dynamics across the six regions of China (a) and relationship between fractional forest cover change and F_{CO2} change across the six regions between the 1980s and 2010s. Note that the NW China region in (b) was excluded from the linear regression analysis.

As an important land use type in China, the fractional cropland cover was found to be statistically related to stream water pCO_2 (Figure 6.4). The Huang-Huai-Hai region with the largest cropland cover showed the highest stream water pCO_2 level. We reason that there are two governing factors responsible for the positive correlation between pCO_2 and cropland cover. Agricultural activities, such as tilling and ploughing, are associated with strong soil detachment and movement and thus high export of lateral carbon into streams and rivers[80, 98]. Furthermore, agricultural land use generally causes elevated delivery of nutrients to rivers compared with forests. Excessive application of chemical fertilizers in China started to surge from the early 1980s and China now is the largest consumer of synthetic fertilizers in the world as mentioned above. As a populous country with 60% of its population being farmers[105], agricultural land use

in China is associated with a much higher population density than other land use types (except urban areas) and huge amounts of largely untreated wastewater inputs into aquatic systems. As a result, Chinese inland waters have been suffering from pervasive pollution due to increased nutrient discharge from cropland, domestic and even industrial sources[2]. The high availability of nutrients in agriculture-affected streams may facilitate the in-stream degradation of organic carbon, leading to high dissolved CO_2 concentrations in agricultural streams[106, 107]. On the other hand, the cropland area in China has been reduced over the study period, especially in the recent 20 years due largely to unprecedented urbanization and the nationwide implementation of vegetation restoration programs[108, 109, 110]. Along with the decreasing F_{CO2} with increasing forest cover in Figure 6.3b, this positive relationship between pCO_2 and fractional cropland cover further suggests that the conversion of cropland land to forest (and perhaps also grassland) is partially responsible for the reduced CO_2 emissions.

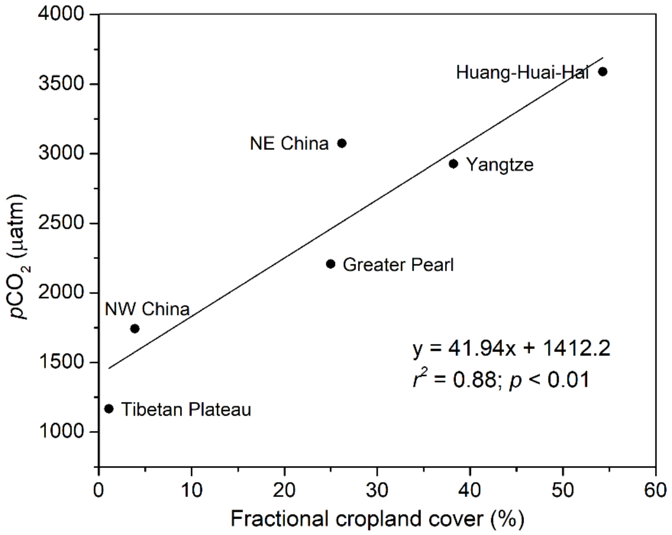

Figure 6.4 Regression analysis of stream water pCO_2 response to fractional cover of cropland across the six regions.

Damming of rivers will change the original physical and biogeochemical properties of primitive rivers[26, 111, 112]. Greatly reduced flow velocity within reservoirs created a favorable environment for aquatic plant growth. Reservoir waters are typically characterized by higher primary productivity as compared with flowing rivers. Therefore, construction of dams on rivers will be able to change the carbon export dynamics within a river system. The magnitude and direction of CO_2 exchange across the water-air interface would be changed, depending on a suite of biotic and abiotic factors such as productivity, trophic status, land use, geology and catchment morphometry[113, 114, 115]. This change in carbon export can be reflected from two aspects. First, the surface area of running waters will be reduced whereas the surface area of stagnant waters will expand accordingly. In most cases, the expanded reservoir surface area will exceed the reduced river surface area because flat floodplains would be submerged with increasing water level after dam impoundment. Second, the areal CO_2 emission (F_{CO2}) will also be changed. Mineralization of organic matter stored in the drawdown zones tends to produce high greenhouse gas emissions[116, 117]. Recent studies suggest that reservoir age and latitude are important predictors of reservoir CO_2 and methane (CH_4) evasion with elevated greenhouse gas emissions from young (e.g., less than 10 years) reservoirs relative to the old ones[112, 115]. In addition, trophic status and

associated primary productivity also play an important role in determining reservoir CO_2 and CH_4 dynamics. Both surface water pCO_2 and CO_2 evasion would decrease with increasing eutrophication and primary production[118, 119].

As outlined in Section 4.3, numerous dams were constructed in China over the past decades for purposes of water supply, irrigation, hydropower, flood control and navigation. Currently China hosts more than half of global large reservoirs and is projected to remain the global leader in hydropower dam construction, producing approximately 25% of global future hydropower development[120, 121]. Continuous conversion of flowing rivers to stagnant waters in reservoirs has greatly expanded the surface area of reservoirs in all the six regions across China (Table 4.11). Moreover, widespread eutrophication resulting from high nutrient loading from agriculture and urbanization has enhanced photosynthesis of aquatic plants in these lentic ecosystems. As a result, the F_{CO2} values in reservoir waters were significantly lower than that observed in streams and rivers and the magnitude of CO_2 evasion from reservoirs was greatly lowered. Pacheco et al (2014)[122] concluded that increasing primary production could shift lentic ecosystems from CO_2 sources to sinks. Our earlier study[26] in the middle Yellow River (in the Huang-Huai-Hai region) also reported a substantially lower F_{CO2} in reservoirs than in rivers and the F_{CO2} in reservoirs was seasonally negative (e.g., in spring and summer) indicating a carbon uptake from the atmosphere. In comparison, flowing streams and rivers were consistently strong CO_2 sources with a much higher F_{CO2} (ref [26]). Although the total water surface area has increased after dam impoundment that converts rivers to reservoirs, the overall CO_2 evasion has been greatly reduced. If rivers and reservoirs were considered together, the total CO_2 efflux of the two water bodies has declined from 130.7 to 89.5 Tg C yr^{-1} over the past 30 years, corresponding to a reduction of 32% relative to the reference period (the 1980s). Therefore, the massive conversion of free-flowing rivers to reservoirs through dam impoundment has effectively reduced the total flux of CO_2 evasion from Chinese river systems on the 30-year-long timescale. This great reduction was simultaneously accompanied by increasing trophic status in most reservoirs across China[123, 124, 125].

However, although CO_2 emissions have been greatly reduced after the conversion, it is also important to understand that lentic ecosystems may be substantial sources of CH_4. A growing body of work indicates that increasing trophic status tends to create favorable environments for CH_4 production and evasion[44, 98, 113]. Field measurements of CH_4 evasion based on case studies of reservoirs in China indicate that its reservoirs are characterized by considerably high CH_4 evasion fluxes and function as hotspots of CH_4 emission[117, 126, 127, 128]. It is worthy of estimating the simultaneous CH_4 evasion from reservoirs to quantify their total carbon emission flux, which warrants future research. This is discussed in the main text.

6.4.2 Natural factors

Outgassing of CO_2 from inland waters is also affected by climate as discussed above. There is a statistically significant relationship between annual precipitation and regional CO_2 emission flux in the 1980s and 2010s (Figures 6.5a and 6.5b). This can be explained by two mechanisms. First, because a higher annual precipitation corresponds to a higher flow discharge and thus larger water surface exposure to the atmosphere as shown in Figure 4.6, this will likely increase the flux of CO_2 evasion. Furthermore, a catchment with a higher precipitation is generally characterized by a higher terrestrial primary productivity[1, 20]. When coupled with higher flushing and delivery of lateral carbon by the higher flow, this will lead to stronger CO_2 evasion.

Consequently, the two regions in southern China (Greater Pearl and Yangtze) with higher precipitation and primary production showed substantially higher CO_2 evasion rates than those having lower precipitation. As implied by the high r^2, annual precipitation can explain a significant portion (>80%) of the variance in the total CO_2 emission flux in each region. In comparison, the relationships between temperature and area-weighted CO_2 evasion in the 1980s and 2010s were shown in Figures 6.5c and 6.5d, respectively. Although the statistical significance of the correlations was somewhat weaker, the positive responses suggest that increasing temperature will lead to higher CO_2 evasion. Therefore, it is reasonable to assume that climate warming over time would change surface water pCO_2 and CO_2 emission rates. The potential impact of climate change is particularly evident in the Tibetan Plateau region which is most sensitive to climate warming among the six regions examined in this study.

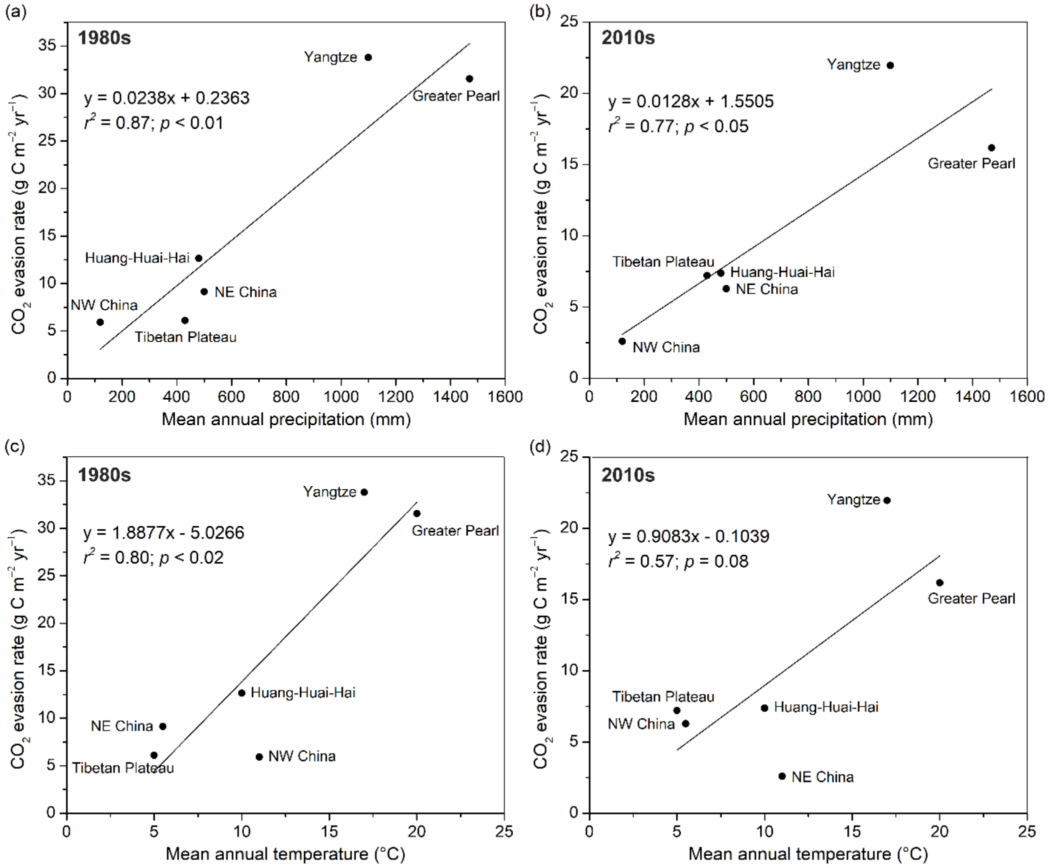

Figure 6.5 Relationship between climate variables (precipitation and temperature) and land surface area-averaged CO_2 evasion rate in the 1980s and 2010s. The long-term mean precipitation and temperature during the period 1980s–2010s were used here for the analysis.

6.4.3 Attribution analysis of human and natural factors

We determined the impacts of temporal changes in the areal CO_2 emission rate (ΔF_{CO2}) and water surface area (ΔSA) on the changes in the total CO_2 emission flux for both flowing rivers and standing waters (lakes and reservoirs) on the regional basis. This was done through a linear regression analysis that revealed the explanatory power of the two factors in driving the variability of the CO_2 emission fluxes[129]. The results for streams and rivers are presented in Table 6.4 and the results for lakes and reservoirs are presented in Table 6.5. For streams and rivers, ΔF_{CO2} is the primary driver for the changing CO_2 emission flux in all the six regions,

which can explain 58–97% of the variance of the CO_2 emission flux changes ($p < 0.05$). This is particularly true for the Greater Pearl, Yangtze and Tibetan Plateau regions. For all regions except the Tibetan Plateau, this largely reveals the impacts of human activities (e.g., river regulation via the conversion of flowing rivers to reservoirs and widespread implementation of ecological restoration programs) in reducing stream water pCO_2. Furthermore, the reduced water velocities due to damming will also result in decreased near-surface turbulence, thereby reducing the gas transfer velocity and subsequently the F_{CO2} (refs [26, 98]). In particular, for the extremely dry NW China region with degraded forest cover (2.1% of the regional land surface in the 2010s), the large decrease in F_{CO2} (Table 6.1) was more likely due to river impoundment. The NW China region showed the highest increase in reservoir surface area (277%; Table 4.11). River regulation by cascade dams tends to slow water velocities and promote primary production by alleviating light limitation through sedimentation[87, 98], which ultimately decreases dissolved CO_2 and therefore its evasion rate. In comparison, for the Tibetan Plateau with relatively low human impacts, climate warming was likely the major driver for its F_{CO2} changes. This is particularly possible for its headwater streams where the F_{CO2} has surprisingly increased by 9.6% (Table 6.1), which is different from all the other regions.

However, it is worth noting that water surface changes are of equal importance in explaining the variance of the CO_2 emission changes in the NW China and Huang-Huai-Hai regions, both of which are characterized by an arid climate with low precipitation. The ΔSA can explain 74% and 77% of the variance of the vertical CO_2 flux changes in the Huang-Huai-Hai and NW China regions, respectively. These values are comparable to that of the ΔF_{CO2} (Table 6.4). This is consistent with the surface area changes in these two regions that showed the largest temporal changes among the six regions (Table 4.11). Massive dam construction and climate warming in these two regions were likely the primary factors for the decreased water surface. We feel that, in the wet regions (i.e., Greater Pearl, Yangtze and NE China), F_{CO2} changes were the primary driver for the total CO_2 efflux changes whereas water surface area changes played a secondary role. But in the dry regions (i.e., Huang-Huai-Hai and NW China), both F_{CO2} and water surface area changes were equally important in determining the total CO_2 efflux changes.

Table 6.4 Linear relationships between total CO_2 efflux change (Tg C) and areal CO_2 emission flux change (ΔF_{CO2}, mmol m^{-2} d^{-1}) and between total CO_2 efflux change (Tg C) and water surface area change (ΔSA, km^2) during the 1980s and 2010s in streams and rivers. The bold *p* values indicate significance at α=0.05.

Region	Item	Linear equation	r^2	*p*
Greater Pearl	ΔF_{CO2}	y = 0.0066x + 0.2256	0.97	**0.00005**
	ΔSA	y = -0.0045x - 2.2093	0.21	0.307
Yangtze	ΔF_{CO2}	y = 0.0074x - 0.751	0.90	**0.0004**
	ΔSA	y = -0.0016x - 2.5315	0.15	0.345
Huang-Huai-Hai	ΔF_{CO2}	y = 0.0064x + 0.0721	0.84	**0.0038**
	ΔSA	y = 0.0074x + 0.1381	0.77	**0.0089**
NE China	ΔF_{CO2}	y = 0.0105x + 0.252	0.58	**0.0455**
	ΔSA	y = 0.0032x - 0.2734	0.17	0.359
NW China	ΔF_{CO2}	y = 0.0035x + 0.2849	0.83	**0.0118**
	ΔSA	y = 0.0084x - 0.0934	0.74	**0.0286**
Tibetan Plateau	ΔF_{CO2}	y = 0.0094x + 0.4023	0.97	**0.0003**
	ΔSA	y = 0.0055x + 0.0676	0.17	0.409

We also plotted the overall impact of ΔF_{CO2} and water surface changes on the total CO_2 efflux (Figure 6.6). If the six regions were considered together, the F_{CO2} changes resulting from changed pCO_2 and gas transfer velocity have a higher explanatory power in explaining the variability in the total efflux changes (69%, p <0.0001). In comparison, water surface area changes showed a very weak explanatory power, largely because water surface changes are only important in the Huang-Hai-Hai and NW China regions in explaining the variability in the total efflux changes (Table 6.4).

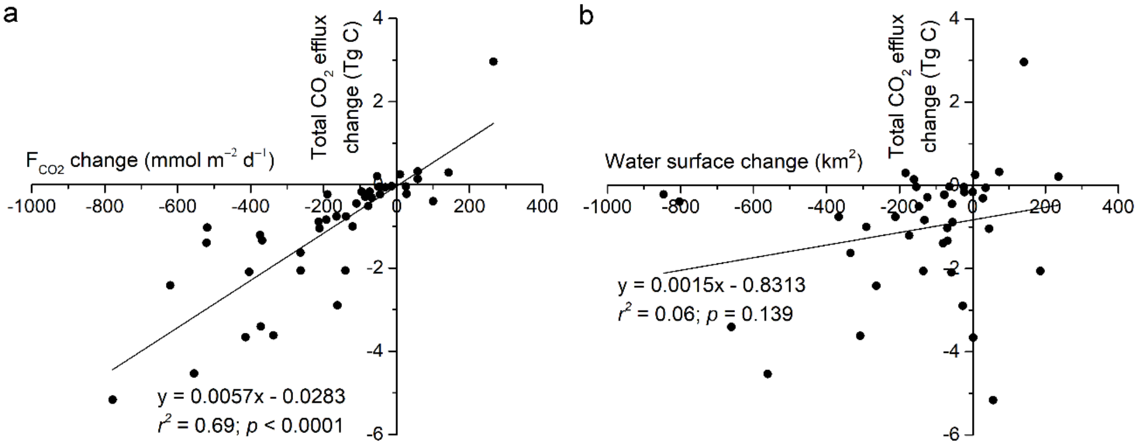

Figure 6.6 Relationship between total CO_2 efflux change and (a) F_{CO2} change and (b) water surface change for streams and rivers in China.

For natural lakes, F_{CO2} changes remained a dominant factor in driving the variability in the total CO_2 efflux across China (national mean: 67%, Tables 6.5 and 6.6). This was likely caused by the strong human activities in reducing pCO_2. Chinese lakes (and also reservoirs) have been suffering from severe eutrophication due to high nutrient loading from agriculture and urbanization over the past decades[125, 130]. China is now the largest consumer of synthetic fertilizers, consuming nearly one third of the world's nitrogen fertilizers and one fourth of the world's phosphate fertilizers[2, 131]. Yu et al (2019)[2] reported that nitrogen discharge into Chinese inland water bodies started to surge from the early 1980s (soon after the initiation of the reform and opening-up policy) and the critical surface water quality standard (1 milligrams of nitrogen per liter) was exceeded in most provinces in China by the mid-1980s until now. In addition to extensive nutrient loading from agriculture, it is also important to understand the impact of wastewater discharge from expanding cities. Domestic sewage discharge has become a major source of water pollution due to rapid urban expansion since the early 1980s[132, 133]. These human activities have not only negatively impacted water as an important resource, but also reduced CO_2 evasion. Recent *in situ* measurements of CO_2 degassing from lakes in the Yangtze region indicate that they typically acted as small CO_2 sources and even CO_2 sinks owing to high primary productivity caused by algal blooms[134, 135, 136]. Intensive eutrophication of lake waters in eastern China, including the Greater Pearl, Yangtze, Huang-Huai-Hai and the NE China regions, that coincided with widespread use of fertilizers in agriculture is therefore an important reason for the reduced flux of CO_2 evasion. In comparison, water surface area changes can explain 29% of the variability in the total CO_2 efflux, but they are an important driver in south China (i.e., the Yangtze and Greater Pearl regions, Table 6.5). This largely reveals the impact of widespread land reclamation for agriculture as discussed in Section 4.3.

Like lakes, the areal CO_2 emission rate is the primary driver for the increased reservoir CO_2 effluxes in most of the regions (Table 6.5). However, surface area changes are also important in driving the temporal changes in the vertical CO_2 flux, especially in the Greater Pearl, NE China and NW China regions. The surface area changes in these regions can explain 69–99% of the variability of the CO_2 efflux changes. Although the overall reservoir F_{CO2} in the Yangtze and NE China region has declined by 29.4% and 7.6%, respectively, between the two periods (Figure 4 in the main text), the F_{CO2} in the <10 km^2 size class in both regions has greatly increased, which has offset the reduced CO_2 emission fluxes from the other two size classes (10–50 and >50 km^2). We found the reservoir F_{CO2} generally exhibited a large decrease (>45%) in large reservoirs in eastern China (e.g., the 10–50 and >50 km^2 classes in the Greater Pearl, Yangtze and NE China regions and the >50 km^2 size class in the Huang-Huai-Hai region). This largely reflected the human impacts, such as eutrophication resulting from agriculture and domestic sewage discharge, as widely observed in Chinese lakes[125, 135]. In contrast, the high explanatory power of F_{CO2} in explaining the variability of the reservoir CO_2 efflux changes in the Tibetan Plateau and NW China regions (Table 6.5) was more related to climate change-induced carbon and CO_2 inputs into reservoir waters. Consequently, the F_{CO2} changes in both regions were comparable (Figure 4c in the main text).

For all reservoirs, surface area increases explained 50% of the variability of the increased CO_2 effluxes whereas ΔF_{CO2} played a minor role due largely to human impacts like eutrophication (Table 6.6). This is in accordance with the consistent increase in reservoir water surface in all the six regions (Table 4.11). Nevertheless, if we consider lakes and reservoir together, changes in F_{CO2} become more important in driving the total CO_2 efflux changes as suggested by the higher r^2 and slope, consistent with the lake CO_2 efflux changes that depends more on F_{CO2} (Table 6.6). This is not surprising because lakes dominated the CO_2 emissions from lentic ecosystems (lakes+reservoirs) as shown in Table 6.2.

Table 6.5 Linear relationships between total CO_2 efflux change (Tg C) and area-weighted CO_2 emission flux change (ΔF_{CO2}) and between total CO_2 efflux change (Tg C) and water surface area change (ΔSA) during the 1980s and 2010s in lakes and reservoirs.

Region	Item	Lakes		Reservoirs	
		Linear equation	r^2	Linear equation	r^2
Greater Pearl	ΔF_{CO2}	y = 0.0005x - 0.0195	0.93	y = 0.002x + 0.0755	0.99
	ΔSA	y = 0.0003x + 0.0198	0.77	y = 0.0004x - 0.1655	0.98
Yangtze	ΔF_{CO2}	y = 0.0075x - 0.0567	0.41	y = 0.0041x + 0.2003	0.95
	ΔSA	y = 0.001x + 0.2315	0.82	y = 0.00007x - 0.0165	0.48
Huang-Huai-Hai	ΔF_{CO2}	y = 0.002x - 0.105	0.72	y = 0.0012x + 0.0051	0.88
	ΔSA	y = 0.0007x - 0.0383	0.90	y = 0.0002x + 0.0156	0.92
NE China	ΔF_{CO2}	y = 0.0018x - 0.1193	0.86	y = 0.0002x - 0.0767	0.87
	ΔSA	y = 0.0008x + 0.0543	0.25	y = 0.0003x - 0.0767	0.99
NW China	ΔF_{CO2}	y = 0.0015x + 0.0665	0.99	y = 0.0003x + 0.0676	0.86
	ΔSA	y = 0.00007x + 0.1692	0.01	y = 0.0003x + 0.007	0.69
Tibetan Plateau	ΔF_{CO2}	y = 0.0026x + 0.2373	0.83	y = 0.00001x + 0.0031	0.98
	ΔSA	y = -0.00003x + 0.6077	0.01	y = 0.00007x + 0.0103	0.24

Table 6.6 Linear relationships between total CO_2 efflux change and areal CO_2 emission flux change (ΔF_{CO2}) and between total CO_2 efflux change and water surface area change (ΔSA) during the 1980s and 2010s in lakes and reservoirs in China. The bold *p* values indicate significance at α=0.05.

Water body	Item	Linear equation	r^2	p
Lakes	ΔF_{CO2}	y = 0.0025x - 0.054	0.67	**0.0001**
	ΔSA	y = 0.0002x + 0.0193	0.29	**0.0199**
Reservoirs	ΔF_{CO2}	y = 0.00004x + 0.0708	0.03	0.542
	ΔSA	y = 0.0001x + 0.0007	0.50	**0.0009**
Lakes+Reservoirs	ΔF_{CO2}	y = 0.0169x + 0.0447	0.68	**0.0001**
	ΔSA	y = 0.0002x - 0.0157	0.23	**0.0449**

7. Robustness test of temporal changes in F_{CO2} between 1980s and 2010s

To test the robustness of the comparative analysis of the total fluxes, especially for streams and rivers which dominated the total evasion (Figure 6.2), we compared the F_{CO2} results obtained for the same sampling locations over the two periods. This comparison enabled us to remove the potential biases introduced by different sampling locations and therefore the obtained differences reveal only the temporal changes. There are 91 stream sampling sites where F_{CO2} is available in both time periods (Figure 7.1). These sampling sites account for 7% and 13.8% of the sample size of the 1980s and 2010s, respectively. These stream sites include all the 8 Strahler orders and are scattered in the six defined regions across China. The changes of seasonal and annual mean F_{CO2} between the two periods were shown in Table 7.1. Of the 91 sampling sites, 72% of them showed a decreasing F_{CO2} over the two periods. The reductions in F_{CO2} in the six regions were comparable to the observed changes from the entire dataset for each region as shown in Table 6.1. In addition, the national annual mean F_{CO2} decease between the two periods (40.1%) is also largely consistent with the observed decrease for the whole dataset (44.7%). Along with the discussion above, this suggests that decreasing F_{CO2} is widespread across China.

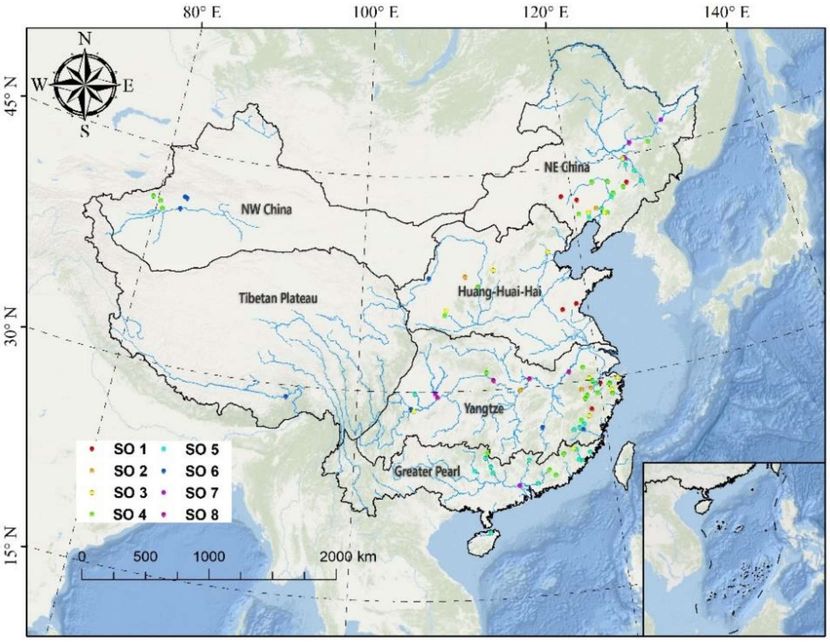

Figure 7.1 Location map of the 91 sampling sites where CO_2 evasion rate is available in the 1980s and 2010s.

Table 7.1 Change of mean F_{CO2} for the 91 sampling locations between the 1980s and 2010s.

Region	No. of sites	Dry season F_{CO2} change (%)	Wet season F_{CO2} change (%)	Annual mean F_{CO2} change (%)
Greater Pearl	17	-58.9	-58.2	-58.6
Yangtze	34	-22.2	-47.1	-32.6
Huang-Huai-Hai	9	-31.1	-38.6	-33.6
NE China	24	-34.1	-33.1	-33.9
NW China	6	-46.4	-27.3	-41.6
Tibetan Plateau	1	-2.3	-22.5	-9.1
Total/Mean	91	-34.1	-44.0	-40.1

8. Uncertainties in CO_2 effluxes

Upscaling CO_2 effluxes from site-based measurements is subject to errors due to uncertainties in all the three factors (i.e., surface water pCO_2, k value and water surface area) in estimating regional fluxes. We estimated the propagated errors of the total CO_2 efflux by treating the errors on these three components as being statistically independent. This allows us to evaluate the propagation of uncertainty by considering the uncertainty of the individual components in quadrature[137]. The error on the pCO_2 resulted in an overall uncertainty of areal CO_2 effluxes of ±11%. While for the uncertainty on the k estimates, it was estimated to be ±7% for streams and rivers that was based on the errors from flow velocity and channel slope and it was ±5% for lakes and reservoirs that was based on wind speed. Consequently, this led to a propagated uncertainty of areal CO_2 effluxes of ±16% for streams and rivers, and ±13% for lakes and reservoirs that also considered the error in Figure 3.7. Lastly, because the water surface area of rivers/streams, lakes and reservoirs were separately estimated and the seasonal changes of stream and river water surface were already considered on the basis of ground measurements, the uncertainty associated with water surface area estimates was relatively small at ±8.1% by comparing with recent national-scale studies, high-resolution satellite images and statistical reports (see Section 4). Our field measurements of hydraulic geometry of streams and rivers over different climates, seasons and Strahler orders (Figure 2.4) also lend confidence to the surface area estimates.

We conducted a bootstrapping Monte Carlo simulation to evaluate errors associated with our estimates by considering the uncertainties of the individual components. The simulation was conducted for each season and Strahler order for streams and rivers in each region and for each season and size class for lakes and reservoirs in each region. Uncertainty associated with pCO_2 and the gas transfer velocity was determined by examining the variance (i.e., one standard deviation) of corresponding variables within each of the simulation unit (e.g., season and Strahler order for streams and rivers and season and size class for lakes and reservoirs). Uncertainty associated with regional water surface areas was assumed to be 8.1% of the water surface area in each region. These uncertainties were then propagated through the upscaling process to compute an estimate with uncertainties specified for each component. We performed the Monte Carlo simulation with 10,000 iterations to generate a distribution of the estimates. Errors associated with the estimates were finally determined by calculating the 5th and 95th percentiles as confidence intervals. All bootstrap analyses were conducted in the statistical language R[138].

Table 8.1 shows uncertainty estimates for each of the regions in the 1980s. The overall errors on total effluxes are in general within 9–26% of the raw estimates of the three inland water types. For the 2010s estimate, we instead used areal CO_2 evasion rate (F_{CO2}) and water surface area for

the simulation because surface water pCO_2 and k (or k_{600}) results were not reported for 73% of the measurements in the original datasets (see Section 2.2). Table 8.2 presents the comparison between the original CO_2 effluxes and the simulated CO_2 effluxes in the 2010s as well as the uncertainties. The simulated total efflux across all the six regions was very close to the original estimate presented in the text. Overall, while the efflux estimate bias for lakes was larger than that for streams/rivers and reservoirs, the discrepancy was still reasonable (i.e., 6.6% in the 1980s and 2.1% in the 2010s). The total bias between the original and simulated estimates was 9.3 and 2.1 Tg C yr^{-1} in the 1980s and 2010s, respectively, falling into the uncertainty range. Therefore, this simulation estimate provides confidence in our method, and it is for this reason that we continue to use the original efflux estimates based on the true dataset instead of the simulated totals. The integrated effluxes from the three inland water types and across the six regions were 91–200 Tg C yr^{-1} (5th and 95th confidence interval percentiles) in the 1980s and 66–136 Tg C yr^{-1} (5th and 95th confidence interval percentiles) in the 2010s.

Table 8.1 Monte Carlo simulation results for total CO_2 efflux from rivers/streams, lakes and reservoirs in the 1980s across China.

Region	Rivers (Tg C yr^{-1})				Lakes (Tg C yr^{-1})				Reservoirs (Tg C yr^{-1})			
	Original efflux	Simulated efflux	Simulated efflux 5th percentile	Simulated efflux 95th percentile	Original efflux	Simulated efflux	Simulated efflux 5th percentile	Simulated efflux 95th percentile	Original efflux	Simulated efflux	Simulated efflux 5th percentile	Simulated efflux 95th percentile
Great Pearl	23.8	16.9	4.8	46.3	0.2	0.17	0.1	0.3	0.3	0.25	0.1	0.4
Yangtze	46.0	39.5	7.3	87.4	2.1	2.2	0.2	4.0	0.7	0.73	0.3	1.2
Huang-Huai-Hai	15.7	15.0	3.1	29.1	0.9	1.0	0.6	1.3	0.5	0.53	0.3	0.6
NE China	16.0	17.0	7.4	25	1.3	1.7	1.0	1.5	0.5	0.6	0.4	0.6
NW China	11.2	12.5	-3.0	35.7	0.7	1.0	0.3	1.0	0.1	0.103	0.04	0.1
Tibetan Plateau	15.8	16.5	1.9	36.6	2.1	3.2	0.6	3.7	0.03	0.034	0.001	0.1
Total	128.6	117.4	81.3	191.0	7.3	9.2	4.6	9.8	2.1	2.3	1.5	2.5

Table 8.2 Monte Carlo simulation results for total CO_2 efflux from rivers/streams, lakes and reservoirs in the 2010s across China.

Region	Rivers (Tg C yr^{-1})				Lakes (Tg C yr^{-1})				Reservoirs (Tg C yr^{-1})			
	Original efflux	Simulated efflux	Simulated efflux 5th percentile	Simulated efflux 95th percentile	Original efflux	Simulated efflux	Simulated efflux 5th percentile	Simulated efflux 95th percentile	Original efflux	Simulated efflux	Simulated efflux 5th percentile	Simulated efflux 95th percentile
Great Pearl	12.2	10.63	5.0	19.4	0.1	0.15	0.01	0.3	0.8	0.76	-0.1	1.6
Yangtze	29.9	29.98	6.2	56.5	1.1	1.14	0.4	1.8	1.0	0.99	0.5	1.5
Huang-Huai-Hai	9.2	10.00	3.7	17.2	0.8	0.81	0.2	1.4	0.8	0.78	0.2	1.4
NE China	11	12.60	-4.4	30.2	1.4	1.32	0.8	1.9	0.6	0.55	0.3	0.8
NW China	4.9	5.72	1.5	11.1	1.2	1.24	0.5	2.0	0.4	0.43	0.2	0.6
Tibetan Plateau	18.6	18.96	6.4	32.4	3.8	3.78	2.0	5.6	0.07	0.07	0.04	0.1
Total	85.8	87.89	54.3	124.3	8.4	8.44	6.3	10.7	3.67	3.58	2.4	4.8

Other sources of uncertainty that are not straightforward to quantify may relate to the spatial representativeness of the sampling sites across the vast area of China. On the one hand, water chemistry data in the 1980s were comparatively lacking in the NE China and Tibetan Plateau regions (Figure 2.1). Considering the low temperature in both regions that has partly constrained organic matter decomposition and resulted in low pCO_2 and CO_2 effluxes[139, 140], the resulting error to CO_2 efflux is considered small. On the other hand, headwater streams can generally

contribute disproportionally to CO_2 efflux in steep, mountainous catchments[39, 41, 92, 94]. Our earlier study[26] in the Wuding River catchment in the middle Yellow River (in the Huang-Huai-Hai region) indicates that no significant difference in areal CO_2 efflux between headwater streams and downstream streams due to strong carbonate buffering. Even so, we paid special attention to regions with elevation difference exceeding 2000 m, especially the Greater Pearl, Yangtze and the Tibetan Plateau regions. These regions are characterized by steep topography and turbulent headwater streams.

In order to account for the impact of high-gradient streams in estimating CO_2 efflux, we calculated their *k* values from flow velocity and stream channel slope based on the models proposed by Raymond et al (2012)[36] and Ulseth et al (2019)[41]. Details are presented in Section 3.1. We calculated the CO_2 effluxes from streams and rivers in the 1980s and 2010s by using the *k* values derived separately from the two approaches while keeping the other variables (e.g., pCO_2 and water surface area) unchanged. The CO_2 efflux estimates based on the two approaches were presented in Table 8.3, and the comparison was illustrated in Figure 8.1. Although the Ulseth approach used a piecewise linear regression (Equations 2 and 3) to differentiate the *k* values in high-energy, mountain streams from that in low-energy streams, the calculated CO_2 effluxes by the two approaches are consistent with each other. The total efflux estimates by the two approaches varied by 5.1% in the 1980s and 0.8% in the 2010s (Table 8.3). The high discrepancies were observed in the NE China and NW China regions in the 1980s. This is likely because most of the hydrologic gauges in these regions are placed in low-gradient channels, whereas insufficient data from high-gradient streams have resulted in different *k* values by the two approaches (Figures 3.3 and 3.4).

Table 8.3 CO_2 effluxes from streams and rivers of the six regions in the 1980s and 2010s.

Region	1980s (Tg C yr^{-1})			2010s (Tg C yr^{-1})		
	Ulseth	Raymond	Change Relative to Raymond (%)	Ulseth	Raymond	Change Relative to Raymond (%)
Great Pearl	27.5	23.8	15.5	11.8	12.2	-3.3
Yangtze	46.8	46.0	1.7	32.6	29.9	9.0
Huang-Huai-Hai	15.3	15.7	-2.5	8.3	9.2	-9.8
NE China	12.9	16.0	-19.4	9.7	11.0	-11.8
NW China	14.4	11.2	28.6	5.2	4.9	6.1
Tibetan Plateau	18.2	15.8	15.2	18.9	18.6	1.6
Total	135.1	128.6	5.1	86.5	85.8	0.8

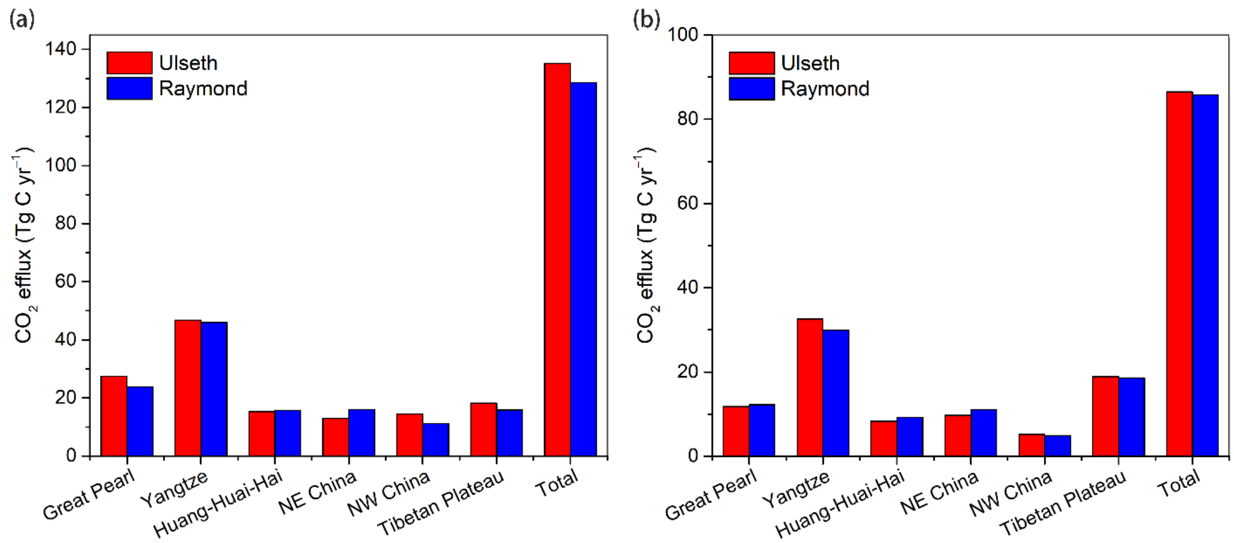

Figure 8.1 Comparison of CO_2 efflux of streams and rivers between the Ulseth and Raymond approaches in the 1980s (a) and 2010s (b). See the efflux results in Table 8.3.

Because measurements were generally conducted in higher-order streams, CO_2 emissions from turbulent streams draining mountains may have not been fully represented. This is particularly true for the hydrologic gauge-based water chemistry records in the 1980s. Mountain streams are characterized by high CO_2 effluxes due to their strong land-water connectivity and high gas transfer velocity[39, 41, 93, 141]. Horgby et al (2019)[39] recently estimated CO_2 emissions from global mountainous streams with strong turbulence. They computed a median areal CO_2 efflux of 3.5 kg C m^{-2} yr^{-1} (-0.5–23.5 kg C m^{-2} yr^{-1}; 5th and 95th confidence interval percentiles) for 23,343 mountainous streams. If the median areal CO_2 efflux of 3.5 kg C m^{-2} yr^{-1} (or 799.1 mmol m^{-2} d^{-1}) is used for the first- and second-order streams of all the six defined regions across China, the total CO_2 efflux from streams and rivers in the 1980s and 2010s will be 121.5 and 94.7 Tg C yr^{-1}, respectively. When compared with our estimates, these correspond to a difference of 5.5% for the 1980s estimate and 10.3% for the 2010s estimate. Clearly, the estimates for mountain streams (first- and second-order streams here) across China are within our uncertainty range.

We recognize that our surface area estimate of headwater streams is probably associated with errors due to the spatial clustering of hydrologic gauges towards higher-order streams. Narrow headwater streams (e.g., <1 m wide[39, 142]) may have not been completely included in our stream network delineation and surface area estimation. However, we feel that our surface area estimate of the stream network has largely reflected the overall water surface extent of Chinese streams and rivers. This can be seen from the percentage of water surface to the total land surface (i.e., 0.58–0.68% in the 1980s and 0.53–0.61% in the 2010s), consistent with recent estimate of percentage of global land surface covered by streams and rivers (i.e., 0.58±0.06%, see Allen and Pavelsky (2018)[143]). Particularly, the percentage of drainage area covered by streams and rivers in the Yangtze region (1.46% in the 1980s and 1.27% in the 2010s) is very close to the latest estimate for the Amazon basin, where Allen and Pavelsky (2018)[143] found that streams and rivers occupy 1.33±0.02% of its drainage area.

We also notice that, although ephemeral streams are important sources of CO_2 emissions[144], they are not considered in this study due to data paucity. Ephemeral streams are widespread in north China with dry climate, especially the Huang-Huai-Hai and the NW China regions. Therefore, our CO_2 efflux estimates for river networks are likely conservative. Considering their short duration (e.g., less than 1 week[144]), we predict that the CO_2 efflux from ephemeral streams would not significantly bias our current estimates. However, more research that includes direct measurement of CO_2 evasion from ephemeral streams is needed to quantify this important efflux. In addition, if the sampling of CO_2 emissions is biased toward seasons when decomposition of aquatic organic matter fixed earlier in the year prevails (e.g., sampling lakes in autumn with decomposition of algal organic matter fixed earlier as summer GPP), there may be an overestimate of the net CO_2 efflux. Overall, the sampling in this study was evenly distributed over the non-growing (dry) and growing (wet) seasons. For example, the sampling in the 1980s was conducted at least on the monthly basis and it was usually on the weekly or even daily basis. For streams and rivers in the 2010s, the sampling at 83% of the sites was conducted in both the dry and wet seasons and only 2% of the sites were sampled in the dry season only. For lakes and reservoirs in the 2010s, the sampling at 61% of the sites was conducted in both the dry and wet seasons and 16% of the sites were sampled in the dry season only. Only two studies were carried out in autumn only (0.4% of all sampling sites in the 2010s). Therefore, we feel the sampling timing would not greatly affect the net CO_2 efflux estimate.

9. Implications for assessing China's and global carbon budgets

There is a large body of studies that examines the carbon budget in China, which are primarily concerned with carbon stock changes in its terrestrial biosphere and carbon emissions by burning fossil fuels[100, 145, 146, 147]. In comparison, CO_2 emissions from the corresponding inland waters were seldom examined across the country[80, 81]. The degassed CO_2 from inland waters originates partly from terrestrial respiration (soil CO_2) or from degradation of terrestrially derived organic matter within the aquatic column[98, 114]. Hence, inland water CO_2 emissions have great implications for better understanding of overall carbon fluxes and budget at the landscape scale. Although it remains difficult to identify the sources of inland water CO_2 evasion across large regions, we recognized that the efflux of CO_2 from inland waters that is derived from primary productivity within the aquatic ecosystem is not a net CO_2 flux to the atmosphere. Rather, it is simply the return of CO_2 to the atmosphere from aquatic ecosystems that had originally been fixed by photosynthesis within that ecosystem[148]. However, in most aquatic ecosystems it now appears that this cycle of photosynthesis and respiration within the ecosystem is not a dominant or even important driver of CO_2 evasion. Furthermore, this flux is largely balanced or is tending towards a net CO_2 sink on an annual time scale[39, 149, 150].

Recent studies conducted across different hydrologic and climatic settings suggest that the degassed CO_2 from inland waters is mostly derived from soil respiration (refs [77, 97] and references therein). This is particularly true for headwater streams that receive a much larger fraction from lateral transport of soil CO_2. To explore the implications of CO_2 evasion from inland waters across China for its terrestrial carbon sink estimates, we assumed that soil respiration contributed 90% of the evasion flux for headwater streams (first- and second-order streams) and 20% (range 17–25%; ref [20]) of the evasion flux for higher-order streams (3rd–8th order streams)[20, 97]. We also assumed that 20% of the evasion flux from lakes and reservoirs is derived from soil respiration, same as for higher-order streams. With these assumptions, we estimate that soil

respiration in China contributed 77 and 56 Tg C yr^{-1} to the CO_2 evasion flux from Chinese inland waters in the 1980s and 2010s, respectively. By subtraction from the total CO_2 evasion flux (138 Tg C yr^{-1} in the 1980s and 98 Tg C yr^{-1} in the 2010s), the remaining fluxes (i.e., 61 Tg C yr^{-1} in the 1980s and 42 Tg C yr^{-1} in the 2010s) are from other sources (e.g., primarily aquatic mineralization of terrestrially derived organic matter but also groundwater-derived inputs of inorganic carbon[57, 97]) not considered in the estimation of terrestrial carbon sink that relies on biomass and soil carbon inventories. Based on these remaining fluxes, we assessed their implications for China's carbon budget in the 1980s and 2010s.

The carbon balance of China's terrestrial ecosystems has been widely assessed. A pioneering work by Piao et al (2009)[100] reported an average net carbon sink of 177±73 Tg C yr^{-1} in Chinese terrestrial ecosystems in the 1980s and 1990s based on biomass accumulation techniques. Comparing our estimates to this carbon sink estimate suggests that CO_2 emissions from the Chinese inland waters offset the terrestrial carbon sink by 24–59% in the 1980s. Hence, ignoring the significant CO_2 emissions from inland waters could substantially overestimate the overall capability of China's terrestrial landscape in sequestrating carbon from the atmosphere.

With the initiation of several nationwide vegetation restoration programs since the early 1980s, China's forest coverage has increased from ~14% in the early 1980s to 21% in the 2010s[101]. As a result of the expanding forest area and the growth of established forests, the carbon sink by China's terrestrial ecosystems has substantially increased during these decades. Yet, there is still no carbon sink estimates regarding Chinese terrestrial ecosystems for the period 2010s. Fang et al (2018)[1] reported that Chinese terrestrial ecosystems had been sequestrating carbon at a rate of 201 Tg yr^{-1} during the period 2001–2010 by estimating the changes in biomass carbon stocks. Yue et al (2020)[151] and Ciais et al (2020)[152] recently provided an updated estimate of around 250 Tg C yr^{-1} for the period 2000–2009. If we use these fluxes (201–250 Tg C yr^{-1}) as conservative estimates for the period 2010s, we conclude that the overall capacity of carbon sequestration by China's terrestrial landscape was offset by 17–21% in the 2010s due to CO_2 emissions from inland waters. With continuously improving terrestrial ecosystems in the coming decades[153, 154], we acknowledge that the offset percentage due to inland water CO_2 evasion may steadily decline. Even so, we feel that the overall capability of carbon sequestration by China's terrestrial landscape will be greatly overestimated if CO_2 emissions from Chinese inland waters are not properly accounted for.

With respect to CO_2 emissions in industry and energy systems, China is the largest CO_2 emitter in the world, accounting for nearly one third of the global total CO_2 emissions from fossil-fuel combustion in 2015 (refs [1, 146]). The average carbon emission from fossil-fuel combustion in China, including mainland China, Hong Kong, Macau and Taiwan, was 550 Tg C yr^{-1} in the 1980s and 2762 Tg C yr^{-1} during the period 2010–2014 (ref [155]), of which 94–97% occurred in the mainland China. The degassed CO_2 from Chinese inland waters in the 1980s was equivalent to 19–31% (average: 25%) of the carbon emissions from fossil fuel combustion in that period. In comparison, the percentages declined to 2.8–4.2% (average: 4%) during the early 2010s due largely to rapid economic growth and soaring energy demand in the past decades. However, it is important to note that China's CO_2 emissions from industry and energy systems peaked in 2013 and started to decline since 2014 (ref [146]). Consequently, the relative importance of CO_2

emissions from China's inland water system is likely to increase again and it thus remains an important flux component in assessing China's overall carbon budget.

Quantifying CO_2 emission flux from global inland waters has received widespread attention over the past decades[15, 99, 156]. Raymond et al (2013)[14] estimated CO_2 emissions from global streams, rivers, lakes and reservoirs using data at 6708 sampling locations for streams and rivers and another 25,699 observations made in lakes and reservoirs. They estimated the global CO_2 efflux at 2.1 Pg C yr^{-1}, including 1.8 Pg C from streams and rivers and 0.3 Pg C from lakes and reservoirs, by combining an estimate for global inland water surface area with regional surface water pCO_2. This global efflux result is currently the most reliable estimate and has been widely cited. When compared with this global flux estimate, our CO_2 efflux results from Chinese inland waters represent 5–7% of the global estimate. In addition, previous flux estimates for China have been underestimated due to data paucity[14, 156]. For example, the total CO_2 efflux from Chinese inland waters was in the range of 48–55 Tg C yr^{-1} according to Raymond et al (2013)[14]. Therefore, replacing our estimates for China will cause a ~0.1 Pg increase over the previous global estimate of CO_2 emissions from inland waters.

10. Datasets used for estimating CO_2 emissions

The major datasets used in this study include:

1. Hydrologic Yearbooks, published by the Ministry of Water Resources of China (http://www.mwr.gov.cn/sj/) and the dataset is available at HKU DataHub (https://doi.org/10.25442/hku.13560452.v3).
2. Wind speed database of China, available at the National Meteorological Information Center, China Meteorological Administration (http://data.cma.cn).
3. Land cover database of China, available at the Resource and Environment Data Cloud Platform (1 km resolution for the 1980s and 2010s; http://www.resdc.cn) and the National Catalogue Service for Geographic Information (30 m resolution for the 2010s; http://www.webmap.cn).
4. HydroSHEDS database, available at https://www.hydrosheds.org.
5. Landsat and SRTM databases, available at the USGS (https://earthexplorer.usgs.gov).
6. SRTM database, available at the (http://srtm.csi.cgiar.org).
7. Fossil-Fuel CO_2 Emissions by Nation database, available at the Carbon Dioxide Information Analysis Center, available at https://cdiac.ess-dive.lbl.gov/trends/emis/tre_coun.html.
8. Chinese lakes and reservoirs in the 1980s are compiled in the Excel file 'Lakes and Reservoirs_1980s' and the dataset is available at HKU DataHub (https://doi.org/10.25442/hku.13560452.v3).
9. pCO_2 and CO_2 emissions in the 2010s are collected from the literature and compiled in the Excel file 'CO2 Dataset_2010s' and the dataset is available at HKU DataHub (https://doi.org/10.25442/hku.13560452.v3).

References for supplementary material

1. Fang J, Yu G, Liu L, Hu S, Chapin FS. Climate change, human impacts, and carbon sequestration in China. *Proceedings of the National Academy of Sciences* 2018, **115**(16)**:** 4015-4020.
2. Yu C, Huang X, Chen H, Godfray HCJ, Wright JS, Hall JW, *et al.* Managing nitrogen to restore water quality in China. *Nature* 2019, **567**(7749)**:** 516-520.

3. Wang H, Sun J. Variability of Northeast China river break-up date. *Advances in Atmospheric Sciences* 2009, **26**(4)**:** 701-706.
4. Wang SM, Dou HS. *Chinese Lake Catalogue*: Beijing, China, 1998.
5. Liu Y, Wu G, Guo R, Wan R. Changing landscapes by damming: the Three Gorges Dam causes downstream lake shrinkage and severe droughts. *Landscape Ecology* 2016, **31**(8)**:** 1883-1890.
6. Liu Y, Wu G, Zhao X. Recent declines in China's largest freshwater lake: trend or regime shift? *Environ Res Lett* 2013, **8**(1)**:** 014010.
7. Zhong Y, Zhong M, Feng W, Zhang Z, Shen Y, Wu D. Groundwater depletion in the West Liaohe River Basin, China and its implications revealed by GRACE and in situ measurements. *Remote Sensing* 2018, **10**(4)**:** 493.
8. Zhang G, Luo W, Chen W, Zheng G. A robust but variable lake expansion on the Tibetan Plateau. *Science Bulletin* 2019, **64:** 1306-1309.
9. Yang X, Lu X, Park E, Tarolli P. Impacts of climate change on lake fluctuations in the Hindu Kush-Himalaya-Tibetan Plateau. *Remote Sensing* 2019, **11**(9)**:** 1082.
10. Tao S, Fang J, Ma S, Cai Q, Xiong X, Tian D, *et al.* Changes in China's lakes: climate and human impacts. *National Science Review* 2020, **7**(1)**:** 132-140.
11. Zhang F, Tiyip T, Johnson VC, Ding J-l, Sun Q, Zhou M, *et al.* The influence of natural and human factors in the shrinking of the Ebinur Lake, Xinjiang, China, during the 1972–2013 period. *Environ Monit Assess* 2015, **187**(1)**:** 4128.
12. Abril G, Bouillon S, Darchambeau F, Teodoru C, Marwick T, Tamooh F, *et al.* Technical Note: Large overestimation of pCO_2 calculated from pH and alkalinity in acidic, organic-rich freshwaters. *Biogeosciences* 2015, **12:** 67-78.
13. Hunt C, Salisbury J, Vandemark D. Contribution of non-carbonate anions to total alkalinity and overestimation of pCO_2 in New England and New Brunswick rivers. *Biogeosciences* 2011, **8**(10)**:** 3069-3076.
14. Raymond PA, Hartmann J, Lauerwald R, Sobek S, McDonald C, Hoover M, *et al.* Global carbon dioxide emissions from inland waters. *Nature* 2013, **503**(7476)**:** 355-359.
15. Lauerwald R, Laruelle GG, Hartmann J, Ciais P, Regnier PA. Spatial patterns in CO_2 evasion from the global river network. *Global Biogeochemical Cycles* 2015, **29:** 534-554.
16. Ran L, Lu XX, Liu S. Dynamics of riverine CO_2 in the Yangtze River fluvial network and their implications for carbon evasion. *Biogeosciences* 2017, **14**(8)**:** 2183-2198.
17. Chen J, Wang FY, Xia XH, Zhang LT. Major element chemistry of the Changjiang (Yangtze River). *Chemical Geology* 2002, **187**(3-4)**:** 231-255.
18. Chen J, Wang F, Meybeck M, He D, Xia X, Zhang L. Spatial and temporal analysis of water chemistry records (1958–2000) in the Huanghe (Yellow River) basin. *Global Biogeochemical Cycles* 2005, **19**(3)**:** GB3016.
19. Zhang SR, Lu XX, Higgitt DL, Chen CTA, Sun HG, Han JT. Water chemistry of the Zhujiang (Pearl River): Natural processes and anthropogenic influences. *Journal of Geophysical Research* 2007, **112:** F01011.
20. Butman D, Raymond PA. Significant efflux of carbon dioxide from streams and rivers in the United States. *Nat Geosci* 2011, **4**(12)**:** 839-842.
21. Zheng M. *Saline lakes on the Qinghai-Tibet Plateau*: Beijing, China, 1989.
22. Robbins L, Hansen M, Kleypas J, Meylan S. CO_2calc: A user-friendly seawater carbon calculator for Windows, Mac OS X, and iOS (iPhone): US Geological Survey Open File Report 2010. Report No.: 2010-1280.

23. Millero FJ. The thermodynamics of the carbonate system in seawater. *Geochimica et Cosmochimica Acta* 1979, **43**(10)**:** 1651-1661.
24. Weyhenmeyer GA, Kosten S, Wallin MB, Tranvik LJ, Jeppesen E, Roland F. Significant fraction of CO_2 emissions from boreal lakes derived from hydrologic inorganic carbon inputs. *Nat Geosci* 2015, **8:** 933-936.
25. Müller D, Warneke T, Rixen T, Müller M, Jamahari S, *et al.* Lateral carbon fluxes and CO_2 outgassing from a tropical peat-draining river. *Biogeosciences* 2015, **12:** 5967-5979.
26. Ran L, Li L, Tian M, Yang X, Yu R, Zhao J, *et al.* Riverine CO_2 emissions in the Wuding River catchment on the Loess Plateau: Environmental controls and dam impoundment impact. *Journal of Geophysical Research: Biogeosciences* 2017, **122**(6)**:** 1439-1455.
27. Alin SR, Rasera MdFFL, Salimon CI, Richey JE, Holtgrieve GW, *et al.* Physical controls on carbon dioxide transfer velocity and flux in low-gradient river systems and implications for regional carbon budgets. *Journal of Geophysical Research* 2011, **116**(G1)**:** G01009.
28. Tian M, Yang X, Ran L, Su Y, Li L, Yu R, *et al.* Impact of land cover types on riverine CO_2 outgassing in the Yellow River Source Region. *Water* 2019, **11**(11)**:** 2243.
29. Lozovik P. Contribution of organic acid anions to the alkalinity of natural humic water. *Journal of Analytical Chemistry* 2005, **60**(11)**:** 1000-1004.
30. Nydahl AC, Wallin MB, Weyhenmeyer GA. No long-term trends in pCO2 despite increasing organic carbon concentrations in boreal lakes, streams, and rivers. *Global Biogeochemical Cycles* 2017, **31**(6)**:** 985-995.
31. Cai W-J, Wang Y, Hodson RE. Acid-base properties of dissolved organic matter in the estuarine waters of Georgia, USA. *Geochimica et Cosmochimica Acta* 1998, **62:** 473-483.
32. Davison W, Gardner MJ. Interlaboratory comparisons of the determination of pH in poorly buffered fresh waters. *Analytica chimica acta* 1986, **182:** 17-31.
33. Davison W, Woof C. Performance tests for the measurement of pH with glass electrodes in low ionic strength solutions including natural waters. *Analytical Chemistry* 1985, **57**(13)**:** 2567-2570.
34. Liu S, David B, Peter R. Evaluating CO_2 calculation error from organic alkalinity and pH measurement error in low ionic strength freshwaters. *Limnology and Oceanography: Methods* 2020, **18:** 606-622.
35. McDowell MJ, Johnson MS. Gas transfer velocities evaluated using carbon dioxide as a tracer show high streamflow to be a major driver of total CO_2 evasion flux for a headwater stream. *Journal of Geophysical Research: Biogeosciences* 2018, **123**(7)**:** 2183-2197.
36. Raymond PA, Zappa CJ, Butman D, Bott TL, Potter J, Mulholland P, *et al.* Scaling the gas transfer velocity and hydraulic geometry in streams and small rivers. *Limnology & Oceanography: Fluids & Environments* 2012, **2:** 41-53.
37. MacIntyre S, Fernandes Amaral JH, Barbosa PM, Cortés A, Forsberg BR, Melack JM. Turbulence and gas transfer velocities in sheltered flooded forests of the Amazon Basin. *Geophys Res Lett* 2019, **46**(16)**:** 9628-9636.
38. Jähne B, Heinz G, Dietrich W. Measurement of the diffusion-coefficients of sparingly soluble gases in water. *Journal of Geophysical Research* 1987, **92**(C10)**:** 10767-10776.
39. Horgby Å, Segatto PL, Bertuzzo E, Lauerwald R, Lehner B, Ulseth AJ, *et al.* Unexpected large evasion fluxes of carbon dioxide from turbulent streams draining the world's mountains. *Nature Communications* 2019, **10**(1)**:** 1-9.
40. Duvert C, Butman DE, Marx A, Ribolzi O, Hutley LB. CO_2 evasion along streams driven by groundwater inputs and geomorphic controls. *Nat Geosci* 2018, **11**(11)**:** 813-818.

41. Ulseth AJ, Hall RO, Boix Canadell M, Madinger HL, Niayifar A, Battin TJ. Distinct air–water gas exchange regimes in low- and high-energy streams. *Nat Geosci* 2019, **12:** 259-263.
42. Rabus B, Eineder M, Roth A, Bamler R. The shuttle radar topography mission—a new class of digital elevation models acquired by spaceborne radar. *ISPRS journal of photogrammetry and remote sensing* 2003, **57**(4)**:** 241-262.
43. Cole JJ, Caraco NF. Atmospheric exchange of carbon dioxide in a low-wind oligotrophic lake measured by the addition of SF_6. *Limnol Oceanogr* 1998, **43**(4)**:** 647-656.
44. Paranaíba JR, Barros N, Mendonça R, Linkhorst A, Isidorova A, Roland Fb*, et al.* Spatially resolved measurements of CO_2 and CH_4 concentration and gas-exchange velocity highly influence carbon-emission estimates of reservoirs. *Environmental Science & Technology* 2018, **52**(2)**:** 607-615.
45. Wanninkhof R. Relationship between wind speed and gas exchange. *Journal of Geophysical Research* 1992, **97**(25)**:** 7373-7382.
46. Wanninkhof R. Relationship between wind speed and gas exchange over the ocean revisited. *Limnology and Oceanography: Methods* 2014, **12**(6)**:** 351-362.
47. Raymond PA, Cole JJ. Gas exchange in rivers and estuaries: choosing a gas transfer velocity. *Estuaries* 2001, **24**(2)**:** 312-317.
48. Rudorff CM, Melack JM, MacIntyre S, Barbosa CC, Novo EM. Seasonal and spatial variability of CO_2 emission from a large floodplain lake in the lower Amazon. *Journal of Geophysical Research: Biogeosciences* 2011, **116**(G4).
49. Nightingale PD, Malin G, Law CS, Watson AJ, Liss PS, Liddicoat MI*, et al.* In situ evaluation of air-sea gas exchange parameterizations using novel conservative and volatile tracers. *Global Biogeochemical Cycles* 2000, **14**(1)**:** 373-387.
50. Crusius J, Wanninkhof R. Gas transfer velocities measured at low wind speed over a lake. *Limnology and Oceanography* 2003, **48**(3)**:** 1010-1017.
51. Chen X, Foley A, Zhang Z, Wang K, O'Driscoll K. An assessment of wind energy potential in the Beibu Gulf considering the energy demands of the Beibu Gulf Economic Rim. *Renewable and Sustainable Energy Reviews* 2020, **119:** 109605.
52. Yang X, Lu X. Drastic change in China's lakes and reservoirs over the past decades. *Scientific Reports* 2014, **4:** 6041.
53. Yang X, Lu X. Delineation of lakes and reservoirs in large river basins: An example of the Yangtze River Basin, China. *Geomorphology* 2013, **190:** 92-102.
54. Ministry of Water Resources and National Bureau of Statistics of China. *Bulletin of First National Census for Water*. China Water & Power Press: Beijing, China, 2013.
55. Piao S, Ciais P, Huang Y, Shen Z, Peng S, Li J*, et al.* The impacts of climate change on water resources and agriculture in China. *Nature* 2010, **467**(7311)**:** 43-51.
56. Strahler AN. Quantitative analysis of watershed geomorphology. *Eos, Transactions American Geophysical Union* 1957, **38**(6)**:** 913-920.
57. Ran L, Lu XX, Yang H, Li L, Yu R, Sun H*, et al.* CO_2 outgassing from the Yellow River network and its implications for riverine carbon cycle. *Journal of Geophysical Research: Biogeosciences* 2015, **120:** 1334-1347.
58. Gong L, Halldin S, Xu CY. Global-scale river routing—an efficient time-delay algorithm based on HydroSHEDS high-resolution hydrography. *Hydrological Processes* 2011, **25**(7)**:** 1114-1128.

59. Benstead JP, Leigh DS. An expanded role for river networks. *Nature Geosci* 2012, **5**(10)**:** 678-679.
60. Ministry of Water Resources of China. *Yearbook of China Water Resources*. China Water & Power Press: Beijing, China, 1990.
61. Ministry of Water Resources of China. *China Water Statistical Yearbook* China Water Power Press: Beijing, China, 2009.
62. Chen Y, Ye Z, Shen Y. Desiccation of the Tarim River, Xinjiang, China, and mitigation strategy. *Quaternary International* 2011, **244**(2)**:** 264-271.
63. Ran L, Lu XX. Redressing China's strategy of water resource exploitation. *Environ Manage* 2013, **51**(3)**:** 503-510.
64. Zhu Y, Chen Y, Ren L, Lü H, Zhao W, Yuan F, *et al.* Ecosystem restoration and conservation in the arid inland river basins of Northwest China: Problems and strategies. *Ecological Engineering* 2016, **94:** 629-637.
65. Zhao Y, Wei Y, Li S, Wu B. Downstream ecosystem responses to middle reach regulation of river discharge in the Heihe River Basin, China. *Hydrol Earth Syst Sc* 2016, **20:** 4469.
66. Li X, Chen Z, Fan X, Cheng Z. Hydropower development situation and prospects in China. *Renewable and Sustainable Energy Reviews* 2018, **82:** 232-239.
67. Bao Y, Gao P, He X. The water-level fluctuation zone of Three Gorges Reservoir — A unique geomorphological unit. *Earth-Science Reviews* 2015, **150:** 14-24.
68. National Bureau of Statistics of China. *China Statistical Yearbook*. China Statistics Press: Beijing China, 2011.
69. Zhang Q, Singh VP, Sun P, Chen X, Zhang Z, Li J. Precipitation and streamflow changes in China: Changing patterns, causes and implications. *Journal of Hydrology* 2011, **410**(3)**:** 204-216.
70. Li L, Yang S, Wang Z, Zhu X, Tang H. Evidence of warming and wetting climate over the Qinghai-Tibet Plateau. *Arctic, Antarctic, and Alpine Research* 2010, **42**(4)**:** 449-457.
71. Liu X, Chen B. Climatic warming in the Tibetan Plateau during recent decades. *Int J Climatol* 2000, **20**(14)**:** 1729-1742.
72. Li N, Liu J, Wang Z. Dynamics and driving force of lake changes in northeast China during 2000–2010. *Journal of Lake Sciences* 2014, **26**(4)**:** 545-551.
73. Liss PS, Slater PG. Flux of gases across the air-sea interface. *Nature* 1974, **247:** 181-184.
74. Liu S, Lu XX, Xia X, Yang X, Ran L. Hydrological and geomorphological control on CO_2 outgassing from low-gradient large rivers: an example of the Yangtze River system. *Journal of Hydrology* 2017, **550:** 26-41.
75. Wang X, Ma H, Li R, Song Z, Wu J. Seasonal fluxes and source variation of organic carbon transported by two major Chinese Rivers: The Yellow River and Changjiang (Yangtze) River. *Global Biogeochemical Cycles* 2012, **26**(2)**:** GB2025.
76. Read JS, Hamilton DP, Desai AR, Rose KC, MacIntyre S, Lenters JD, *et al.* Lake-size dependency of wind shear and convection as controls on gas exchange. *Geophys Res Lett* 2012, **39**(9).
77. Holgerson MA, Raymond PA. Large contribution to inland water CO_2 and CH_4 emissions from very small ponds. *Nat Geosci* 2016, **9:** 222-226.
78. Cheng G, Jin H. Permafrost and groundwater on the Qinghai-Tibet Plateau and in northeast China. *Hydrogeology Journal* 2013, **21**(1)**:** 5-23.

79. Guo Y, Song C, Wan Z, Lu Y, Qiao T, Tan W, *et al.* Dynamics of dissolved organic carbon release from a permafrost wetland catchment in northeast China. *Journal of Hydrology* 2015, **531:** 919-928.
80. Yue Y, Ni J, Ciais P, Piao S, Wang T, Huang M, *et al.* Lateral transport of soil carbon and land− atmosphere CO_2 flux induced by water erosion in China. *Proceedings of the National Academy of Sciences* 2016, **112:** 6617-6622.
81. Zhang H, Liu S, Yuan W, Dong W, Ye A, Xie X, *et al.* Inclusion of soil carbon lateral movement alters terrestrial carbon budget in China. *Scientific Reports* 2014, **4**(1)**:** 1-6.
82. Ran L, Lu X, Fang N, Yang X. Effective soil erosion control represents a significant net carbon sequestration. *Scientific Reports* 2018, **8**(1)**:** 12018.
83. Mei X, Dai Z, Darby SE, Gao S, Wang J, Jiang W. Modulation of extreme flood levels by impoundment significantly offset by floodplain loss downstream of the Three Gorges Dam. *Geophys Res Lett* 2018, **45**(7)**:** 3147-3155.
84. Qu B, Sillanpää M, Li C, Kang S, Stubbins A, Yan F, *et al.* Aged dissolved organic carbon exported from rivers of the Tibetan Plateau. *PloS one* 2017, **12**(5)**:** e0178166.
85. Ding J, Chen L, Ji C, Hugelius G, Li Y, Liu L, *et al.* Decadal soil carbon accumulation across Tibetan permafrost regions. *Nat Geosci* 2017, **10:** 420–424.
86. Li S, Bush RT, Santos IR, Zhang Q, Song K, Mao R, *et al.* Large greenhouse gases emissions from China's lakes and reservoirs. *Water Res* 2018, **147:** 13-24.
87. Knoll LB, Vanni MJ, Renwick WH, Dittman EK, Gephart JA. Temperate reservoirs are large carbon sinks and small CO_2 sources: Results from high-resolution carbon budgets. *Global Biogeochemical Cycles* 2013, **27**(1)**:** 52-64.
88. Morin TH, Rey-Sánchez AC, Vogel CS, Matheny AM, Kenny WT, Bohrer G. Carbon dioxide emissions from an oligotrophic temperate lake: An eddy covariance approach. *Ecological Engineering* 2018, **114:** 25-33.
89. Loken LC, Crawford JT, Schramm PJ, Stadler P, Desai AR, Stanley EH. Large spatial and temporal variability of carbon dioxide and methane in a eutrophic lake. *Journal of Geophysical Research: Biogeosciences* 2019, **124**(7)**:** 2248-2266.
90. Rocher-Ros G, Giesler R, Lundin E, Salimi S, Jonsson A, Karlsson J. Large lakes dominate CO_2 evasion from lakes in an arctic catchment. *Geophys Res Lett* 2017, **44:** 12,254-212,261.
91. National Bureau of Statistics. Communiqué of the National Bureau of Statistics of People's Republic of China on Major Figures of the 2010 Population Census. 2013. Available from: http://www.stats.gov.cn/
92. Schade JD, Bailio J, McDowell WH. Greenhouse gas flux from headwater streams in New Hampshire, USA: Patterns and drivers. *Limnology and Oceanography* 2016.
93. Wallin MB, Grabs T, Buffam I, Laudon H, Ågren A, Öquist MG, *et al.* Evasion of CO_2 from streams–The dominant component of the carbon export through the aquatic conduit in a boreal landscape. *Global Change Biology* 2013, **19**(3)**:** 785-797.
94. Kokic J, Wallin MB, Chmiel HE, Denfeld BA, Sobek S. Carbon dioxide evasion from headwater systems strongly contributes to the total export of carbon from a small boreal lake catchment. *Journal of Geophysical Research: Biogeosciences* 2015, **120**(1)**:** 13-28.
95. Marescaux A, Thieu V, Garnier J. Carbon dioxide, methane and nitrous oxide emissions from the human-impacted Seine watershed in France. *Sci Total Environ* 2018, **643:** 247-259.

96. Duvert C, Bossa M, Tyler KJ, Wynn JG, Munksgaard NC, Bird MI*, et al.* Groundwater-derived DIC and carbonate buffering enhance fluvial CO_2 evasion in two Australian tropical rivers. *Journal of Geophysical Research: Biogeosciences* 2019, **124**(2)**:** 312-327.
97. Horgby Å, Boix Canadell M, Ulseth AJ, Vennemann TW, Battin TJ. High-resolution spatial sampling identifies groundwater as driver of CO_2 dynamics in an Alpine stream network. *Journal of Geophysical Research: Biogeosciences* 2019, **124:** 1961-1976.
98. Crawford JT, Loken LC, Stanley EH, Stets EG, Dornblaser MM, Striegl RG. Basin scale controls on CO_2 and CH_4 emissions from the Upper Mississippi River. *Geophys Res Lett* 2016, **43**(5)**:** 1973-1979.
99. Drake TW, Raymond PA, Spencer RG. Terrestrial carbon inputs to inland waters: A current synthesis of estimates and uncertainty. *Limnology and Oceanography Letters* 2018, **3:** 132-142.
100. Piao S, Fang J, Ciais P, Peylin P, Huang Y, Sitch S*, et al.* The carbon balance of terrestrial ecosystems in China. *Nature* 2009, **458**(7241)**:** 1009-1013.
101. Tang X, Zhao X, Bai Y, Tang Z, Wang W, Zhao Y*, et al.* Carbon pools in China's terrestrial ecosystems: New estimates based on an intensive field survey. *Proceedings of the National Academy of Sciences* 2018, **115**(16)**:** 4021-4026.
102. Deng L, Liu G-b, Shangguan Z-p. Land-use conversion and changing soil carbon stocks in China's 'Grain-for-Green'Program: a synthesis. *Global Change Biology* 2014, **20**(11)**:** 3544-3556.
103. Williams CJ, Yamashita Y, Wilson HF, Jaffé R, Xenopoulos MA. Unraveling the role of land use and microbial activity in shaping dissolved organic matter characteristics in stream ecosystems. *Limnology and Oceanography* 2010, **55**(3)**:** 1159-1171.
104. Masese FO, Salcedo-Borda JS, Gettel GM, Irvine K, McClain ME. Influence of catchment land use and seasonality on dissolved organic matter composition and ecosystem metabolism in headwater streams of a Kenyan river. *Biogeochemistry* 2017, **132:** 1-22.
105. Fang M, Chan CK, Yao X. Managing air quality in a rapidly developing nation: China. *Atmos Environ* 2009, **43**(1)**:** 79-86.
106. Bodmer P, Heinz M, Pusch M, Singer G, Premke K. Carbon dynamics and their link to dissolved organic matter quality across contrasting stream ecosystems. *Sci Total Environ* 2016, **553:** 574-586.
107. Amann T, Weiss A, Hartmann J. Carbon dynamics in the freshwater part of the Elbe estuary, Germany: Implications of improving water quality. *Estuarine, Coastal and Shelf Science* 2012, **107:** 112-121.
108. Xie H, Chen Q, Wang W, He Y. Analyzing the green efficiency of arable land use in China. *Technological Forecasting and Social Change* 2018, **133:** 15-28.
109. Larson C. Losing arable land, China faces stark choice: adapt or go hungry. American Association for the Advancement of Science; 2013.
110. Lei D, Shangguan Z-P, Rui L. Effects of the grain-for-green program on soil erosion in China. *International Journal of Sediment Research* 2012, **27**(1)**:** 120-127.
111. Prairie YT, Alm J, Beaulieu J, Barros N, Battin T, Cole J*, et al.* Greenhouse gas emissions from freshwater reservoirs: what does the atmosphere see? *Ecosystems* 2018, **21**(5)**:** 1058-1071.
112. Barros N, Cole JJ, Tranvik LJ, Prairie YT, Bastviken D, Huszar VL*, et al.* Carbon emission from hydroelectric reservoirs linked to reservoir age and latitude. *Nat Geosci* 2011, **4**(9)**:** 593-596.

113. Almeida RM, Nobrega GN, Junger PC, Figueiredo AV, Andrade AS, de Moura CG, *et al.* High primary production contrasts with intense carbon emission in a eutrophic tropical reservoir. *Frontiers in Microbiology* 2016, **7:** 717.
114. Tranvik LJ, Downing JA, Cotner JB, Loiselle SA, Striegl RG, Ballatore TJ, *et al.* Lakes and reservoirs as regulators of carbon cycling and climate. *Limnology and Oceanography* 2009, **54**(6)**:** 2298-2314.
115. Bevelhimer MS, Stewart AJ, Fortner AM, Phillips JR, Mosher JJ. CO_2 is dominant greenhouse gas emitted from six hydropower reservoirs in southeastern United States during peak summer emissions. *Water* 2016, **8**(1)**:** 15.
116. Kosten S, van den Berg S, Mendonça R, Paranaíba JR, Roland F, Sobek S, *et al.* Extreme drought boosts CO_2 and CH_4 emissions from reservoir drawdown areas. *Inland Waters* 2018, **8**(3)**:** 329-340.
117. Chen H, Wu Y, Yuan X, Gao Y, Wu N, Zhu D. Methane emissions from newly created marshes in the drawdown area of the Three Gorges Reservoir. *Journal of Geophysical Research: Atmospheres (1984–2012)* 2009, **114:** D18301.
118. Junger PC, Dantas FdCC, Nobre RLG, Kosten S, Venticinque EM, Araújo FdC, *et al.* Effects of seasonality, trophic state and landscape properties on CO_2 saturation in low-latitude lakes and reservoirs. *Sci Total Environ* 2019, **664:** 283-295.
119. Li S, Zhang Q, Bush RT, Sullivan LA. Methane and CO_2 emissions from China's hydroelectric reservoirs: a new quantitative synthesis. *Environmental Science and Pollution Research* 2015, **22**(7)**:** 5325-5339.
120. Yang L, Lu F, Wang X. Measuring Greenhouse Gas Emissions From China's Reservoirs. *Eos, Transactions American Geophysical Union* 2014, **95**(1)**:** 1-2.
121. Zarfl C, Lumsdon AE, Berlekamp J, Tydecks L, Tockner K. A global boom in hydropower dam construction. *Aquatic Sciences* 2015, **77**(1)**:** 161-170.
122. Pacheco FS, Roland F, Downing JA. Eutrophication reverses whole-lake carbon budgets. *Inland waters* 2014, **4**(1)**:** 41-48.
123. Ma J, Li H. Preliminary discussion on eutrophication status of lakes, reservoirs and reivers in China and overseas. *Resources and Environment in the Yangtze Valley* 2002, **11**(6)**:** 575-578.
124. Yang J, Yu X, Liu L, Zhang W, Guo P. Algae community and trophic state of subtropical reservoirs in southeast Fujian, China. *Environmental Science and Pollution Research* 2012, **19**(5)**:** 1432-1442.
125. Huang J, Zhang Y, Arhonditsis GB, Gao J, Chen Q, Wu N, *et al.* How successful are the restoration efforts of China's lakes and reservoirs? *Environment International* 2019, **123:** 96-103.
126. Yang L, Lu F, Wang X, Duan X, Song W, Sun B, *et al.* Spatial and seasonal variability of diffusive methane emissions from the Three Gorges Reservoir. *Journal of Geophysical Research: Biogeosciences* 2013, **118**(2)**:** 471-481.
127. Zheng H, Zhao X, Zhao T, Chen F, Xu W, Duan X, *et al.* Spatial–temporal variations of methane emissions from the Ertan hydroelectric reservoir in southwest China. *Hydrological Processes* 2011, **25**(9)**:** 1391-1396.
128. Kumar A, Yang T, Sharma MP. Long-term prediction of greenhouse gas risk to the Chinese hydropower reservoirs. *Sci Total Environ* 2019, **646:** 300-308.

129. Peter H, Singer GA, Preiler C, Chifflard P, Steniczka G, Battin TJ. Scales and drivers of temporal pCO_2 dynamics in an Alpine stream. *Journal of Geophysical Research: Biogeosciences* 2014, **119**(6)**:** 1078-1091.
130. Le C, Zha Y, Li Y, Sun D, Lu H, Yin B. Eutrophication of lake waters in China: cost, causes, and control. *Environ Manage* 2010, **45**(4)**:** 662-668.
131. Food and Agriculture Organization of the United Nations. FAOSTAT 2017**:** http://www.fao.org/faostat/en/#data/RFN.
132. Han D, Currell MJ, Cao G. Deep challenges for China's war on water pollution. *Environmental Pollution* 2016, **218:** 1222-1233.
133. Shao M, Tang X, Zhang Y, Li W. City clusters in China: air and surface water pollution. *Front Ecol Environ* 2006, **4**(7)**:** 353-361.
134. Xiao Q, Duan H, Qi T, Hu Z, Liu S, Zhang M, *et al.* Environmental investments decreased partial pressure of CO_2 in a small eutrophic urban lake: Evidence from long-term measurements. *Environmental Pollution* 2020, **263:** 114433.
135. Xiao Q, Xu X, Duan H, Qi T, Qin B, Lee X, *et al.* Eutrophic Lake Taihu as a significant CO_2 source during 2000–2015. *Water Res* 2020, **170:** 115331.
136. Yang H, Xing Y, Xie P, Ni L, Rong K. Carbon source/sink function of a subtropical, eutrophic lake determined from an overall mass balance and a gas exchange and carbon burial balance. *Environmental Pollution* 2008, **151**(3)**:** 559-568.
137. Taylor JR. *An introduction to error analysis: the study of uncertainties in physical measurements*. University science books, 1997.
138. Team RC. R: A language and environment for statistical computing. 2013.
139. Bao Y. The study of hydrochemical characteristics and caron cycles in the Yarlung Zangbo River Basin. PhD thesis, China Institute of Water Resources and Hydropower Research, Beijing, 2019.
140. Wen Z, Song K, Zhao Y, Jin X. Carbon dioxide and methane supersaturation in lakes of semi-humid/semi-arid region, Northeastern China. *Atmos Environ* 2016, **138:** 65-73.
141. Crawford JT, Dornblaser MM, Stanley EH, Clow DW, Striegl RG. Source limitation of carbon gas emissions in high-elevation mountain streams and lakes. *Journal of Geophysical Research: Biogeosciences* 2015, **120**(5)**:** 952-964.
142. Allen GH, Pavelsky TM, Barefoot EA, Lamb MP, Butman D, Tashie A, *et al.* Similarity of stream width distributions across headwater systems. *Nature communications* 2018, **9:** 610.
143. Allen GH, Pavelsky TM. Global extent of rivers and streams. *Science* 2018, **361:** 585-588.
144. Von Schiller D, Datry T, Corti R, Foulquier A, Tockner K, Marcé R, *et al.* Sediment respiration pulses in intermittent rivers and ephemeral streams. *Global Biogeochemical Cycles* 2019, **33**(10)**:** 1251-1263.
145. Lun F, Li W, Liu Y. Complete forest carbon cycle and budget in China, 1999–2008. *Forest Ecology and Management* 2012, **264:** 81-89.
146. Guan D, Meng J, Reiner DM, Zhang N, Shan Y, Mi Z, *et al.* Structural decline in China's CO_2 emissions through transitions in industry and energy systems. *Nat Geosci* 2018, **11**(8)**:** 551-555.
147. Zhu J, Hu H, Tao S, Chi X, Li P, Jiang L, *et al.* Carbon stocks and changes of dead organic matter in China's forests. *Nature Communications* 2017, **8**(1)**:** 1-10.
148. Cole JJ, Prairie YT, Caraco NF, McDowell WH, Tranvik LJ, Striegl RG, *et al.* Plumbing the global carbon cycle: Integrating inland waters into the terrestrial carbon budget. *Ecosystems* 2007, **10**(1)**:** 171-184.

149. Mendonça R, Müller RA, Clow D, Verpoorter C, Raymond P, Tranvik LJ, *et al.* Organic carbon burial in global lakes and reservoirs. *Nature Communications* 2017, **8**(1)**:** 1694.
150. Bogard MJ, Kuhn CD, Johnston SE, Striegl RG, Holtgrieve GW, Dornblaser MM, *et al.* Negligible cycling of terrestrial carbon in many lakes of the arid circumpolar landscape. *Nat Geosci* 2019, **12**(3)**:** 180-185.
151. Yue C, Ciais P, Houghton RA, Nassikas AA. Contribution of land use to the interannual variability of the land carbon cycle. *Nature Communications* 2020, **11**(1)**:** 3170.
152. Ciais P, Yao Y, Gasser T, Baccini A, Wang Y, Lauerwald R, *et al.* Empirical estimates of regional carbon budgets imply reduced global soil heterotrophic respiration. *National Science Review* 2020.
153. Hu H, Wang S, Guo Z, Xu B, Fang J. The stage-classified matrix models project a significant increase in biomass carbon stocks in China's forests between 2005 and 2050. *Scientific reports* 2015, **5:** 11203.
154. Lu F, Hu H, Sun W, Zhu J, Liu G, Zhou W, *et al.* Effects of national ecological restoration projects on carbon sequestration in China from 2001 to 2010. *Proceedings of the National Academy of Sciences* 2018, **115**(16)**:** 4039-4044.
155. Boden T, Marland G, Andres R. Global, regional, and national fossil-fuel CO_2 emissions (1751-2014)(v. 2017): Carbon Dioxide Information Analysis Center, Oak Ridge National Laboratory, U.S. Department of Energy, Oak Ridge, Tenn., U.S.A; 2017.
156. Regnier P, Friedlingstein P, Ciais P, Mackenzie FT, Gruber N, Janssens IA, *et al.* Anthropogenic perturbation of the carbon fluxes from land to ocean. *Nat Geosci* 2013, **6:** 597-607.
